# Burden and Trends of Genitourinary Cancers Across the Americas: A GBD 2023 Analysis of Regional Socioeconomic Gradients

**DOI:** 10.3390/cancers18122016

**Published:** 2026-06-22

**Authors:** José Guzmán-Esquivel, Gustavo A. Hernández-Fuentes, Kayim Pineda-Urbina, Janet Diaz-Martinez, Carlos M. Hernandez-Suarez, Jesús Venegas-Ramírez, Gabriel Ceja-Espíritu, Iram P. Rodríguez-Sánchez, Margarita L. Martinez-Fierro, Idalia Garza-Veloz, Fabian Rojas-Larios, Alejandrina Rodríguez-Hernandez, Daniel A. Montes-Galindo, Iván Delgado-Enciso

**Affiliations:** 1 Clinical Epidemiology Research Unit, Mexican Institute of Social Security (IMSS), Villa de Alvarez 29883, Colima, Mexico; jose.esquivel@imss.gob.mx; 2 Department of Molecular Medicine, School of Medicine, University of Colima, Colima 28040, Mexico; ghfuentes@ucol.mx (G.A.H.-F.); gcejae11@ucol.mx (G.C.-E.); frojas@ucol.mx (F.R.-L.); arodrig@ucol.mx (A.R.-H.); 3 State Cancerology Institute of Colima, Health Services of the Mexican Social Security Institute for Welfare (IMSS-BIENESTAR), Colima 28085, Mexico; 4 Faculty of Chemical Sciences, University of Colima, Coquimatlan 28400, Mexico; kpineda@ucol.mx (K.P.-U.); daniel_montes@ucol.mx (D.A.M.-G.); 5 Research Center in Minority Institutions, Florida International University (FIU-RCMI), Miami, FL 33199, USA; jdimarti@fiu.edu; 6 Department of Dietetics & Nutrition, Robert Stempel College of Public Health & Social Work, Florida International University (FIU-RCMI), Miami, FL 33199, USA; 7 Coordinación General de Investigación, University of Colima, Colima 28040, Mexico; cmh1@cornell.edu; 8 Department of Nephrology, Instituto Mexicano del Seguro Social (IMSS), Hospital de Zona No. 1, Villa de Álvarez 28984, Colima, Mexico; jesus.venegas@imss.gob.mx; 9 Molecular and Structural Physiology Laboratory, School of Biological Sciences, Universidad Autónoma de Nuevo León, San Nicolás de los Garza 66455, Nuevo León, Mexico; 10 Molecular Medicine Laboratory, Academic Unit of Human Medicine and Health Sciences, Autonomous University of Zacatecas, Zacatecas 98160, Mexico; margaritamf@uaz.edu.mx (M.L.M.-F.); idaliagv@uaz.edu.mx (I.G.-V.)

**Keywords:** Americas, Global Burden of Disease, genitourinary cancers, prostate cancer, socioeconomic disparities, social determinants of health

## Abstract

Background/Objectives: Genitourinary cancers represent a major and growing source of cancer burden worldwide; however, important disparities persist across the Americas. This study aimed to evaluate the incidence, mortality, and disability burden of prostate, testicular, bladder, and kidney cancers across 38 countries and territories using Global Burden of Disease (GBD) 2023 estimates, with emphasis on temporal trends and sociodemographic inequalities. Methods: A descriptive ecological study was conducted using Global Burden of Disease (GBD) 2023 estimates. Age-standardized incidence, mortality, and disability-adjusted life year (DALY) rates per 100,000 population were analyzed for prostate, bladder, kidney, and testicular cancers. Burden estimates were obtained from GBD 2023 data, and temporal trend analyses were conducted using age-standardized rates from 2000–2023. Temporal trends were assessed using weighted log-linear regression to estimate annual percentage changes (APCs) based on age-standardized rates from 2000–2023. Results: In 2023, prostate cancer accounted for the greatest genitourinary cancer burden across the Americas, with high incidence concentrated in high-income North America, whereas mortality and DALY rates were disproportionately elevated in Latin America and the Caribbean. Across all cancer types, high-SDI regions consistently exhibited higher incidence but more favorable mortality and disability profiles. Testicular cancer incidence increased across all SDI quintiles, although mortality reductions were mainly observed in high-SDI settings. Bladder and kidney cancers demonstrated similar epidemiological patterns, with declining mortality trends in high-income regions but persistent or increasing burden in lower-SDI countries. Mortality-to-incidence disparities remained substantial across Latin America and the Caribbean, which may reflect differences in healthcare resources, early detection, treatment availability, or other contextual factors not directly captured in the GBD database. National extremes included Bermuda prostate ASIR 170.63 and Dominica DALYs 1423.30 per 100,000. Conclusions: The burden of genitourinary cancers across the Americas remains strongly associated with socioeconomic inequalities. Although higher-resource settings have achieved important reductions in mortality and disability, these gains have not been equitably distributed across the region. Strengthening health system capacity, improving early diagnosis, and ensuring equitable access to evidence-based cancer care are essential to reduce avoidable mortality and improve long-term outcomes throughout the Americas.

## Simple Summary

Genitourinary cancers, including prostate, bladder, kidney, and testicular cancers, are an increasing public health challenge across the Americas. However, the burden of these malignancies is not evenly distributed. Countries with higher socioeconomic development tend to report more diagnosed cases, which may reflect greater access to screening and diagnostic technologies, while lower-resource settings experience disproportionately higher mortality and disability. Using data from the Global Burden of Disease 2023 study, we evaluated long-term trends in incidence, mortality, and disability-adjusted life years across 38 countries and territories from 2000 to 2023. Our findings reveal persistent and widening inequalities, particularly in Latin America and the Caribbean, which may reflect differences in healthcare resources and cancer care delivery. These findings support the need to evaluate strategies aimed at improving equitable access to cancer diagnosis and care across the Americas.

## 1. Introduction

Genitourinary cancers, including malignancies of the prostate, bladder, kidney, and testis, represent a substantial and increasingly relevant component of the global cancer burden, particularly within the framework of the Global Burden of Disease (GBD) study [1,2]. Among these, prostate cancer stands out as the most frequently diagnosed and a leading cause of cancer-related mortality among men worldwide, while testicular, bladder, and kidney cancers contribute distinct epidemiological and clinical challenges across different age groups and risk profiles [2].

The Americas encompasses 38 countries and territories characterized by pronounced socioeconomic heterogeneity, which plays a central role in shaping cancer epidemiology across the region [3]. Previous evidence suggests a consistent but paradoxical pattern: higher-income settings tend to report elevated age-standardized incidence rates, which may be associated with greater access to diagnostic technologies and more intensive detection practices, whereas low- and middle-income countries (LMICs) often experience less favorable outcomes [4,5] which may be consistent with persistent inequities in timely diagnosis, treatment access, healthcare system capacity, and other contextual factors [6]. This divergence between incidence and outcomes underscores the importance of examining cancer burden beyond occurrence alone.

Ongoing demographic and epidemiological transitions, including population ageing, urbanization, and increased life expectancy [7], are further amplifying the cancer burden across the Americas [8,9]. These shifts not only increase the absolute number of cancer cases but also interact with structural determinants of health, such as income inequality, education, and healthcare infrastructure, thereby exacerbating disparities in cancer outcomes across countries and regions [10,11,12,13].

The Sociodemographic Index (SDI), a composite measure of income, education, and fertility, has emerged as a key indicator for understanding these patterns [14]. While higher SDI settings generally exhibit increased incidence due to more widespread detection, they also tend to achieve better survival outcomes, reflected in lower mortality-to-incidence ratios and reduced DALY rates [15]. In contrast, lower-SDI settings frequently experience a disproportionate burden of advanced disease and premature mortality, suggesting that differences in healthcare access and quality may contribute to the observed disparities, although these factors could not be directly evaluated in the present study [9].

Despite growing recognition of these inequalities, a comprehensive and up-to-date assessment integrating incidence, mortality, and DALYs across countries, regions, and SDI strata in the Americas—using the most recent GBD 2023 estimates—remains lacking. In particular, it is still unclear whether the apparent decoupling between incidence and mortality is consistent across all genitourinary cancers, how these patterns have evolved over time, and to what extent unfavorable outcome gradients persist or widen in Latin America and the Caribbean.

Although several GBD-based studies have evaluated individual genitourinary cancers at global or national levels, comprehensive analyses simultaneously examining prostate, testicular, kidney, and bladder cancers across the Americas using the most recent GBD 2023 estimates remain limited [16,17]. Furthermore, few studies have evaluated temporal trends together with Sociodemographic Index gradients and mortality-to-incidence ratios across the region. Given the substantial socioeconomic heterogeneity of the Americas, updated assessments are needed to better characterize contemporary disparities in genitourinary cancer burden and outcomes [16,17,18].

Therefore, the aim of this study was to quantify and compare age-standardized incidence, mortality, and DALY rates for prostate, testicular, bladder, and kidney cancers across 38 countries and territories in the Americas using GBD 2023 estimates. Additionally, we evaluated temporal trends from 2000 to 2023 and examined how these patterns vary by SDI level and geographic region. By integrating multiple dimensions of disease burden, this study seeks to provide a more comprehensive understanding of persistent disparities and to generate evidence that can inform more equitable cancer control strategies across the region.

## 2. Materials and Methods

### 2.1. Data Source and GBD Framework

An ecological study was conducted using population-level aggregated data from the Global Burden of Disease database. Estimates from the Global Burden of Diseases, Injuries, and Risk Factors Study (GBD) 2023 were used [19,20]. GBD researchers gathered health data from civil registries, national censuses, household surveys, cancer registries, hospital discharge codes, published studies, and government reports—then applied consistent analytic methods across all sources. Health loss causes within GBD are organized into hierarchical groupings with increasingly granular levels of diseases, injuries, and disability sequelae [19,20]. The main objective is to provide a robust evidence base for health policy formulation and efficient resource allocation, encapsulated in the vision: “All people live long and healthy lives” [21]. Although GBD estimates were available from 1990 to 2023, APC analyses were restricted to 2000–2023 to evaluate contemporary temporal trends and improve comparability across locations.

All data used in this study are publicly available and de-identified, ensuring full compliance with ethical standards. Detailed information on GBD components, causes, risks, and locations is accessible via the Global Health Data Exchange (https://www.healthdata.org/data-tools-practices/data-sources) (accessed on 15 May 2025). Complementary tools from the Institute for Health Metrics and Evaluation (IHME), such as GBD Compare and GBD Results, enable in-depth exploration of estimates and methods.

### 2.2. Study Outcomes and Metrics

Age-standardized incidence, mortality, and disability-adjusted life years (DALYs) per 100,000 population, with 95% uncertainty intervals (UIs) were extracted for prostate, kidney, bladder, and testicular cancers from the GBD 2023 database for 2000 and 2023, with temporal trend analyses conducted from 2000 to 2023. Mortality estimates were modelled using the Cause of Death Ensemble model (CODEm) [22]. DALYs were calculated as the sum of years of life lost (YLLs) and years lived with disability (YLDs). All estimates were reported as counts or rates for all ages or age-standardized values. Means and UIs were derived from 1000 posterior draws. Detailed counts, age-standardized rates, and trend estimates used in this study are provided in Supplementary Tables S1–S13.

### 2.3. Temporal Trend Analysis

Temporal trends were assessed using annual percentage changes (APCs) [23,24]. For each cancer type, age-standardized incidence, mortality, and DALY rates from 2000 to 2023 were modeled using weighted log-linear regression. Specifically, the natural logarithm of each age-standardized rate was regressed against calendar year according to the model: *log(*$y_{t}$*) =* α *+ λt,* where $y_{t}$ represents the age-standardized rate in year t, α is the intercept, and λ is the annual rate of change. Weighted least squares (WLS) regression was applied, with each annual observation weighted by the inverse squared width of its corresponding 95% uncertainty interval obtained from GBD 2023 estimates $\left[1/(upper-lower{)}^{2}\right]$. This approach assigns greater weight to estimates with higher precision.

APCs were calculated as $(e^{\lambda }-1)\times 100$, representing the average percentage change per year. Ninety-five percent confidence intervals (95% CIs) for APC estimates were derived from the weighted standard error of the regression slope. Negative APC values indicate decreasing trends over time, whereas positive APC values indicate increasing trends. This approach was chosen to provide a consistent measure of long-term temporal change across countries, regions, and Sociodemographic Index (SDI) categories [25]. The methodological approach used to estimate APC is described in detail in Supplementary Table S14.

### 2.4. Mortality-to-Incidence Ratio Analysis

The mortality-to-incidence ratio (MIR) was calculated as the quotient between the age-standardized mortality rate (ASMR) and the age-standardized incidence rate (ASIR): MIR = ASMR/ASIR.

MIRs were estimated for prostate, testicular, kidney, and bladder cancers using GBD 2023 age-standardized rates. Calculations were performed at the global, regional, and Sociodemographic Index (SDI) levels. Lower MIR values were interpreted as indicative of more favorable cancer outcomes and may be associated with greater access to early diagnosis and effective treatment, whereas higher MIR values may reflect less favorable survival patterns and potential inequalities in healthcare access (Supplementary Table S16).

### 2.5. Geographic Locations and SDI

GBD generates estimates for 204 countries and territories grouped into 21 regions. The Americas region includes 38 countries and territories (Antigua and Barbuda, Argentina, Bahamas, Barbados, Belize, Bermuda, Bolivia, Canada, Chile, Colombia, Costa Rica, Cuba, Dominica, Dominican Republic, Ecuador, El Salvador, Grenada, Greenland, Guatemala, Guyana, Haiti, Honduras, Jamaica, Mexico, Nicaragua, Panama, Paraguay, Puerto Rico, Peru, Saint Kitts and Nevis, Saint Lucia, Saint Vincent and the Grenadines, Suriname, Trinidad and Tobago, United States of America, Uruguay, Venezuela). Analyses were conducted in the country, regional (high-income North America; Latin America and the Caribbean), and SDI levels.

The Sociodemographic Index (SDI) is a composite indicator reflecting underlying social and economic conditions influencing health outcomes, calculated as the geometric mean of the total fertility rate under age 25 (TFU25), mean education for those aged 15+ years (EDU15+), and per capita income, scaled from 0 to 100. Locations were classified into five SDI quintiles (low SDI, low-middle SDI, middle SDI, high-middle SDI, and high SDI) according to the GBD 2023 classification framework (Supplementary Table S1) [26]. Graphical presentations were executed using R programming language (version 4.4.1) [27].

### 2.6. Period of Analysis

Number and age-standardized rates of incidence, mortality, and DALYs were estimated for the Americas region using GBD 2023 estimates, with percentage change rates for 2000–2023. Results were compared across Americas countries, with specific analyses at global, SDI, high-income North America, Latin America and the Caribbean, and SDI levels. Findings were stratified by sex (kidney and bladder cancers) or males only (prostate and testis), by location and year. Analyses were conducted using Stata (version 13.1) and R (version 4.4.1).

### 2.7. GBD Cause List and Coding

GBD causes and sequelae are hierarchically organized. Level 1 includes communicable, maternal, neonatal, and nutritional diseases (group 1); non-communicable diseases (group 2); and injuries (group 3). Neoplasms, including genitourinary cancers, fall under non-communicable diseases at level 3, coded per International Statistical Classification of Diseases and Related Health Problems, 10th Revision (ICD-10): prostate (C61), kidney (C64), bladder (C67), testis (C62) [28].

### 2.8. GATHER Compliance

This study adheres to the Guidelines for Accurate and Transparent Health Estimates Reporting (GATHER). Analytical procedures and data sources have been documented; the completed GATHER checklist is in (Supplementary Table S15) (http://gather-statement.org/) [29].

### 2.9. Input Data Sources Overview

GBD 2023 incorporates extensive input data sources (surveys, censuses, vital statistics, health-related data) to estimate morbidity, attributable risk, and cause-specific mortality for 204 locations from 1990–2023. Genitourinary cancer inputs included hospital registries, emergency services, insurance claims, surveys, and vital registration systems globally. Methodological details are published previously [21]. Verbal autopsy via SmartVA-Analyze (Tariff 2.0 method) addressed mortality data gaps in countries lacking complete vital registration. Input data are accessible via GHDx (https://ghdx.healthdata.org/gbd-2023/sources [30]) (accessed on 20 May 2025); (https://ghdx.healthdata.org/search/site/cancer) (accessed on 20 May 2025) [31]. Statistical code is provided in GHDx [32]. The population sizes of the countries in the Americas and their corresponding 2021 SDI quintiles have been previously described [33].

Bladder and prostate cancer incidence were estimated by multiplying procedure proportions by oncology case data, input into DisMod-MR 2.1 for prevalence modelling. CoD database mortality fed into CODEm, with CoDCorrect adjustments incorporating causal fraction uncertainty.

Mortality estimates were generated using the Cause of Death Ensemble Model (CODEm), a statistical framework that combines multiple predictive models and covariates to improve estimation accuracy across locations with varying levels of data completeness. Incidence and prevalence estimates were informed by DisMod-MR 2.1, a Bayesian meta-regression tool that synthesizes available epidemiological data and enforces internal consistency among disease parameters. Both modeling approaches contribute to uncertainty propagation, which is reflected in the 95% uncertainty intervals reported by the GBD study.

### 2.10. GBD World Standard Population

Age-standardized rates used the GBD world standard population, based on unweighted averages of national age distributions from UN 2012 Revision prospects (2010–2035), continued in GBD 2023 with internal population estimates.

### 2.11. Funding, Ethics, and Data Sharing

This research received no specific funding from public, commercial, or not-for-profit sectors. Full data access was available to authors, who take final responsibility for submission. As secondary public data were used, no institutional ethics approval was required. All data are publicly available via GBD 2023 on the Global Health Data Exchange (http://ghdx.healthdata.org).

## 3. Results

### 3.1. Overall Patterns Across Genitourinary Cancers

In 2023, genitourinary cancers exhibited pronounced heterogeneity across the Americas, strongly patterned by sociodemographic level. A consistent gradient emerged, whereby high-SDI settings showed higher age-standardized incidence rates, while mortality and DALY rates were disproportionately concentrated in Latin America and the Caribbean and lower-SDI regions (Table 1). Notably, this divergence between incidence and outcomes was observed across all four cancer types, a pattern that may reflect differences in detection capacity and survival outcomes across settings. Supporting data are provided in Supplementary Tables S2–S13. Additional interval-specific analyses demonstrated substantial temporal heterogeneity across calendar periods, particularly for prostate cancer and in lower-SDI settings. Detailed APC estimates and corresponding 95% confidence intervals are presented in Supplementary Table S17 and Supplementary Figures S1–S4.

This pattern was further supported by MIR analyses (Supplementary Table S16). Across all four malignancies, MIRs increased progressively as SDI decreased. High-income North America consistently exhibited the lowest MIR values, whereas low-SDI regions showed the highest ratios. For example, prostate cancer MIR increased from 0.169 in high-income North America to 0.816 in low-SDI settings, while bladder cancer MIR increased from 0.248 to 0.607. This inverse relationship between incidence and mortality is consistent with differences in cancer detection, management, and healthcare resources across regions.

### 3.2. Prostate Cancer

Prostate cancer represented the largest burden among genitourinary malignancies in 2023, with 1.41 million incident cases, 472,953 deaths, and 8.9 million DALYs (Table 1). A striking geographic mismatch emerged: while incidence peaked in high-income North America, mortality and DALY rates were highest in Latin America and the Caribbean.

Across SDI levels, contrasting patterns became evident. High-SDI settings combined elevated incidence with more favorable mortality and DALY profiles, whereas lower-SDI regions showed poorer outcomes despite lower incidence (Table 1). This inverse relationship between incidence and mortality is consistent with differences in cancer detection, management, and healthcare resources across regions, although these factors could not be directly evaluated in the present ecological analysis.

MIR analysis further highlighted these disparities. The prostate cancer MIR increased from 0.169 in high-income North America to 0.816 in low-SDI regions and reached 0.420 in Latin America and the Caribbean. These findings suggest substantial inequalities in survival outcomes despite lower incidence rates in less developed settings.

**Figure 1:**
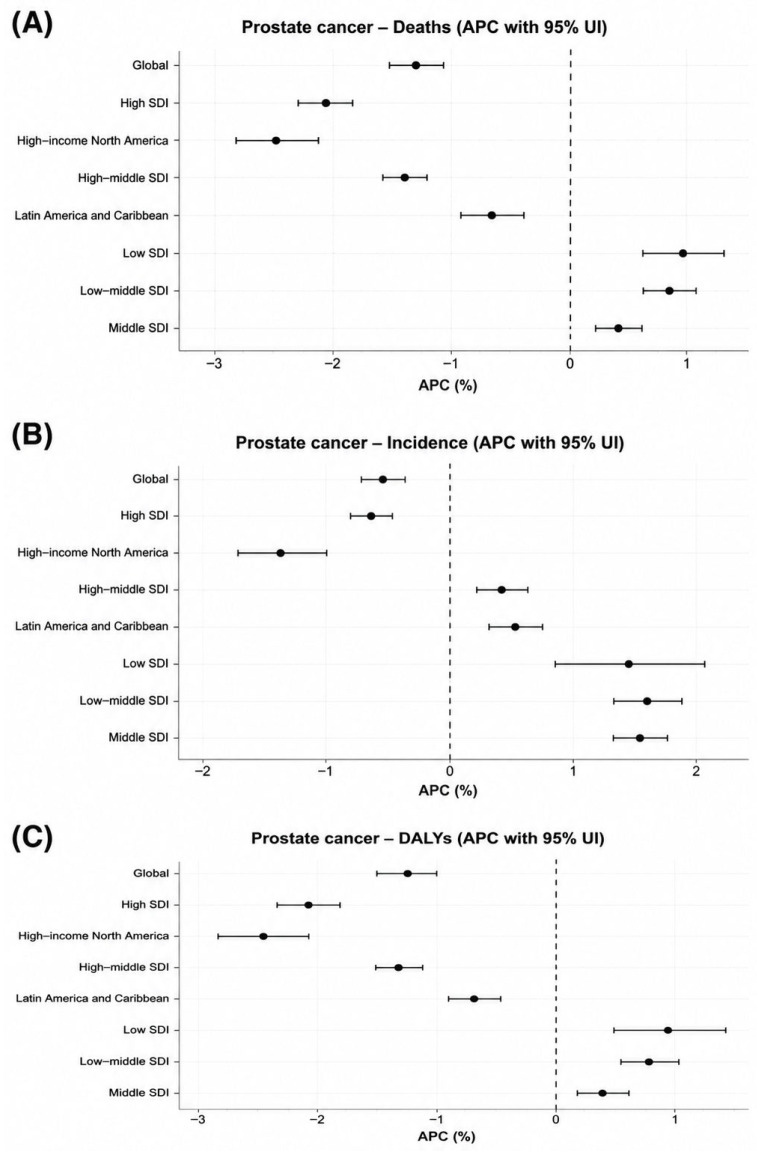
Temporal trends in age-standardized prostate cancer burden across global, regional, and Sociodemographic Index (SDI) groups, 2000–2023. (**A**) Annual percentage change (APC) in age-standardized mortality rates (ASMR). (**B**) APC in age-standardized incidence rates (ASIR). (**C**) APC in age-standardized disability-adjusted life year (DALY) rates. Points represent APC estimates derived from weighted log-linear regression models, and horizontal bars indicate 95% confidence intervals. The vertical dashed line indicates no temporal change (APC = 0). Negative APC values indicate decreasing trends over time, whereas positive APC values indicate increasing trends. SDI categories represent levels of socioeconomic development based on income, education, and fertility. High-income North America and high-SDI regions generally exhibited declining mortality and DALY trends despite relatively high incidence rates, whereas low-, low-middle-, and middle-SDI regions showed increasing incidence and less favorable mortality and DALY trajectories, highlighting persistent socioeconomic gradients in prostate cancer burden across the Americas.

Age-standardized rates enabled more meaningful comparisons of disease burden across populations with different demographic structures. Globally, the age-standardized incidence rate (ASIR) was 33.68 (95% UI: 28.56–38.69) per 100,000 population, with the highest rate observed in high-income North America at 96.75 (77.86–116.23), followed by Latin America and the Caribbean at 54.53 (44.25–65.68), and the lowest in low-middle SDI regions at 16.98 (12.58–21.47). The global age-standardized mortality rate (ASMR) was 12.48 (10.88–13.95), reaching its highest value in Latin America and the Caribbean at 22.88 (20.65–24.51). The global age-standardized DALY rate was 218.60 (192.71–244.97), with the greatest burden also observed in Latin America and the Caribbean at 395.92 (365.48–421.98) (Table 1). Temporal trends further reinforced these disparities. From 2000 to 2023, high-income North America experienced sustained declines in incidence, mortality, and DALYs. In contrast, lower-SDI regions showed increasing trends across all three indicators, indicating a growing burden rather than epidemiological transition toward control (Table 2; Figure 1). Detailed data are provided in Supplementary Tables S2, S6, and S10.

Interval-specific analyses revealed that declines in incidence, mortality, and DALY rates were most pronounced in high-income North America and high-SDI settings during the early and middle study periods, whereas low-SDI regions experienced persistent increases across successive calendar intervals (Supplementary Table S17; Supplementary Figure S1).

High-SDI regions and High-income North America generally experienced declining mortality and DALY rates for most genitourinary cancers. In contrast, low-, low-middle-, and middle-SDI regions showed increasing incidence trends and less favorable mortality trajectories, particularly for prostate, kidney, and testicular cancers. Latin America and the Caribbean demonstrated increasing incidence rates across all four cancers, with particularly marked increases in testicular and kidney cancer burden, highlighting persistent socioeconomic gradients in genitourinary cancer outcomes across the Americas.

Substantial heterogeneity was also observed at the national level in 2023. The age-standardized incidence rate (ASIR) ranged from 170.63 (95% UI: 126.99–226.49) per 100,000 population in Bermuda and 140.02 (96.31–187.65) in Dominica to 29.17 (16.97–42.96) in Greenland. The highest age-standardized mortality rates (ASMRs) were recorded in Dominica (87.02 [61.77–117.09]) and Trinidad and Tobago (81.84 [68.81–94.43]), whereas the lowest were observed in Puerto Rico (15.57 [13.58–17.34]) and Mexico (15.65 [14.37–16.71]). Similarly, Dominica exhibited the greatest DALY burden, with an age-standardized rate of 1423.30 (1027.70–1909.51) per 100,000 population.

Estimates were obtained from the Global Burden of Disease (GBD) 2023 study. Age-standardized rates were calculated using the GBD world standard population to facilitate comparisons across regions and Sociodemographic Index (SDI) categories. High-income North America consistently exhibited the highest incidence rates for most genitourinary cancers, whereas Latin America and the Caribbean and lower-SDI regions showed disproportionately higher mortality and disability-adjusted life year (DALY) burdens relative to incidence, highlighting persistent socioeconomic disparities in cancer outcomes and access to healthcare across the Americas. These findings should also be interpreted in the context of contemporary diagnostic practices. Higher prostate cancer incidence in high-SDI settings may partly reflect greater use of prostate-specific antigen (PSA) testing and multiparametric magnetic resonance imaging (MRI), which increase detection of indolent tumors. Current clinical guidelines increasingly emphasize risk-adapted diagnostic pathways and active surveillance for selected low-risk disease to reduce overdiagnosis and overtreatment.

### 3.3. Testicular Cancer

In 2023, testicular cancer accounted for 100,215 incident cases and 11,909 deaths globally (Table 1). Despite its relatively low overall burden, a notable paradox emerged: incidence increased across all SDI levels, yet mortality declines were largely restricted to high-SDI settings.

**Figure 2:**
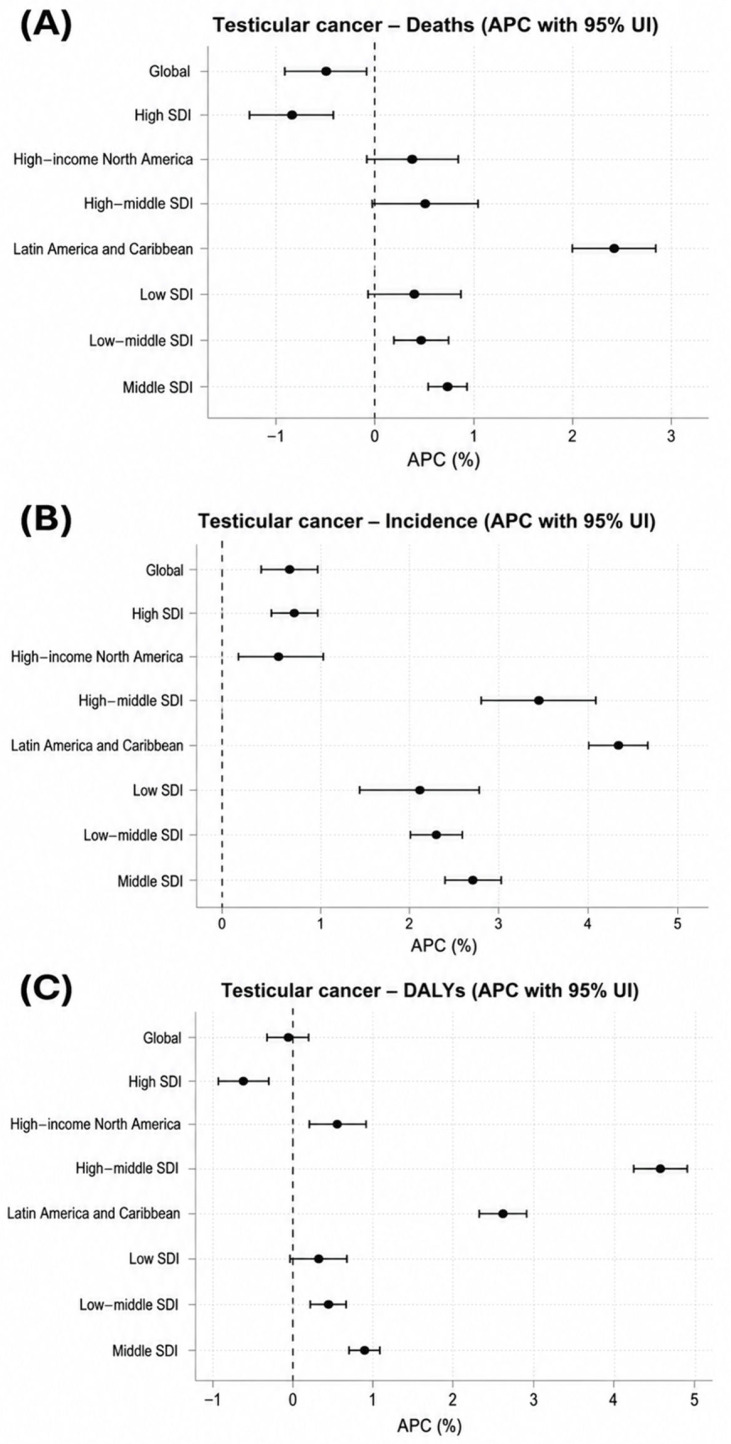
Temporal trends in age-standardized testicular cancer burden across global, regional, and Sociodemographic Index (SDI) groups, 2000–2023. (**A**) Annual percentage change (APC) in age-standardized mortality rates (ASMR). (**B**) APC in age-standardized incidence rates (ASIR). (**C**) APC in age-standardized disability-adjusted life year (DALY) rates. Points represent APC estimates and horizontal bars represent 95% confidence intervals. The vertical dashed line indicates no temporal change (APC = 0). Positive APC values indicate increasing trends, whereas negative values indicate decreasing trends. Although testicular cancer is generally associated with favorable survival outcomes, incidence increased across nearly all SDI groups and regions.

Contrary to expectations for a highly curable malignancy, mortality and DALYs increased in Latin America and the Caribbean (Table 2; Figure 2). This pattern is consistent with unequal improvements in cancer outcomes across regions and may reflect differences in healthcare access, early diagnosis, treatment availability, or other unmeasured factors. The MIR for testicular cancer was nearly eight-fold higher in low-SDI regions (0.316) than in high-income North America (0.041) and is consistent with potential differences in treatment access and healthcare quality for this otherwise highly curable malignancy.

The largest increases were observed in Latin America and the Caribbean and in high-middle SDI settings. Mortality and DALY reductions were mainly observed in high-SDI regions, whereas several lower-SDI settings exhibited stable or increasing mortality trends, suggesting unequal improvements in outcomes across the Americas. Across SDI strata, a divergence between rising incidence and uneven mortality trends became evident, particularly in middle and high-middle SDI regions, where mortality did not decline proportionally. Country-level estimates revealed additional heterogeneity, with several Latin American countries showing disproportionately high mortality relative to incidence (Supplementary Table S7).

Taken together, these findings suggest that factors beyond disease occurrence may contribute to the higher burden observed in lower-resource settings; however, the relative contributions of diagnosis, treatment, healthcare access, and registry characteristics could not be assessed in the present ecological analysis. Globally, the age-standardized incidence and mortality rates were 2.4 (95% UI: 1.7–3.1) and 0.28 (0.23–0.35) per 100,000 population, respectively. High-income North America exhibited the highest incidence rate at 7.3 (5.1–9.7), followed by Latin America and the Caribbean at 5.2 (3.9–6.7), whereas the lowest incidence was observed in low-SDI regions at 0.79 (0.43–1.3). The lowest mortality rate was recorded in low-middle SDI regions at 0.24 (0.15–0.35) per 100,000 population. At the national level, substantial variation was observed, with Bermuda reporting the highest prostate cancer ASIR (170.63 per 100,000 population) and Dominica the highest DALY rate (1423.30 per 100,000 population).

Interval-specific APC analyses showed sustained increases in incidence across most regions and SDI groups, with particularly marked increases in Latin America and the Caribbean and high-middle SDI settings during recent calendar periods (Supplementary Table S17; Supplementary Figure S2).

Testicular cancer provides an important example of potentially avoidable mortality disparities, as survival approaches 100% for many patients with stage I disease when surveillance protocols, serum tumor markers, imaging, and effective salvage therapies are available. Consequently, persistent mortality differences across regions may be consistent with inequalities in access to specialized oncologic care.

### 3.4. Kidney and Bladder Cancers (Both Sexes)

Bladder and kidney cancers displayed broadly similar epidemiological patterns across regions and SDI levels. In 2023, bladder cancer accounted for 572,796 incident cases and 233,666 deaths, while kidney cancer contributed to 400,948 cases and 165,265 deaths (Table 1).

Globally, the age-standardized incidence rate (ASIR) was 6.2 (95% UI: 5.4–7.1) per 100,000 population for bladder cancer and 4.4 (3.8–5.2) for kidney cancer, while the corresponding age-standardized mortality rates (ASMRs) were 2.6 (2.3–2.8) and 1.8 (1.6–1.9), respectively. High-income North America consistently exhibited the highest burden, with bladder cancer ASIR and ASMR of 13.5 (11.5–15.2) and 3.3 (3.0–3.6), respectively, and kidney cancer ASIR and ASMR of 13.5 and 3.3 per 100,000 population. In contrast, low-middle SDI regions had the lowest bladder cancer incidence, with an ASIR of 2.3 (1.7–3.1).

A consistent but uneven gradient was observed: incidence rates were highest in high-income North America and high-SDI settings, whereas mortality remained disproportionately elevated in lower-SDI regions. Notably, Latin America and the Caribbean showed persistently higher mortality relative to incidence, reflecting less favorable survival outcomes compared with high-income regions.

Similar patterns were observed for kidney and bladder cancers. Kidney cancer MIR increased from 0.276 in high-income North America to 0.615 in low-SDI settings, while bladder cancer MIR increased from 0.248 to 0.607. Latin America and the Caribbean also showed elevated MIRs for both cancers compared with high-income North America, suggesting persistent regional inequalities in cancer management and survival.

**Figure 3:**
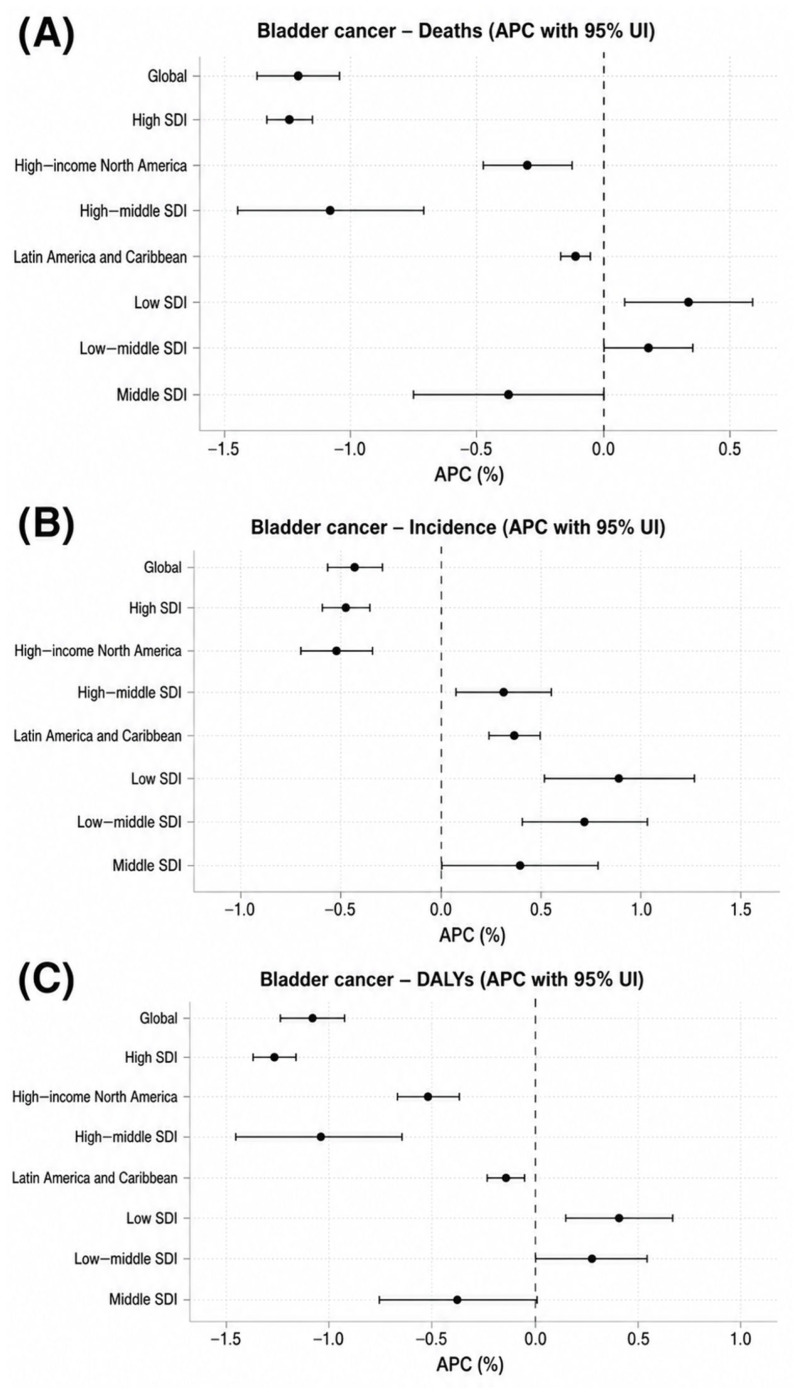
Temporal trends in age-standardized kidney cancer burden across global, regional, and Sociodemographic Index (SDI) groups, 2000–2023. (**A**) Annual percentage change (APC) in age-standardized mortality rates (ASMR). (**B**) APC in age-standardized incidence rates (ASIR). (**C**) APC in age-standardized disability-adjusted life year (DALY) rates. Points represent APC estimates obtained from weighted log-linear regression analyses and horizontal bars indicate 95% confidence intervals. The vertical dashed line indicates no temporal change (APC = 0). Negative APC values indicate declining trends, whereas positive values indicate increasing trends over time. High-income North America and high-SDI regions generally showed declining incidence, mortality, and DALY rates. In contrast, Latin America and the Caribbean and lower-SDI regions exhibited increasing incidence, mortality, and DALY trends, indicating a growing burden of kidney cancer and persistent socioeconomic inequalities across the Americas.

**Figure 4:**
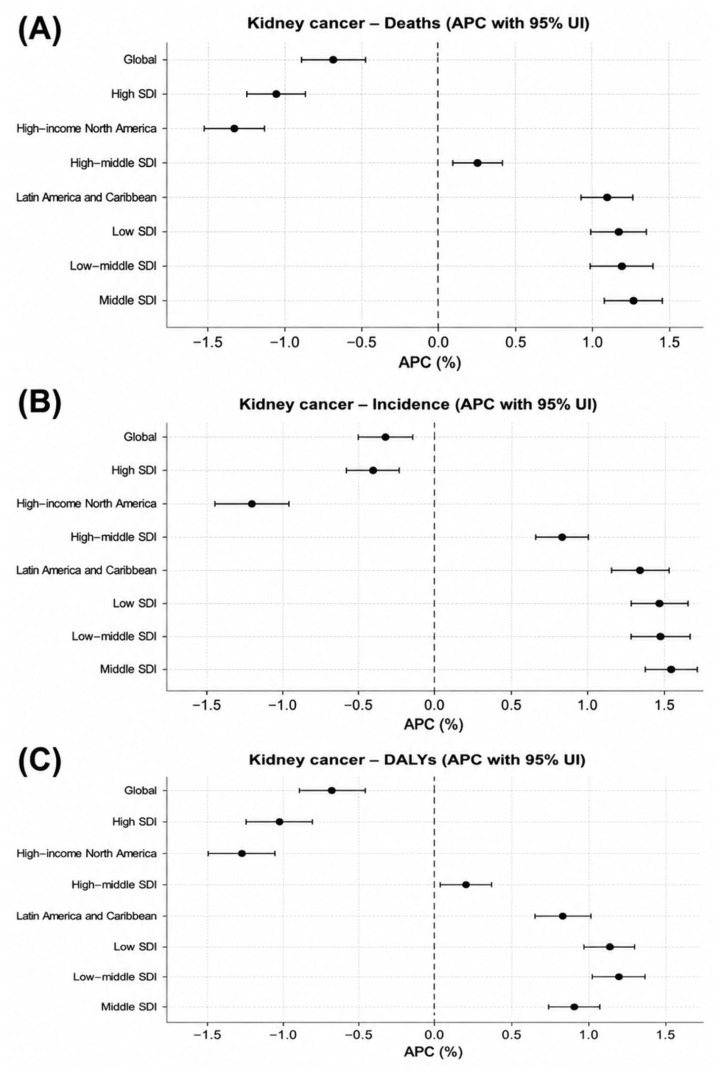
Temporal trends in age-standardized bladder cancer burden across global, regional, and Sociodemographic Index (SDI) groups, 2000–2023. (**A**) Annual percentage change (APC) in age-standardized mortality rates (ASMR). (**B**) APC in age-standardized incidence rates (ASIR). (**C**) APC in age-standardized disability-adjusted life year (DALY) rates. Points represent APC estimates and horizontal bars indicate 95% confidence intervals. The vertical dashed line indicates no temporal change (APC = 0). Positive APC values indicate increasing trends, whereas negative values indicate decreasing trends over time. Most high-SDI regions demonstrated declining incidence, mortality, and DALY rates, whereas low-SDI and low-middle SDI settings experienced stable or increasing trends. Latin America and the Caribbean showed modest increases in incidence accompanied by limited reductions in mortality and DALYs, reflecting persistent disparities in bladder cancer outcomes across the Americas.

Temporal trends revealed further divergence. A striking pattern emerged, with declining incidence and mortality in high-SDI settings—particularly for bladder cancer—contrasted by increasing incidence and slower or absent mortality reductions in low- and middle-SDI regions (Table 2; Figure 3 and Figure 4). Kidney cancer followed a similar trajectory, with declining trends in high-SDI settings but rising incidence in lower-SDI groups.

Interval-specific analyses confirmed considerable temporal heterogeneity. For kidney cancer, incidence increases were particularly evident in Latin America and the Caribbean and lower-SDI settings during recent periods, whereas bladder cancer showed declining trends in many high-SDI regions but less favorable trajectories in lower-SDI groups (Supplementary Table S1; Supplementary Figures S3 and S4).

High-SDI regions and high-income North America showed declining mortality and DALY trends despite stable or decreasing incidence rates, suggesting improvements in early detection, diagnostic imaging, and access to effective treatment. In contrast, low-, low-middle-, and middle-SDI regions experienced increasing incidence, mortality, and DALY rates, particularly in Latin America and the Caribbean, indicating a growing burden of kidney and bladder cancers and persistent disparities in healthcare access and oncologic care across the Americas. In 2023, the highest kidney cancer burden was observed in Uruguay, with an ASIR of 13.17 (95% UI: 11.37–15.06) and an ASMR of 6.50 (5.86–7.12) per 100,000 population. For bladder cancer, the highest ASIR was reported in the United States at 14.18 (12.06–15.96), while the highest ASMR was observed in Greenland at 5.99 (4.44–7.73) per 100,000 population (Tables S7 and S8).

At the national level, heterogeneity became particularly evident. Countries such as Uruguay and the United States showed the highest incidence rates, whereas smaller populations, including Greenland, exhibited disproportionately high mortality rates. This mismatch between incidence and mortality highlights potential inequities in access to diagnosis and treatment across countries. Detailed results are presented in Supplementary Tables S4–S9, S12 and S13.

For kidney cancer, increasing incidence trends may partially reflect greater use of abdominal imaging and incidental tumor detection. Established risk factors include smoking, obesity, and hypertension, although these variables were not directly evaluated in the present study [34,35]. Routine population-based screening for renal cell carcinoma is not currently recommended. In bladder cancer, differences between non-muscle-invasive and muscle-invasive disease may contribute to outcome variability. Tobacco use, occupational carcinogen exposure, timely evaluation of hematuria, accurate staging, and access to definitive treatment are recognized determinants of prognosis and may contribute to some of the regional differences observed across the Americas [36].

### 3.5. Summary of Disparities

Across all four cancer types, a coherent and consistent pattern emerged: higher incidence in more developed settings was accompanied by more favorable mortality and DALY outcomes, whereas lower-SDI regions experienced worse outcomes despite lower or moderate incidence (Table 1 and Table 2).

Importantly, these disparities were not static but persisted—and in some cases widened—over time, as evidenced by temporal trend analyses (Figure 1, Figure 2, Figure 3 and Figure 4). This suggests that improvements in cancer control have been unevenly distributed across the Americas, reinforcing existing socioeconomic gradients in health outcomes.

## 4. Discussion

The present study provides a comprehensive assessment of genitourinary cancer burden across the Americas using GBD 2023 estimates, revealing persistent and, in some cases, widening socioeconomic disparities in incidence, mortality, and DALYs. A consistent and structurally relevant pattern emerged: higher-SDI regions exhibited elevated incidence alongside more favorable mortality and DALY outcomes, whereas lower-SDI regions experienced less favorable outcomes despite lower or moderate incidence [37]. This divergence highlights that differences in survival are not primarily driven by disease occurrence, but rather by inequities in healthcare access, early detection, and treatment capacity.

Prostate cancer exemplifies this imbalance [4]. While incidence was highest in high-income North America, mortality and DALYs remained disproportionately elevated in Latin America and the Caribbean. This geographic decoupling between incidence and mortality suggests that detection alone is insufficient to reduce disease burden in the absence of effective treatment pathways. In high-SDI settings, declining mortality likely reflects improvements in early detection, risk stratification, and access to definitive therapies. In contrast, in lower-SDI regions, later-stage diagnosis and constrained access to specialized care continue to limit survival gains [2,38]. Differences in prostate-specific antigen (PSA) [39] testing practices further contribute to divergent incidence trends, with more structured or intensive screening in high-income settings and more heterogeneous or opportunistic approaches elsewhere [40].

Interpretation of prostate cancer incidence patterns requires consideration of contemporary diagnostic practices. Higher incidence rates in high-SDI settings may partly reflect greater use of prostate-specific antigen (PSA) testing, multiparametric magnetic resonance imaging (mpMRI), and increased detection of low-risk disease. Current European guidelines advocate risk-adapted diagnostic pathways incorporating PSA assessment, MRI-based triage, and selective biopsy strategies, while active surveillance is recommended for many patients with low-risk tumors to reduce overtreatment [41,42]. Therefore, higher incidence in more developed settings should not necessarily be interpreted as a greater underlying disease burden.

Testicular cancer revealed a particularly important and counterintuitive pattern. Despite being one of the most curable solid malignancies, mortality and DALY trends remained unfavorable in several lower-SDI settings. Contrary to expectations, survival gains were largely confined to high-SDI regions, while Latin America and the Caribbean showed increases in both incidence and mortality. This finding underscores that the prognosis of testicular cancer is highly dependent on timely diagnosis and access to standardized treatment protocols, including tumor marker assessment and chemotherapy. The persistence of elevated mortality in lower-resource settings may be consistent with differences in healthcare delivery, access to specialized care, follow-up systems, and treatment implementation rather than limitations in the effectiveness of currently available therapies [43]. The unfavorable mortality patterns observed for testicular cancer in several countries of Latin America and the Caribbean contrast with trends reported in Europe. Bray et al. documented continued increases in testicular cancer incidence accompanied by sustained declines in mortality across 22 European countries, suggesting that improvements in early diagnosis, access to cisplatin-based therapies, and specialized oncologic care have translated into substantial survival gains. In contrast, our findings indicate that mortality reductions in the Americas have been less uniform, particularly in lower-SDI settings, highlighting persistent inequalities in access to cancer care and treatment outcomes [44].

Testicular cancer deserves particular attention because survival rates should approach 100% for many patients diagnosed with stage I disease when contemporary management is available [45,46,47]. Current clinical practice relies on risk-adapted surveillance protocols, serum tumor marker assessment, cro ss-sectional imaging, and highly effective cisplatin-based salvage therapies [45,46]. Therefore, the persistence of unfavorable mortality trends in some lower-SDI settings is clinically meaningful and may be consistent with differences in access to specialized multidisciplinary care, follow-up programs, and treatment delivery.

Bladder and kidney cancers further reinforced the presence of asymmetric epidemiological transitions across the region. For bladder cancer, important clinical distinctions exist between non-muscle-invasive bladder cancer (NMIBC) and muscle-invasive bladder cancer (MIBC), which differ substantially in prognosis and management [48,49]. Population-level disparities may be influenced by differences in tobacco exposure, occupational carcinogen exposure, timely evaluation of hematuria, access to cystoscopy and pathology services, accurate staging, and availability of intravesical and definitive treatments [50,51]. In high-SDI settings, stable or declining mortality alongside relatively high incidence suggests improvements in early detection and clinical management. In contrast, in lower-SDI regions, increasing incidence without proportional declines in mortality indicates a growing mismatch between disease occurrence and survival gains [52]. This divergence likely reflects combined effects of differential exposure to risk factors, limited diagnostic capacity, and restricted access to effective treatment.

For kidney cancer, established risk factors include tobacco use, obesity, hypertension, and metabolic disorders. Furthermore, increasing incidence in high-SDI settings may partly reflect the widespread use of abdominal imaging, which facilitates the incidental detection of asymptomatic renal masses [34,53]. Importantly, current clinical guidelines do not recommend population-based screening for renal cell carcinoma because available evidence does not demonstrate a favorable balance between benefits and harm, leading to the detection of incidental and potentially indolent tumors [54]. However, the simultaneous decline in mortality suggests that improvements in clinical management have offset the potential harms of overdiagnosis. In lower-SDI regions, where diagnostic and treatment infrastructure is more limited, rising incidence is more likely to translate into increased mortality and DALY burden, reinforcing existing inequalities [54]. Taken together, these findings highlight that the burden of genitourinary cancers in the Americas is shaped less by biological differences and more by structural determinants of health. Access to timely diagnosis, availability of specialized oncologic care, and health system capacity appear to be the primary drivers of the observed disparities. Importantly, the consistent pattern across all four cancer types suggests that these inequities are systemic rather than disease-specific.

From a public health perspective, these results underscore the need for context-specific and resource-sensitive cancer control strategies. In high-SDI settings, priorities may include optimizing diagnostic pathways to minimize overdiagnosis and overtreatment while maintaining high survival rates. In contrast, in lower-SDI regions, the primary challenge remains strengthening health system capacity, particularly by improving access to early diagnosis, ensuring availability of essential medicines, and expanding access to evidence-based treatment [5]. Given the high curability of testicular cancer and the relatively favorable prognosis of many prostate cancers when detected early, substantial reductions in mortality could be achieved through targeted improvements in care delivery.

Future projections indicate that cancer burden in low- and middle-income countries will continue to rise due to population growth, ageing, and increasing exposure to risk factors associated with urbanization and industrialization. While high-income countries have achieved meaningful reductions in mortality, these gains have not been equitably distributed. Closing this gap will require coordinated efforts to integrate effective diagnostic strategies, strengthen oncology services, and ensure equitable access to high-quality care across the region [5].

Beyond regional and SDI-level patterns, substantial heterogeneity was observed across individual countries in the Americas. Bermuda and Dominica consistently exhibited some of the highest prostate cancer incidence rates, whereas Dominica and Trinidad and Tobago showed the highest mortality and DALY burdens. For kidney cancer, Uruguay recorded the highest incidence and mortality rates, while the United States exhibited the highest bladder cancer incidence and Greenland the highest bladder cancer mortality. Similarly, testicular cancer incidence was concentrated in high-income settings, whereas mortality and DALY burdens remained disproportionately elevated in several Latin American and Caribbean countries. These findings suggest that disparities in genitourinary cancer burden are influenced not only by broad socioeconomic gradients but also by country-specific differences in healthcare infrastructure, access to early diagnosis, treatment availability, cancer control policies, and population risk-factor profiles. Detailed country-level estimates for all 38 countries and territories are presented in the Supplementary Materials (Tables S2–S15).

Comparisons with Europe provide additional context for interpreting the disparities observed across the Americas. Similar to our findings, several European countries report higher incidence rates of prostate, kidney, and bladder cancers in more developed settings, partly attributable to greater diagnostic intensity, population aging, and wider access to screening and imaging technologies [2,13,55]. However, Europe generally exhibits lower mortality-to-incidence ratios and more favorable survival outcomes, reflecting stronger healthcare infrastructure, earlier diagnosis, and broader access to specialized oncologic care [37,56]. These differences suggest that socioeconomic development alone does not fully explain variations in cancer outcomes; rather, the organization, accessibility, and quality of healthcare systems appear to play a fundamental role in reducing mortality and improving survival among patients with genitourinary malignancies [56,57].

This study has several strengths. By leveraging GBD 2023 estimates, we provide standardized and comparable data across 38 countries and territories, allowing for robust assessment of temporal trends and socioeconomic gradients from 1990 to 2023. The integration of incidence, mortality, and DALYs offers a multidimensional perspective on cancer burden, while the use of a fitted log-linear model allows for a detailed characterization of temporal changes. Adherence to GATHER guidelines further supports transparency and reproducibility.

However, several limitations should be considered. First, the ecological design precludes causal inference and is inherently susceptible to ecological fallacy. Therefore, associations observed at the population level should not be interpreted as reflecting individual-level risk relationships, and the findings should be regarded as correlational rather than causal. Second, country-level estimates may mask important subnational heterogeneity, particularly in large and socioeconomically diverse countries. Third, the GBD 2023 database does not provide information on tumor stage at diagnosis, histological grade, molecular characteristics, screening practices, treatment patterns, or healthcare utilization. Because GBD estimates are derived from population-level statistical modeling frameworks rather than individual patient records, stage-specific and grade-specific distributions cannot be obtained from the available dataset. Consequently, the present study was unable to evaluate regional differences according to disease stage or grade, which may contribute to the mortality disparities observed across the Americas. Fourth, GBD estimates are partially model-generated through frameworks such as CODEm and DisMod-MR 2.1. Although these approaches improve comparability across countries and help address data gaps, estimates for locations with sparse cancer registry coverage or incomplete mortality reporting may rely more heavily on statistical modeling and therefore remain subject to greater uncertainty. Consequently, findings from countries with limited underlying data should be interpreted with appropriate caution. Fifth, although GBD 2023 incorporates recent disruptions such as the COVID-19 pandemic, the specific impact of these factors on cancer detection, diagnosis, treatment, and outcomes could not be disentangled in the present analysis. Sixth, the present study focused primarily on age-standardized estimates to facilitate comparisons across countries, regions, and SDI groups. Consequently, age-specific patterns were not evaluated. In addition, kidney and bladder cancers were analyzed using sex-combined estimates, which may have obscured important biological, behavioral, and healthcare-related differences between males and females. Finally, although mortality-to-incidence ratios (MIRs) were calculated for global, regional, and SDI groupings, country-level MIRs were not separately presented because country-specific age-standardized incidence and mortality rates are already available in the Supplementary Materials. Presenting both measures would have resulted in substantial duplication of information. Nevertheless, the reported ASIR and ASMR values allow independent calculation and verification of MIRs for individual countries.

Despite these limitations, the present study provides one of the most comprehensive assessments of genitourinary cancer burden across the Americas using the most recent GBD 2023 estimates. An additional strength of this analysis is the formal evaluation of mortality-to-incidence ratios. Across all four genitourinary malignancies, MIRs exhibited a clear inverse relationship with socioeconomic development. High-SDI regions and high-income North America consistently demonstrated lower MIRs despite higher incidence rates, whereas low-SDI regions and Latin America and the Caribbean exhibited substantially higher MIRs [20,37]. Because MIR is widely regarded as a population-level surrogate indicator of cancer survival and healthcare system effectiveness, these findings support the interpretation that inequalities in access to screening, timely diagnosis, specialized treatment, and long-term follow-up remain important determinants of genitourinary cancer outcomes throughout the Americas [37,58].

Finally, some estimates were associated with relatively wide uncertainty intervals, particularly in locations with limited data availability. Consequently, small differences between countries, regions, or SDI categories should be interpreted with caution, especially when uncertainty intervals overlap.

## 5. Conclusions

Genitourinary cancer burden in the Americas shows persistent socioeconomic disparities. High-SDI regions benefit from improved detection and treatment, whereas lower-SDI settings face rising incidence without equivalent survival gains. Strengthening timely diagnosis and access to evidence-based care is essential to reduce mortality and DALYs. Given the high curability of testicular cancer and the manageable course of many prostate cancers, targeted improvements in healthcare delivery could yield rapid and cost-effective reductions in disease burden across the region.

## Figures and Tables

**Table 1:** Global, region and SDI. Cancer incidence, death, and DALY count and age-standardized rates.

Cancer Type	Location	Incident Cases, 2023, in Thousands (95% UI)	Age-Standardized Incidence Rate/100,000, 2023 (95% UI)	Deaths, 2023, in Thousands (95% UI)	Age-Standardized Death Rate/100,000, 2023 (95% UI)	DALYs, 2023, in Thousands (95% UI)	Age-Standardized DALY Rate/100,000, 2023 (95% UI)
Prostate cancer, male	Global	1417 (1196–1634)	33.6 (28.5–38.6)	472 (415–530)	12.4 (10.8–13.9)	8907 (7865–10,012)	218.5 (192.6–244.9)
	High-income North America	312 (251–337)	96.7 (77.8–116.2)	51 (44–57)	16.3 (13.9–18.5)	1025 (892–1147)	320.6 (279.2–358.9)
	Latin America and the Caribbean	159 (129–192)	54.5 (44.2–65.6)	61 (56–65)	22.8 (20.6–24.5)	1127 (1042–1201)	395.9 (365.4–421.9)
	High SDI	1021 (871–1184)	49.3 (42.0–57.1)	268 (238–289)	13.6 (12.0–14.7)	4858 (4393–5237)	239.2 (215.8–258.0)
	High-middle SDI	186 (146–232)	19.6 (15.5–24.1)	76 (65–88)	8.9 (7.7–10.4)	1447 (1253–1690)	156.8 (136.2–183.4)
	Middle SDI	63 (46–80)	16.9 (12.5–21.4)	32 (24–40)	10.2 (7.5–12.8)	656 (489–826)	180.0 (133.4–226.3)
	Low-middle SDI	63 (47–82)	15.7 (11.8–20.1)	37 (27–47)	10.4 (7.8–13.5)	735 (547–956)	183.9 (137.2–237.9)
	Low SDI	79 (54–110)	20.1 (13.8–28.1)	58 (39–82)	16.4 (11.0–23.3)	1194 (829–1713)	298.0 (205.4–424.6)
Testicular cancer, male	Global	100 (74.5–129.5)	2.4 (1.7–3.1)	11 (9.53–14.6)	0.28 (0.23–0.35)	587 (470–730)	14.2 (11.3–17.6)
	High-income North America	13 (9–18)	7.3 (5.1–9.7)	6 (5–8)	0.30 (0.25–0.37)	35 (28–44)	18.1 (14.5–22.9)
	Latin America and the Caribbean	15 (12–20)	5.2 (3.9–6.7)	2 (2–2)	0.71 (0.65–0.78)	115 (104–127)	38.0 (34.3–42.1)
	High SDI	62 (45–79)	4.6 (3.3–5.8)	4 (4–5)	0.28 (0.26–0.30)	207 (185–233)	14.9 (13.4–16.9)
	High-middle SDI	19 (14–26)	2.3 (1.7–3.0)	3 (2–3)	0.34 (0.29–0.40)	147 (125–174)	17.2 (14.6–20.3)
	Middle SDI	6 (4–9)	1.1 (0.7–1.7)	1 (0.8–1.0)	0.25 (0.17–0.34)	57 (37–79)	11.5 (7.5–15.7)
	Low-middle SDI	5 (3–8)	0.87 (0.5–1.3)	1 (0.8–2.0)	0.24 (0.15–0.35)	73 (60–85)	11.9 (7.4–17.5)
	Low SDI	6 (3–10)	0.79 (0.43–1.3)	2 (1–3)	0.25 (0.15–0.39)	102 (58–157)	12.9 (7.4–19.9)
Kidney cancer, both sexes	Global	400 (347–465)	4.4 (3.8–5.2)	165 (146–179)	1.8 (1.6–1.9)	4061 (3600–4509)	45.5 (40.2–50.5)
	High-income North America	65 (56–75)	10.6 (9.07–12.2)	20 (18–21)	2.9 (2.6–3.1)	440 (404–477)	71.2 (65.4–77.6)
	Latin America and the Caribbean	31 (27–35)	4.7 (4.2–5.4)	14 (13–15)	2.19 (2.05–2.31)	396 (372–416)	60.81 (57.25–64.07)
	High SDI	294 (254–332)	7.2 (6.2–8.1)	115 (104–123)	2.5 (2.3–2.7)	2544 (2335–2690)	62.1 (57.3–65.7)
	High-middle SDI	56 (46–74)	2.9 (2.4–3.9)	24 (20–30)	1.20 (1.0–1.4)	664 (570–826)	34.08 (29.2–42.4)
	Middle SDI	19 (15–24)	2.0 (1.6–2.6)	8 (6–10)	0.9 (0.7–1.1)	269 (205–334)	28.72 (22.03–35.34)
	Low-middle SDI	14 (10–18)	1.4 (1.0–1.8)	7 (6–9)	0.8 (0.62–1.0)	246 (185–310)	24.02 (18.09–30.23)
	Low SDI	16 (11–21)	1.3 (0.95–1.7)	9 (6–11)	0.8 (0.61–1.12)	331 (231–444)	25.92 (18.09–34.02)
Bladder cancer, both sexes	Global	572 (501–649)	6.2 (5.4–7.1)	233 (208–257)	2.6 (2.3–2.8)	4625 (4221–5130)	50.7 (46.2–56.3)
	High-income North America	93 (79–105)	13.5 (11.5–15.2)	24 (22–26)	3.3 (3.0–3.6)	452 (410–488)	65.5 (59.8–70.7)
	Latin America and the Caribbean	24 (21–27)	3.76 (3.2–4.2)	12 (11–13)	1.9 (1.7–2.0)	251 (234–264)	38.32 (35.7–40.3)
	High SDI	412 (359–462)	9.0 (7.8–10.1)	156 (141–166)	3.2 (2.9–3.4)	2851 (2649–3012)	62.8 (58.7–66.4)
	High-middle SDI	76 (65–89)	3.7 (3.2–4.4)	34 (30–39)	1.7 (1.5–1.9)	733 (658–826)	36.0 (32.4–40.6)
	Middle SDI	32 (25–40)	3.7 (2.2–4.6)	14 (12–17)	1.7 (1.4–2.2)	348 (286–429)	39.33 (32.60–48.35)
	Low-middle SDI	22 (15–29)	2.3 (1.7–3.1)	12 (8–16)	1.4 (1.0–1.8)	279 (201–374)	29.88 (21.51–40.13)
	Low SDI	28 (20–39)	3.13 (2.2–4.3)	16 (11–23)	1.9 (1.3–2.8)	406 (287–572)	43.27 (30.47–61.14)

Global, regional, and Sociodemographic Index (SDI)-specific burden of prostate, testicular, kidney, and bladder cancers in 2023. Incident cases, deaths, and disability-adjusted life years (DALYs) are presented as counts in thousands with corresponding age-standardized rates per 100,000 population and 95% uncertainty intervals (UI). Estimates were obtained from the Global Burden of Disease (GBD) 2023 study. Age-standardized rates were calculated using the GBD world standard population to enable comparisons across regions and SDI categories.

**Table 2:** Temporal trends in age-standardized incidence, mortality, and disability-adjusted life year (DALY) rates for prostate, testicular, kidney, and bladder cancers across global, regional, and Sociodemographic Index (SDI) groups in the Americas, 2000–2023.

Prostate Cancer				
DALYs				
Location	APC	LCL	UCL	N (years)
Global	−1.03	−1.15	−0.92	24
High SDI	−1.58	−1.68	−1.48	24
High-income North America	−1.70	−1.92	−1.48	24
High-middle SDI	−0.97	−1.07	−0.87	24
Latin America and the Caribbean	−0.63	−0.72	−0.53	24
Low SDI	0.61	0.40	0.83	24
Low-middle SDI	0.50	0.35	0.65	24
Middle SDI	0.38	0.29	0.48	24
Mortality				
Location	APC	LCL	UCL	N (years)
Global	−1.15	−1.26	−1.03	24
High SDI	−1.68	−1.79	−1.56	24
High-income North America	−1.82	−2.05	−1.59	24
High-middle SDI	−1.21	−1.29	−1.13	24
Latin America and the Caribbean	−0.64	−0.74	−0.55	24
Low SDI	0.56	0.35	0.77	24
Low-middle SDI	0.55	0.44	0.67	24
Middle SDI	0.31	0.21	0.40	24
Incidence				
Location	APC	LCL	UCL	N (years)
Global	−0.51	−0.60	−0.41	24
High SDI	−0.70	−0.81	−0.59	24
High-income North America	−1.41	−1.58	−1.24	24
High-middle SDI	0.25	0.13	0.37	24
Latin America and the Caribbean	0.28	0.17	0.39	24
Low SDI	1.20	0.95	1.45	24
Low-middle SDI	1.39	1.24	1.54	24
Middle SDI	1.35	1.23	1.46	24
**Testicular Cancer**				
DALYs				
Location	APC	LCL	UCL	N (years)
Global	−0.05	−0.29	0.19	24
High SDI	−0.82	−1.07	−0.56	24
High-income North America	0.45	0.14	0.77	24
High-middle SDI	4.58	4.35	4.91	24
Latin America and the Caribbean	2.60	2.35	2.84	24
Low SDI	0.27	−0.05	0.61	24
Low-middle SDI	0.34	0.18	0.51	24
Middle SDI	0.97	0.87	1.06	24
Mortality				
Location	APC	LCL	UCL	N (years)
Global	−0.29	−0.54	−0.04	24
High SDI	−1.11	−1.36	−0.86	24
High-income North America	0.22	−0.05	0.50	24
High-middle SDI	0.39	0.01	0.77	24
Latin America and the Caribbean	2.17	1.92	2.42	24
Low SDI	0.30	−0.03	0.63	24
Low-middle SDI	0.37	0.21	0.54	24
Middle SDI	0.78	0.67	0.89	24
Incidence				
Location	APC	LCL	UCL	N (years)
Global	0.95	0.79	1.12	24
High SDI	0.83	0.65	1.01	24
High-income North America	0.80	0.50	1.09	24
High-middle SDI	3.63	3.27	4.00	24
Latin America and the Caribbean	4.25	4.01	4.49	24
Low SDI	2.12	1.68	2.55	24
Low-middle SDI	2.28	2.11	2.44	24
Middle SDI	3.32	3.14	3.49	24
**Kidney Cancer**				
DALYs				
Location	APC	LCL	UCL	N (years)
Global	−0.81	−0.88	−0.73	24
High SDI	−1.20	−1.31	−1.09	24
High-income North America	−1.31	−1.40	−1.22	24
High-middle SDI	−0.11	−0.21	−0.01	24
Latin America and the Caribbean	0.61	0.52	0.70	24
Low SDI	0.90	0.81	0.99	24
Low-middle SDI	0.89	0.78	0.99	24
Middle SDI	0.68	0.57	0.79	24
Mortality				
Location	APC	LCL	UCL	N (years)
Global	−0.62	−0.70	−0.53	24
High SDI	−0.83	−0.93	−0.72	24
High-income North America	−0.98	−1.08	−0.88	24
High-middle SDI	0.09	−0.01	0.20	24
Latin America and the Caribbean	0.84	0.75	0.92	24
Low SDI	0.86	0.78	0.95	24
Low-middle SDI	0.90	0.79	1.00	24
Middle SDI	0.95	0.81	1.08	24
Incidence				
Location	APC	LCL	UCL	N (years)
Global	−0.17	−0.33	−0.01	24
High SDI	−0.26	−0.45	−0.06	24
High-income North America	−1.04	−1.19	−0.88	24
High-middle SDI	1.10	0.99	1.22	24
Latin America and the Caribbean	1.45	1.34	1.56	24
Low SDI	1.53	1.45	1.62	24
Low-middle SDI	1.55	1.43	1.67	24
Middle SDI	1.64	1.56	1.73	24
**Bladder Cancer**				
DALYs				
Location	APC	LCL	UCL	N (years)
Global	−1.16	−1.30	−1.03	24
High SDI	−1.36	−1.43	−1.29	24
High-income North America	−0.69	−0.85	−0.53	24
High-middle SDI	−1.14	−1.41	−0.86	24
Latin America and the Caribbean	−0.23	−0.28	−0.18	24
Low SDI	0.36	0.06	0.66	24
Low-middle SDI	0.12	−0.07	0.32	24
Middle SDI	−0.59	−0.96	−0.22	24
Mortality				
Location	APC	LCL	UCL	N (years)
Global	−0.93	−1.05	−0.82	24
High SDI	−1.03	−1.09	−0.96	24
High-income North America	−0.29	−0.44	−0.14	24
High-middle SDI	−0.97	−1.23	−0.71	24
Latin America and the Caribbean	−0.10	−0.15	−0.05	24
Low SDI	0.31	0.04	0.58	24
Low-middle SDI	0.10	−0.07	0.28	24
Middle SDI	−0.32	−0.66	0.01	24
Incidence				
Location	APC	LCL	UCL	N (years)
Global	−0.69	−0.76	−0.62	24
High SDI	−0.73	−0.80	−0.66	24
High-income North America	−0.66	−0.83	−0.48	24
High-middle SDI	−0.13	−0.40	0.13	24
Latin America and the Caribbean	0.19	0.13	0.25	24
Low SDI	0.78	0.44	1.12	24
Low-middle SDI	0.63	0.40	0.86	24
Middle SDI	0.34	−0.03	0.73	24

Annual percentage change (APC) in age-standardized incidence rates (ASIR), mortality rates, and disability-adjusted life year (DALY) rates for prostate, testicular, kidney, and bladder cancers across global, regional, and Sociodemographic Index (SDI) groups from 2000 to 2023. APC estimates are presented with corresponding lower confidence limits (LCL) and upper confidence limits (UCL) based on weighted log-linear regression models. Negative APC values indicate decreasing temporal trends, whereas positive APC values indicate increasing trends over time.

## Data Availability

All data are publicly available via GBD 2023 on the Global Health Data Exchange (http://ghdx.healthdata.org). Analytical code available from corresponding author upon request.

## Supplementary Materials

The following supporting information can be downloaded at: https://www.mdpi.com/article/10.3390/cancers18122016/s1, Table S1: Sociodemographic Index (SDI) groups by location; Table S2: incidence, mortality, and DALY counts of prostate cancer in the Americas, 2000–2023 (95% UI); Table S3: incidence, mortality, and DALY counts of testicular cancer in the Americas, 2000–2023 (95% UI); Table S4: incidence, mortality, and DALY counts of kidney cancer in the Americas by sex, 2000–2023 (95% UI); Table S5: incidence, mortality, and DALY counts of bladder cancer in the Americas by sex, 2000–2023 (95% UI); Table S6: Age-standardized incidence rates of prostate cancer in the Americas, 2000–2023 (95% UI); Table S7: Age-standardized incidence rates of testicular cancer in the Americas, 2000–2023 (95% UI); Table S8: Age-standardized incidence rates of kidney cancer in the Americas by sex, 2000–2023 (95% UI); Table S9: Age-standardized incidence rates of bladder cancer in the Americas by sex, 2000–2023 (95% UI); Table S10: Age-standardized mortality rates of prostate cancer in the Americas, 2000–2023 (95% UI); Table S11: Age-standardized mortality rates of testicular cancer in the Americas, 2000–2023 (95% UI); Table S12: Age-standardized mortality rates of kidney cancer in the Americas by sex, 2000–2023 (95% UI); Table S13: Age-standardized mortality rates of bladder cancer in the Americas by sex, 2000–2023 (95% UI); Table S14: Methodological approach used for annual percentage change (APC) estimation through to fitted log-linear model; Table S15: GATHER checklist for transparent reporting of health estimates; Table S16: Mortality-to-incidence ratios (MIRs) according to region and SDI level, 2023; Table S17: Interval-specific annual percentage changes (APCs) in age-standardized incidence, mortality, and disability-adjusted life year (DALY) rates for prostate, testicular, kidney, and bladder cancers across global, regional, and Sociodemographic Index (SDI) groups in the Americas; Figure S1: Interval-specific annual percentage changes (APCs) in age-standardized incidence, mortality, and disability-adjusted life year (DALY) rates for prostate cancer across global, regional, and Sociodemographic Index (SDI) groups in the Americas; Figure S2: Interval-specific annual percentage changes (APCs) in age-standardized incidence, mortality, and disability-adjusted life year (DALY) rates for testicular cancer across global, regional, and Sociodemographic Index (SDI) groups in the Americas; Figure S3: Interval-specific annual percentage changes (APCs) in age-standardized incidence, mortality, and disability-adjusted life year (DALY) rates for kidney cancer across global, regional, and Sociodemographic Index (SDI) groups in the Americas; Figure S4: Interval-specific annual percentage changes (APCs) in age-standardized incidence, mortality, and disability-adjusted life year (DALY) rates for bladder cancer across global, regional, and Sociodemographic Index (SDI) groups in the Americas.
