# Supplementary material for: Burden and Trends of Genitourinary Cancers Across the Americas: A GBD 2023 Analysis of Regional Socioeconomic Gradients
Source: Cancers (Basel). 2026 Jun 22;18(12):2016. doi: 10.3390/cancers18122016 (PMC13296783; doi:10.3390/cancers18122016)

Supplementary Material

Table S1. Sociodemographic index (SDI) groups by location.

Location	SDI group
Antigua and Barbuda	High SDI
Argentina	High SDI
Barbados	High SDI
Bermuda	High SDI
Canada	High SDI
Chile	High SDI
Dominica	High SDI
Greenland	High SDI
Puerto Rico	High SDI
Saint Kitts and Nevis	High SDI
The Bahamas	High SDI
Trinidad and Tobago	High SDI
Uruguay	High SDI
USA	High SDI
Brazil	High-Middle SDI
Colombia	High-Middle SDI

Table S2. Incidence, Mortality and DALYs counts of Prostate cancer — Americas 2000 - 2023 (UI 95 %)

Location	2000 Incidence	2000 Mortality	2000 DALYs	2023 Incidence	2023 Mortality	2023 DALYs
**Antigua and Barbuda**	45<br>(36, 55)	23<br>(19, 27)	395<br>(338, 461)	77<br>(56, 104)	29<br>(24, 35)	556<br>(453, 674)
**Argentina**	7,819<br>(6,632, 9,431)	4,850<br>(4,402, 5,365)	88,457<br>(80,724, 98,398)	10,994<br>(8,975, 13,428)	5,748<br>(4,938, 6,529)	100,398<br>(87,087, 113,125)
**Bahamas**	110<br>(88, 138)	52<br>(45, 58)	1,043<br>(897, 1,178)	274<br>(215, 355)	117<br>(99, 136)	2,276<br>(1,913, 2,674)
**Barbados**	146<br>(114, 188)	76<br>(64, 89)	1,232<br>(1,043, 1,453)	365<br>(277, 481)	141<br>(117, 171)	2,529<br>(2,120, 3,051)
**Belize**	41<br>(32, 50)	25<br>(21, 29)	451<br>(388, 527)	96<br>(76, 125)	46<br>(39, 54)	849<br>(719, 1,006)
**Bermuda**	60<br>(44, 79)	17<br>(14, 19)	308<br>(260, 359)	111<br>(82, 147)	26<br>(22, 31)	463<br>(385, 556)
**Bolivia (Plurinational State of)**	768<br>(498, 1,090)	453<br>(295, 646)	8,827<br>(5,867, 12,366)	3,632<br>(2,446, 5,188)	1,409<br>(992, 1,905)	28,466<br>(19,892, 39,081)
**Brazil**	22,123<br>(18,443, 26,885)	11,254<br>(10,235, 12,286)	219,541<br>(201,343, 240,229)	52,603<br>(42,765, 65,146)	22,025<br>(19,652, 24,062)	408,779<br>(371,272, 445,107)
**Canada**	15,323<br>(12,452, 18,839)	4,516<br>(4,149, 4,835)	82,457<br>(75,653, 89,395)	21,698<br>(17,114, 26,961)	6,437<br>(5,615, 7,177)	108,039<br>(94,173, 120,385)
**Chile**	2,607<br>(2,253, 3,070)	1,545<br>(1,443, 1,656)	27,193<br>(25,423, 28,825)	6,257<br>(5,184, 7,615)	2,765<br>(2,528, 2,936)	46,127<br>(42,705, 48,855)
**Colombia**	5,922<br>(4,843, 7,214)	2,338<br>(2,178, 2,492)	44,830<br>(42,106, 47,943)	14,241<br>(11,232, 17,727)	4,102<br>(3,677, 4,514)	75,949<br>(68,029, 84,623)
**Costa Rica**	921<br>(738, 1,119)	323<br>(295, 351)	5,733<br>(5,273, 6,209)	2,647<br>(2,100, 3,362)	700<br>(618, 780)	12,646<br>(11,251, 13,996)
**Cuba**	4,927<br>(4,028, 5,963)	2,008<br>(1,858, 2,148)	35,634<br>(33,221, 38,259)	10,620<br>(8,103, 13,488)	3,554<br>(3,017, 4,178)	63,008<br>(53,952, 71,703)
**Dominica**	44<br>(30, 59)	29<br>(21, 39)	478<br>(344, 633)	69<br>(47, 92)	40<br>(29, 54)	689<br>(499, 921)
**Dominican Republic**	1,498<br>(1,114, 1,942)	935<br>(708, 1,151)	16,663<br>(12,730, 20,152)	4,250<br>(2,728, 5,779)	2,495<br>(1,620, 3,448)	43,074<br>(27,971, 58,294)
**Ecuador**	2,150<br>(1,751, 2,588)	1,031<br>(934, 1,124)	18,134<br>(16,510, 19,740)	5,033<br>(4,029, 6,359)	1,693<br>(1,515, 1,896)	29,407<br>(26,456, 32,689)
**El Salvador**	749<br>(565, 988)	364<br>(286, 475)	6,512<br>(5,181, 8,331)	1,885<br>(1,265, 2,797)	732<br>(548, 1,035)	12,943<br>(9,717, 18,581)
**Greenland**	4<br>(2, 6)	2<br>(1, 3)	49<br>(29, 73)	9<br>(5, 14)	4<br>(3, 6)	83<br>(50, 120)
**Grenada**	53<br>(43, 67)	31<br>(26, 36)	556<br>(478, 647)	88<br>(69, 115)	41<br>(35, 50)	802<br>(668, 978)
**Guatemala**	881<br>(723, 1,085)	508<br>(449, 569)	9,388<br>(8,272, 10,504)	2,181<br>(1,702, 2,721)	1,077<br>(910, 1,266)	18,751<br>(15,972, 21,744)

Location	2000 Incidence	2000 Mortality	2000 DALYs	2023 Incidence	2023 Mortality	2023 DALYs
**Guyana**	126<br>(106, 152)	84<br>(74, 94)	1,675<br>(1,491, 1,881)	288<br>(222, 380)	161<br>(133, 199)	3,309<br>(2,736, 4,052)
**Haiti**	782<br>(486, 1,138)	627<br>(395, 916)	12,411<br>(7,786, 18,084)	1,836<br>(1,148, 2,735)	1,316<br>(839, 1,991)	27,055<br>(17,280, 39,509)
**Honduras**	205<br>(137, 301)	140<br>(95, 211)	2,383<br>(1,663, 3,545)	633<br>(423, 886)	426<br>(290, 605)	6,512<br>(4,478, 9,153)
**Jamaica**	1,175<br>(981, 1,468)	623<br>(555, 698)	10,999<br>(9,967, 12,412)	1,955<br>(1,510, 2,495)	876<br>(749, 1,038)	16,001<br>(13,451, 19,200)
**Mexico**	10,697<br>(8,632, 12,989)	4,759<br>(4,313, 5,130)	88,581<br>(81,115, 96,640)	23,085<br>(18,663, 28,199)	8,904<br>(8,220, 9,503)	162,325<br>(151,647, 173,561)
**Nicaragua**	357<br>(255, 477)	162<br>(115, 203)	3,066<br>(2,182, 3,807)	998<br>(735, 1,351)	307<br>(230, 399)	6,123<br>(4,772, 7,767)
**Panama**	726<br>(610, 876)	279<br>(254, 302)	4,993<br>(4,571, 5,410)	1,612<br>(1,325, 1,944)	476<br>(415, 535)	8,500<br>(7,552, 9,518)
**Paraguay**	519<br>(333, 682)	311<br>(203, 389)	5,623<br>(3,673, 6,990)	1,195<br>(717, 1,593)	585<br>(349, 720)	10,864<br>(6,632, 13,368)
**Peru**	3,381<br>(2,140, 4,352)	1,386<br>(969, 1,739)	25,177<br>(17,373, 31,336)	12,884<br>(8,999, 18,170)	3,744<br>(2,781, 4,913)	62,938<br>(46,885, 81,827)
**Puerto Rico**	1,603<br>(1,319, 1,934)	674<br>(614, 728)	11,278<br>(10,390, 12,203)	2,211<br>(1,733, 2,781)	669<br>(583, 747)	11,511<br>(10,094, 12,862)
**Saint Kitts and Nevis**	23<br>(19, 28)	16<br>(13, 18)	256<br>(219, 299)	34<br>(26, 44)	17<br>(14, 19)	308<br>(256, 366)
**Saint Lucia**	70<br>(56, 88)	38<br>(32, 43)	683<br>(583, 786)	140<br>(109, 179)	63<br>(51, 75)	1,163<br>(963, 1,403)
**Saint Vincent and the Grenadines**	48<br>(38, 59)	30<br>(26, 34)	521<br>(450, 596)	98<br>(76, 123)	52<br>(43, 62)	936<br>(783, 1,118)
**Suriname**	70<br>(51, 90)	45<br>(33, 55)	856<br>(604, 1,055)	162<br>(113, 206)	97<br>(69, 117)	1,775<br>(1,238, 2,140)
**Trinidad and Tobago**	452<br>(394, 526)	269<br>(244, 294)	4,926<br>(4,462, 5,360)	1,178<br>(939, 1,500)	526<br>(441, 610)	9,923<br>(8,291, 11,530)
**United States of America**	217,315<br>(175,847, 264,383)	38,104<br>(34,352, 41,658)	755,028<br>(667,932, 844,735)	290,195<br>(235,815, 347,463)	44,824<br>(38,496, 50,728)	917,788<br>(799,662, 1,034,277)
**Uruguay**	1,277<br>(1,113, 1,508)	742<br>(691, 791)	13,152<br>(12,260, 14,012)	1,669<br>(1,375, 2,030)	806<br>(726, 882)	13,912<br>(12,687, 15,133)
**Venezuela (Bolivarian Republic of)**	3,833<br>(3,146, 4,674)	1,580<br>(1,453, 1,702)	31,287<br>(28,868, 33,650)	12,106<br>(9,521, 15,242)	4,551<br>(3,841, 5,372)	90,214<br>(76,567, 106,073)

Abbreviation: DALYs = disability-adjusted life years.

Table S3. Incidence, Mortality and DALYs counts of Testicular cancer — Americas 2000 - 2023 (UI 95 %)

Location	2000 Incidence	2000 Mortality	2000 DALYs	2023 Incidence	2023 Mortality	2023 DALYs
**Antigua and Barbuda**	0<br>(0, 0)	0<br>(0, 0)	3<br>(2, 3)	1<br>(0, 1)	0<br>(0, 0)	4<br>(3, 5)
**Argentina**	1,168<br>(866, 1,517)	191<br>(167, 216)	10,129<br>(8,833, 11,421)	2,837<br>(1,979, 3,832)	301<br>(268, 340)	16,453<br>(14,323, 18,815)
**Bahamas**	0<br>(0, 0)	0<br>(0, 0)	3<br>(2, 4)	1<br>(1, 1)	0<br>(0, 0)	6<br>(5, 8)
**Barbados**	1<br>(1, 2)	0<br>(0, 0)	9<br>(7, 11)	1<br>(1, 2)	0<br>(0, 0)	9<br>(7, 11)
**Belize**	1<br>(1, 1)	0<br>(0, 0)	10<br>(8, 12)	3<br>(2, 4)	0<br>(0, 1)	27<br>(21, 34)
**Bermuda**	0<br>(0, 0)	0<br>(0, 0)	1<br>(1, 2)	1<br>(1, 1)	0<br>(0, 0)	2<br>(2, 3)
**Bolivia (Plurinational State of)**	45<br>(25, 76)	17<br>(10, 28)	913<br>(510, 1,545)	166<br>(88, 264)	43<br>(25, 66)	2,013<br>(1,153, 3,085)
**Brazil**	1,172<br>(879, 1,560)	250<br>(218, 283)	13,521<br>(11,858, 15,387)	4,038<br>(3,009, 5,381)	591<br>(527, 665)	30,349<br>(27,081, 33,980)
**Canada**	1,124<br>(777, 1,585)	46<br>(40, 54)	2,582<br>(2,153, 3,069)	1,686<br>(1,221, 2,276)	57<br>(48, 69)	3,191<br>(2,555, 4,041)
**Chile**	881<br>(661, 1,168)	111<br>(99, 123)	6,172<br>(5,489, 6,891)	1,816<br>(1,207, 2,584)	127<br>(109, 149)	6,892<br>(5,754, 8,296)
**Colombia**	500<br>(368, 653)	80<br>(71, 90)	4,597<br>(4,078, 5,215)	1,875<br>(1,358, 2,457)	168<br>(153, 186)	9,559<br>(8,445, 10,934)
**Costa Rica**	68<br>(49, 95)	8<br>(7, 9)	434<br>(356, 517)	305<br>(212, 416)	23<br>(19, 28)	1,274<br>(1,037, 1,582)
**Cuba**	118<br>(84, 162)	17<br>(14, 20)	770<br>(633, 915)	156<br>(108, 209)	23<br>(18, 28)	797<br>(631, 979)
**Dominica**	0<br>(0, 0)	0<br>(0, 0)	1<br>(1, 2)	0<br>(0, 0)	0<br>(0, 0)	2<br>(1, 3)
**Dominican Republic**	9<br>(6, 15)	2<br>(2, 4)	129<br>(82, 201)	48<br>(29, 81)	11<br>(8, 18)	529<br>(343, 853)
**Ecuador**	205<br>(153, 272)	46<br>(39, 55)	2,432<br>(2,058, 2,891)	479<br>(338, 642)	63<br>(52, 76)	3,346<br>(2,763, 4,063)
**El Salvador**	43<br>(29, 63)	8<br>(6, 11)	475<br>(348, 662)	97<br>(57, 156)	13<br>(9, 20)	715<br>(461, 1,105)
**Greenland**	1<br>(0, 1)	0<br>(0, 0)	7<br>(5, 10)	1<br>(0, 1)	0<br>(0, 0)	5<br>(3, 7)
**Grenada**	1<br>(0, 1)	0<br>(0, 0)	6<br>(5, 8)	1<br>(1, 1)	0<br>(0, 0)	9<br>(7, 11)
**Guatemala**	91<br>(66, 123)	25<br>(21, 29)	1,447<br>(1,199, 1,702)	302<br>(214, 411)	55<br>(44, 68)	3,157<br>(2,462, 3,892)

Location	2000 Incidence	2000 Mortality	2000 DALYs	2023 Incidence	2023 Mortality	2023 DALYs
**Guyana**	2<br>(2, 3)	1<br>(1, 1)	46<br>(38, 57)	6<br>(4, 8)	2<br>(1, 2)	78<br>(62, 96)
**Haiti**	9<br>(5, 18)	4<br>(2, 8)	243<br>(119, 468)	39<br>(19, 67)	15<br>(8, 26)	842<br>(434, 1,472)
**Honduras**	15<br>(8, 27)	5<br>(3, 8)	276<br>(151, 468)	43<br>(21, 75)	10<br>(6, 17)	572<br>(298, 949)
**Jamaica**	9<br>(6, 12)	2<br>(2, 2)	86<br>(71, 105)	29<br>(20, 41)	4<br>(4, 6)	221<br>(170, 278)
**Mexico**	1,879<br>(1,388, 2,540)	357<br>(306, 415)	20,135<br>(17,339, 23,288)	6,321<br>(4,694, 8,235)	866<br>(748, 1,022)	49,461<br>(42,819, 57,903)
**Nicaragua**	38<br>(23, 59)	7<br>(5, 11)	427<br>(264, 628)	135<br>(70, 222)	16<br>(10, 25)	904<br>(532, 1,426)
**Panama**	34<br>(24, 47)	5<br>(4, 5)	257<br>(215, 306)	103<br>(71, 142)	9<br>(8, 11)	505<br>(413, 614)
**Paraguay**	37<br>(23, 56)	10<br>(7, 14)	504<br>(349, 715)	124<br>(78, 187)	25<br>(17, 35)	1,245<br>(828, 1,778)
**Peru**	309<br>(209, 452)	67<br>(50, 91)	3,469<br>(2,574, 4,693)	1,170<br>(732, 1,676)	139<br>(104, 175)	6,745<br>(4,973, 8,613)
**Puerto Rico**	57<br>(40, 79)	7<br>(6, 8)	338<br>(278, 403)	91<br>(63, 124)	8<br>(7, 10)	335<br>(273, 416)
**Saint Kitts and Nevis**	0<br>(0, 0)	0<br>(0, 0)	4<br>(3, 4)	1<br>(0, 1)	0<br>(0, 0)	5<br>(4, 6)
**Saint Lucia**	1<br>(1, 1)	0<br>(0, 0)	10<br>(8, 13)	2<br>(2, 3)	0<br>(0, 0)	19<br>(16, 24)
**Saint Vincent and the Grenadines**	0<br>(0, 1)	0<br>(0, 0)	6<br>(5, 8)	1<br>(1, 1)	0<br>(0, 0)	8<br>(7, 10)
**Suriname**	2<br>(1, 2)	0<br>(0, 1)	25<br>(16, 36)	4<br>(2, 6)	1<br>(1, 2)	50<br>(30, 77)
**Trinidad and Tobago**	6<br>(4, 8)	1<br>(1, 2)	74<br>(61, 87)	11<br>(8, 16)	2<br>(2, 3)	94<br>(75, 117)
**United States of America**	9,028<br>(6,212, 12,597)	456<br>(385, 533)	25,532<br>(21,196, 30,870)	12,125<br>(8,404, 15,988)	591<br>(494, 732)	31,867<br>(25,576, 40,354)
**Uruguay**	100<br>(71, 136)	14<br>(11, 16)	702<br>(590, 842)	209<br>(135, 293)	19<br>(15, 23)	1,007<br>(805, 1,220)
**Venezuela (Bolivarian Republic of)**	191<br>(140, 261)	34<br>(30, 39)	1,991<br>(1,733, 2,296)	292<br>(169, 416)	52<br>(35, 70)	2,657<br>(1,725, 3,612)

Abbreviation: DALYs = disability-adjusted life years.

Table S4. Incidence, Mortality and DALYs counts of Kidney cancer — Americas 2000 - 2023, males, females and both sexes (UI 95 %)

Location	Sex	2000 Incidence	2000 Mortality	2000 DALYs	2023 Incidence	2023 Mortality	2023 DALYs
**Antigua and Barbuda**	Male	1<br>(1, 1)	1<br>(0, 1)	18<br>(14, 21)	2<br>(1, 2)	1<br>(1, 1)	24<br>(19, 28)
Antigua and Barbuda	Female	1<br>(1, 1)	0<br>(0, 0)	10<br>(8, 12)	1<br>(1, 2)	1<br>(0, 1)	16<br>(13, 18)
Antigua and Barbuda	Both	2<br>(1, 2)	1<br>(1, 1)	27<br>(23, 32)	3<br>(3, 4)	1<br>(1, 2)	39<br>(34, 45)
**Argentina**	Male	1,926<br>(1,743, 2,127)	1,220<br>(1,133, 1,307)	32,843<br>(30,639, 35,166)	3,571<br>(3,160, 4,006)	2,013<br>(1,841, 2,186)	51,380<br>(47,115, 55,973)
Argentina	Female	938<br>(822, 1,078)	577<br>(512, 634)	14,171<br>(12,735, 15,692)	1,714<br>(1,525, 1,956)	908<br>(819, 987)	21,964<br>(19,993, 23,947)
Argentina	Both	2,864<br>(2,581, 3,158)	1,797<br>(1,684, 1,899)	47,014<br>(44,461, 49,689)	5,285<br>(4,704, 5,871)	2,921<br>(2,712, 3,117)	73,345<br>(68,231, 78,079)
**Bahamas**	Male	5<br>(4, 6)	3<br>(2, 3)	95<br>(79, 115)	12<br>(10, 14)	5<br>(5, 6)	174<br>(147, 207)
Bahamas	Female	4<br>(3, 5)	2<br>(1, 2)	59<br>(47, 70)	10<br>(8, 12)	4<br>(3, 5)	127<br>(103, 150)
Bahamas	Both	9<br>(8, 11)	4<br>(4, 5)	154<br>(134, 179)	21<br>(18, 25)	9<br>(8, 11)	302<br>(263, 341)
**Barbados**	Male	8<br>(6, 10)	4<br>(4, 5)	120<br>(99, 146)	17<br>(14, 22)	9<br>(7, 11)	220<br>(183, 268)
Barbados	Female	5<br>(4, 7)	3<br>(2, 3)	69<br>(55, 84)	11<br>(8, 13)	5<br>(4, 5)	118<br>(97, 139)
Barbados	Both	13<br>(11, 16)	7<br>(6, 8)	190<br>(164, 214)	28<br>(23, 33)	13<br>(11, 15)	338<br>(291, 389)
**Belize**	Male	3<br>(2, 4)	2<br>(1, 2)	56<br>(45, 67)	7<br>(5, 8)	3<br>(3, 4)	105<br>(85, 130)
Belize	Female	2<br>(2, 3)	1<br>(1, 1)	39<br>(31, 47)	6<br>(4, 7)	2<br>(2, 3)	77<br>(64, 92)
Belize	Both	5<br>(4, 6)	2<br>(2, 3)	94<br>(80, 108)	12<br>(10, 15)	5<br>(5, 6)	182<br>(155, 209)
**Bermuda**	Male	3<br>(2, 4)	1<br>(1, 2)	37<br>(30, 43)	5<br>(4, 6)	2<br>(1, 2)	43<br>(35, 52)
Bermuda	Female	2<br>(1, 2)	1<br>(1, 1)	17<br>(13, 20)	2<br>(2, 3)	1<br>(1, 1)	17<br>(14, 20)
Bermuda	Both	5<br>(4, 6)	2<br>(2, 2)	54<br>(46, 61)	7<br>(6, 8)	3<br>(2, 3)	60<br>(51, 69)
**Bolivia (Plurinational State of)**	Male	115<br>(75, 175)	56<br>(36, 87)	1,928<br>(1,241, 2,998)	309<br>(197, 470)	135<br>(87, 201)	4,057<br>(2,638, 6,057)
Bolivia (Plurinational State of)	Female	126<br>(74, 202)	53<br>(31, 80)	1,890<br>(1,105, 2,941)	299<br>(179, 490)	109<br>(63, 179)	3,431<br>(2,017, 5,617)

Location	Sex	2000 Incidence	2000 Mortality	2000 DALYs	2023 Incidence	2023 Mortality	2023 DALYs
Bolivia (Plurinational State of)	Both	241<br>(164, 341)	108<br>(75, 154)	3,818<br>(2,618, 5,400)	608<br>(425, 835)	244<br>(171, 327)	7,488<br>(5,248, 9,991)
**Brazil**	Male	2,290<br>(2,028, 2,555)	1,268<br>(1,175, 1,358)	41,014<br>(37,401, 44,453)	6,461<br>(5,768, 7,256)	3,437<br>(3,210, 3,670)	91,118<br>(85,775, 96,640)
Brazil	Female	1,820<br>(1,540, 2,202)	863<br>(773, 972)	28,779<br>(25,293, 32,732)	4,220<br>(3,669, 4,833)	2,016<br>(1,825, 2,160)	52,756<br>(48,885, 56,280)
Brazil	Both	4,110<br>(3,636, 4,700)	2,131<br>(1,983, 2,277)	69,793<br>(64,883, 75,181)	10,682<br>(9,486, 12,018)	5,454<br>(5,113, 5,717)	143,874<br>(137,203, 150,847)
**Canada**	Male	3,215<br>(2,777, 3,677)	918<br>(873, 962)	23,009<br>(21,871, 24,105)	5,302<br>(4,236, 6,456)	1,597<br>(1,375, 1,826)	34,815<br>(30,238, 39,991)
Canada	Female	1,294<br>(1,121, 1,484)	563<br>(513, 611)	12,336<br>(11,385, 13,202)	1,719<br>(1,483, 2,018)	777<br>(672, 874)	14,951<br>(13,314, 16,710)
Canada	Both	4,509<br>(3,901, 5,142)	1,480<br>(1,399, 1,550)	35,345<br>(33,636, 37,026)	7,021<br>(5,861, 8,344)	2,374<br>(2,113, 2,621)	49,766<br>(44,576, 55,382)
**Chile**	Male	568<br>(510, 641)	335<br>(304, 365)	9,165<br>(8,394, 9,910)	1,694<br>(1,483, 1,905)	839<br>(782, 902)	20,827<br>(19,267, 22,350)
Chile	Female	303<br>(260, 356)	172<br>(152, 192)	4,363<br>(3,877, 4,866)	828<br>(680, 962)	398<br>(347, 446)	8,977<br>(7,991, 9,954)
Chile	Both	871<br>(782, 990)	507<br>(470, 543)	13,528<br>(12,605, 14,577)	2,522<br>(2,193, 2,829)	1,237<br>(1,136, 1,326)	29,803<br>(27,595, 31,854)
**Colombia**	Male	407<br>(358, 459)	201<br>(182, 222)	6,768<br>(6,196, 7,345)	1,171<br>(1,011, 1,325)	536<br>(491, 577)	14,278<br>(13,230, 15,290)
Colombia	Female	320<br>(271, 375)	139<br>(126, 151)	4,587<br>(4,157, 4,990)	832<br>(711, 951)	337<br>(300, 369)	8,878<br>(8,166, 9,646)
Colombia	Both	727<br>(643, 826)	340<br>(315, 363)	11,355<br>(10,618, 12,066)	2,003<br>(1,753, 2,264)	873<br>(809, 935)	23,156<br>(21,847, 24,537)
**Costa Rica**	Male	60<br>(50, 73)	30<br>(25, 35)	856<br>(723, 1,003)	192<br>(153, 236)	83<br>(67, 98)	2,156<br>(1,741, 2,576)
Costa Rica	Female	51<br>(40, 64)	22<br>(18, 26)	636<br>(523, 759)	145<br>(114, 178)	55<br>(46, 64)	1,415<br>(1,162, 1,644)
Costa Rica	Both	111<br>(93, 131)	52<br>(46, 60)	1,492<br>(1,313, 1,688)	337<br>(277, 397)	138<br>(120, 156)	3,571<br>(3,134, 4,033)
**Cuba**	Male	262<br>(219, 308)	131<br>(115, 150)	3,734<br>(3,242, 4,246)	619<br>(496, 761)	289<br>(239, 345)	7,142<br>(5,947, 8,481)
Cuba	Female	170<br>(138, 221)	75<br>(62, 90)	2,113<br>(1,732, 2,530)	406<br>(312, 505)	169<br>(138, 204)	4,099<br>(3,336, 4,881)
Cuba	Both	433<br>(371, 516)	206<br>(185, 232)	5,847<br>(5,221, 6,595)	1,026<br>(840, 1,215)	459<br>(393, 527)	11,241<br>(9,677, 12,870)
**Dominica**	Male	1<br>(1, 2)	1<br>(0, 1)	22<br>(15, 31)	2<br>(1, 3)	1<br>(1, 2)	34<br>(22, 49)
Dominica	Female	1<br>(1, 1)	0<br>(0, 1)	12<br>(8, 17)	1<br>(1, 2)	1<br>(0, 1)	18<br>(12, 26)

Location	Sex	2000 Incidence	2000 Mortality	2000 DALYs	2023 Incidence	2023 Mortality	2023 DALYs
Dominica	Both	2<br>(1, 3)	1<br>(1, 2)	34<br>(25, 45)	3<br>(2, 5)	2<br>(1, 3)	52<br>(37, 69)
**Dominican Republic**	Male	56<br>(36, 80)	30<br>(20, 43)	1,076<br>(686, 1,545)	104<br>(65, 166)	55<br>(35, 89)	1,732<br>(1,081, 2,737)
Dominican Republic	Female	36<br>(22, 55)	17<br>(10, 25)	614<br>(382, 932)	73<br>(45, 124)	34<br>(21, 57)	1,073<br>(661, 1,811)
Dominican Republic	Both	91<br>(63, 119)	46<br>(32, 62)	1,690<br>(1,177, 2,174)	178<br>(122, 267)	89<br>(61, 134)	2,804<br>(1,937, 4,105)
**Ecuador**	Male	194<br>(157, 233)	79<br>(68, 91)	2,547<br>(2,187, 2,936)	454<br>(367, 542)	164<br>(138, 190)	4,634<br>(3,870, 5,461)
Ecuador	Female	161<br>(129, 202)	57<br>(48, 67)	1,837<br>(1,534, 2,169)	333<br>(268, 407)	105<br>(88, 123)	2,959<br>(2,494, 3,482)
Ecuador	Both	355<br>(300, 423)	136<br>(121, 153)	4,384<br>(3,904, 4,909)	787<br>(655, 946)	269<br>(237, 304)	7,593<br>(6,718, 8,590)
**El Salvador**	Male	48<br>(33, 69)	27<br>(19, 36)	847<br>(587, 1,147)	94<br>(60, 139)	46<br>(30, 67)	1,334<br>(864, 1,906)
El Salvador	Female	47<br>(33, 66)	23<br>(16, 32)	730<br>(509, 1,059)	103<br>(68, 150)	46<br>(31, 66)	1,269<br>(852, 1,823)
El Salvador	Both	95<br>(67, 120)	49<br>(37, 64)	1,576<br>(1,205, 1,974)	197<br>(140, 270)	93<br>(65, 121)	2,602<br>(1,809, 3,375)
**Greenland**	Male	3<br>(2, 5)	2<br>(1, 3)	52<br>(33, 74)	5<br>(3, 8)	3<br>(2, 4)	65<br>(41, 91)
Greenland	Female	1<br>(1, 2)	1<br>(0, 1)	20<br>(9, 29)	2<br>(1, 3)	1<br>(0, 2)	24<br>(9, 38)
Greenland	Both	4<br>(3, 6)	2<br>(1, 3)	72<br>(45, 94)	7<br>(4, 10)	4<br>(2, 5)	89<br>(54, 120)
**Grenada**	Male	1<br>(1, 2)	1<br>(1, 1)	27<br>(22, 32)	3<br>(2, 4)	2<br>(1, 2)	44<br>(35, 53)
Grenada	Female	1<br>(1, 1)	1<br>(0, 1)	15<br>(12, 19)	2<br>(1, 2)	1<br>(1, 1)	21<br>(17, 25)
Grenada	Both	2<br>(2, 3)	1<br>(1, 2)	42<br>(37, 48)	4<br>(4, 5)	2<br>(2, 3)	64<br>(55, 74)
**Guatemala**	Male	66<br>(54, 82)	35<br>(28, 42)	1,370<br>(1,105, 1,681)	200<br>(163, 241)	107<br>(86, 129)	3,256<br>(2,617, 3,918)
Guatemala	Female	70<br>(58, 86)	30<br>(26, 35)	1,287<br>(1,084, 1,504)	186<br>(142, 239)	85<br>(69, 106)	2,672<br>(2,153, 3,305)
Guatemala	Both	137<br>(117, 162)	65<br>(57, 73)	2,657<br>(2,345, 3,018)	386<br>(318, 459)	192<br>(163, 224)	5,928<br>(5,096, 6,930)
**Guyana**	Male	8<br>(7, 10)	5<br>(4, 6)	188<br>(156, 222)	13<br>(10, 16)	7<br>(5, 8)	239<br>(189, 293)
Guyana	Female	6<br>(5, 8)	3<br>(2, 4)	125<br>(97, 152)	11<br>(9, 14)	5<br>(4, 7)	185<br>(151, 222)

Location	Sex	2000 Incidence	2000 Mortality	2000 DALYs	2023 Incidence	2023 Mortality	2023 DALYs
Guyana	Both	15 (12, 18)	8 (7, 9)	313 (274, 354)	24 (21, 28)	12 (11, 14)	424 (366, 485)
**Haiti**	Male	63 (40, 102)	39 (24, 63)	1,671 (1,057, 2,674)	130 (84, 206)	73 (45, 109)	3,041 (1,982, 4,591)
Haiti	Female	48 (29, 76)	27 (16, 42)	1,170 (682, 1,852)	106 (61, 168)	54 (31, 83)	2,242 (1,245, 3,518)
Haiti	Both	112 (71, 160)	66 (44, 93)	2,841 (1,874, 4,021)	236 (159, 339)	127 (85, 172)	5,283 (3,531, 7,247)
**Honduras**	Male	19 (12, 28)	11 (8, 17)	394 (265, 594)	43 (26, 68)	26 (16, 41)	741 (462, 1,141)
Honduras	Female	19 (11, 30)	9 (6, 14)	369 (219, 553)	38 (23, 58)	20 (12, 31)	605 (365, 940)
Honduras	Both	38 (26, 53)	21 (15, 29)	763 (535, 1,046)	81 (57, 114)	46 (32, 63)	1,346 (945, 1,817)
**Jamaica**	Male	41 (33, 50)	20 (17, 24)	679 (557, 798)	57 (45, 73)	28 (23, 35)	823 (672, 1,033)
Jamaica	Female	31 (24, 40)	13 (11, 16)	453 (363, 555)	41 (32, 51)	17 (14, 20)	515 (431, 603)
Jamaica	Both	72 (60, 88)	34 (29, 39)	1,132 (973, 1,303)	98 (82, 117)	45 (38, 52)	1,338 (1,148, 1,537)
**Mexico**	Male	1,568 (1,330, 1,826)	871 (758, 986)	27,060 (23,393, 30,643)	5,094 (4,246, 5,962)	2,588 (2,231, 2,972)	73,774 (63,435, 84,723)
Mexico	Female	1,240 (1,002, 1,514)	596 (504, 690)	18,963 (16,081, 22,179)	3,349 (2,775, 3,973)	1,523 (1,288, 1,765)	42,643 (36,105, 49,512)
Mexico	Both	2,808 (2,422, 3,227)	1,467 (1,318, 1,623)	46,023 (41,350, 50,804)	8,442 (7,260, 9,780)	4,111 (3,739, 4,549)	116,418 (106,072, 129,070)
**Nicaragua**	Male	35 (24, 52)	18 (12, 26)	634 (436, 922)	106 (66, 160)	48 (28, 71)	1,472 (899, 2,097)
Nicaragua	Female	31 (21, 45)	13 (9, 19)	492 (336, 732)	83 (54, 133)	33 (21, 52)	994 (626, 1,593)
Nicaragua	Both	66 (48, 87)	31 (22, 41)	1,126 (837, 1,457)	189 (134, 257)	80 (53, 105)	2,465 (1,657, 3,278)
**Panama**	Male	46 (37, 56)	23 (19, 27)	697 (576, 837)	146 (114, 177)	64 (52, 77)	1,746 (1,402, 2,074)
Panama	Female	38 (29, 48)	15 (12, 18)	501 (394, 604)	101 (83, 122)	38 (31, 45)	1,050 (869, 1,240)
Panama	Both	84 (68, 101)	38 (33, 43)	1,198 (1,038, 1,373)	246 (204, 288)	102 (88, 115)	2,796 (2,418, 3,144)
**Paraguay**	Male	52 (37, 71)	30 (21, 41)	968 (673, 1,325)	144 (80, 201)	78 (43, 109)	2,274 (1,295, 3,114)
Paraguay	Female	46 (32, 65)	22 (15, 31)	768 (545, 1,108)	105 (67, 142)	50 (32, 66)	1,481 (925, 1,942)

Location	Sex	2000 Incidence	2000 Mortality	2000 DALYs	2023 Incidence	2023 Mortality	2023 DALYs
Paraguay	Both	97<br>(73, 123)	52<br>(39, 64)	1,736<br>(1,339, 2,186)	249<br>(158, 313)	128<br>(81, 164)	3,755<br>(2,385, 4,746)
**Peru**	Male	495<br>(330, 671)	189<br>(125, 247)	6,112<br>(4,140, 8,220)	1,881<br>(1,322, 2,468)	601<br>(379, 774)	17,221<br>(11,287, 22,203)
Peru	Female	484<br>(347, 678)	154<br>(116, 222)	5,144<br>(3,776, 7,498)	1,479<br>(1,052, 2,592)	404<br>(325, 662)	11,469<br>(8,532, 19,887)
Peru	Both	978<br>(696, 1,255)	344<br>(260, 429)	11,256<br>(8,861, 14,307)	3,360<br>(2,528, 4,516)	1,005<br>(757, 1,278)	28,690<br>(22,734, 37,322)
**Puerto Rico**	Male	117<br>(97, 139)	57<br>(48, 66)	1,602<br>(1,351, 1,871)	190<br>(148, 235)	82<br>(66, 98)	1,933<br>(1,561, 2,329)
Puerto Rico	Female	61<br>(46, 78)	27<br>(21, 33)	724<br>(572, 870)	107<br>(85, 132)	44<br>(36, 53)	955<br>(804, 1,142)
Puerto Rico	Both	177<br>(149, 211)	84<br>(72, 96)	2,326<br>(2,015, 2,655)	296<br>(242, 353)	125<br>(107, 145)	2,888<br>(2,494, 3,317)
**Saint Kitts and Nevis**	Male	1<br>(1, 1)	0<br>(0, 1)	13<br>(11, 16)	1<br>(1, 1)	1<br>(0, 1)	17<br>(14, 20)
Saint Kitts and Nevis	Female	1<br>(0, 1)	0<br>(0, 0)	9<br>(7, 10)	1<br>(1, 1)	0<br>(0, 0)	11<br>(9, 13)
Saint Kitts and Nevis	Both	1<br>(1, 1)	1<br>(1, 1)	21<br>(19, 25)	2<br>(2, 2)	1<br>(1, 1)	27<br>(24, 31)
**Saint Lucia**	Male	2<br>(2, 3)	1<br>(1, 1)	36<br>(29, 44)	4<br>(3, 4)	2<br>(1, 2)	52<br>(42, 65)
Saint Lucia	Female	1<br>(1, 2)	1<br>(0, 1)	19<br>(15, 23)	2<br>(2, 3)	1<br>(1, 1)	27<br>(22, 32)
Saint Lucia	Both	3<br>(3, 4)	2<br>(1, 2)	54<br>(47, 64)	6<br>(5, 7)	3<br>(2, 3)	79<br>(67, 93)
**Saint Vincent and the Grenadines**	Male	1<br>(1, 1)	1<br>(1, 1)	20<br>(16, 25)	2<br>(1, 2)	1<br>(1, 1)	29<br>(23, 36)
Saint Vincent and the Grenadines	Female	1<br>(1, 2)	1<br>(1, 1)	22<br>(17, 27)	2<br>(1, 2)	1<br>(1, 1)	26<br>(21, 31)
Saint Vincent and the Grenadines	Both	2<br>(2, 3)	1<br>(1, 1)	42<br>(36, 48)	4<br>(3, 4)	2<br>(2, 2)	55<br>(46, 63)
**Suriname**	Male	5<br>(3, 7)	3<br>(2, 4)	103<br>(68, 144)	10<br>(7, 15)	6<br>(4, 8)	181<br>(119, 257)
Suriname	Female	3<br>(2, 5)	2<br>(1, 3)	63<br>(40, 93)	8<br>(5, 12)	4<br>(2, 6)	118<br>(72, 184)
Suriname	Both	8<br>(6, 11)	5<br>(4, 7)	166<br>(121, 213)	18<br>(13, 24)	10<br>(7, 13)	299<br>(222, 399)
**Trinidad and Tobago**	Male	22<br>(19, 27)	13<br>(11, 15)	418<br>(355, 494)	42<br>(33, 52)	21<br>(17, 26)	610<br>(496, 742)
Trinidad and Tobago	Female	19<br>(15, 24)	9<br>(7, 11)	310<br>(244, 373)	40<br>(32, 49)	18<br>(15, 22)	518<br>(431, 605)

Location	Sex	2000 Incidence	2000 Mortality	2000 DALYs	2023 Incidence	2023 Mortality	2023 DALYs
Trinidad and Tobago	Both	41<br>(35, 48)	22<br>(19, 25)	727<br>(631, 812)	82<br>(68, 97)	40<br>(35, 46)	1,128<br>(986, 1,304)
**United States of America**	Male	28,884<br>(24,921, 33,378)	8,567<br>(7,882, 9,174)	220,309<br>(204,758, 235,026)	38,817<br>(33,068, 44,903)	11,829<br>(10,613, 13,068)	270,099<br>(244,862, 298,993)
United States of America	Female	18,256<br>(15,099, 21,748)	5,215<br>(4,676, 5,739)	118,371<br>(106,451, 130,691)	19,825<br>(16,371, 23,584)	5,814<br>(5,032, 6,438)	119,934<br>(106,241, 132,470)
United States of America	Both	47,140<br>(40,840, 54,461)	13,782<br>(12,764, 14,652)	338,680<br>(315,997, 360,595)	58,642<br>(50,362, 67,275)	17,644<br>(15,811, 19,171)	390,033<br>(359,807, 422,810)
**Uruguay**	Male	262<br>(232, 294)	161<br>(146, 177)	4,140<br>(3,759, 4,520)	421<br>(360, 488)	229<br>(207, 256)	5,629<br>(4,999, 6,303)
Uruguay	Female	136<br>(110, 169)	82<br>(67, 98)	1,909<br>(1,570, 2,277)	242<br>(196, 289)	130<br>(108, 153)	2,860<br>(2,417, 3,327)
Uruguay	Both	398<br>(358, 454)	243<br>(221, 264)	6,049<br>(5,544, 6,584)	663<br>(577, 753)	360<br>(326, 395)	8,489<br>(7,660, 9,233)
**Venezuela (Bolivarian Republic of)**	Male	346<br>(303, 388)	184<br>(167, 201)	6,031<br>(5,489, 6,557)	723<br>(596, 832)	378<br>(317, 423)	11,080<br>(9,415, 12,505)
Venezuela (Bolivarian Republic of)	Female	271<br>(233, 317)	122<br>(108, 139)	4,155<br>(3,671, 4,752)	561<br>(461, 678)	261<br>(220, 307)	7,537<br>(6,331, 9,007)
Venezuela (Bolivarian Republic of)	Both	617<br>(546, 696)	307<br>(282, 330)	10,186<br>(9,411, 11,018)	1,284<br>(1,092, 1,473)	638<br>(550, 709)	18,618<br>(16,232, 20,725)

Abbreviation: DALYs = disability-adjusted life years.

Table S5. Incidence, Mortality and DALYs counts of Bladder cancer — Americas 2000 - 2023, males, females and both sexes (UI 95 %)

Location	Sex	2000 Incidence	2000 Mortality	2000 DALYs	2023 Incidence	2023 Mortality	2023 DALYs
**Antigua and Barbuda**	Male	2 (1, 2)	1 (1, 1)	16 (13, 19)	3 (2, 3)	1 (1, 2)	28 (23, 34)
Antigua and Barbuda	Female	1 (1, 1)	1 (0, 1)	11 (10, 13)	2 (2, 3)	1 (1, 1)	21 (18, 25)
Antigua and Barbuda	Both	3 (2, 3)	1 (1, 2)	28 (24, 31)	5 (4, 6)	2 (2, 3)	49 (43, 57)
**Argentina**	Male	2,409 (2,133, 2,724)	1,231 (1,146, 1,323)	26,111 (24,262, 28,123)	2,840 (2,470, 3,332)	1,436 (1,267, 1,628)	28,298 (24,954, 32,031)
Argentina	Female	828 (691, 1,019)	449 (397, 495)	8,427 (7,600, 9,323)	959 (779, 1,178)	497 (425, 580)	9,114 (7,880, 10,450)
Argentina	Both	3,237 (2,831, 3,711)	1,680 (1,562, 1,781)	34,538 (32,118, 36,657)	3,800 (3,268, 4,400)	1,933 (1,753, 2,146)	37,412 (33,868, 41,460)
**Bahamas**	Male	4 (3, 5)	2 (2, 2)	50 (42, 60)	11 (9, 13)	5 (4, 6)	115 (94, 142)
Bahamas	Female	2 (2, 3)	1 (1, 1)	28 (24, 33)	6 (5, 7)	3 (3, 4)	66 (55, 78)
Bahamas	Both	7 (6, 8)	3 (3, 4)	79 (69, 89)	17 (14, 20)	8 (7, 9)	181 (156, 211)
**Barbados**	Male	9 (7, 11)	5 (4, 6)	92 (76, 111)	19 (15, 23)	9 (8, 11)	183 (150, 222)
Barbados	Female	5 (4, 7)	3 (3, 4)	55 (46, 66)	10 (8, 13)	6 (5, 7)	103 (85, 123)
Barbados	Both	14 (12, 17)	8 (7, 9)	147 (129, 170)	30 (25, 35)	15 (13, 17)	286 (248, 330)
**Belize**	Male	3 (2, 3)	2 (1, 2)	34 (29, 40)	7 (5, 8)	3 (3, 4)	72 (58, 88)
Belize	Female	2 (1, 2)	1 (1, 1)	22 (18, 26)	5 (4, 7)	2 (2, 3)	55 (44, 69)
Belize	Both	5 (4, 5)	3 (2, 3)	56 (49, 63)	11 (9, 14)	6 (5, 6)	127 (108, 147)
**Bermuda**	Male	7 (6, 9)	3 (2, 3)	58 (48, 66)	13 (10, 16)	5 (4, 6)	93 (75, 112)
Bermuda	Female	2 (2, 3)	1 (1, 1)	21 (17, 24)	4 (3, 5)	2 (2, 2)	32 (26, 38)
Bermuda	Both	9 (8, 11)	4 (4, 4)	78 (68, 88)	17 (14, 20)	7 (6, 8)	125 (107, 143)
**Bolivia (Plurinational State of)**	Male	84 (57, 130)	40 (26, 63)	937 (618, 1,461)	319 (198, 509)	129 (80, 200)	2,937 (1,794, 4,528)
Bolivia (Plurinational State of)	Female	36 (25, 53)	22 (15, 32)	531 (365, 775)	114 (69, 175)	62 (39, 92)	1,471 (936, 2,204)

Location	Sex	2000 Incidence	2000 Mortality	2000 DALYs	2023 Incidence	2023 Mortality	2023 DALYs
Bolivia (Plurinational State of)	Both	120<br>(86, 167)	63<br>(46, 88)	1,468<br>(1,075, 2,048)	433<br>(297, 636)	191<br>(137, 273)	4,408<br>(3,149, 6,189)
**Brazil**	Male	3,505<br>(3,130, 3,973)	1,875<br>(1,753, 1,994)	41,436<br>(38,740, 44,535)	7,920<br>(6,953, 8,877)	4,166<br>(3,818, 4,465)	82,557<br>(77,194, 88,121)
Brazil	Female	1,703<br>(1,414, 2,103)	900<br>(798, 1,013)	20,633<br>(18,508, 22,882)	4,309<br>(3,514, 5,114)	2,292<br>(1,960, 2,499)	46,492<br>(41,629, 50,239)
Brazil	Both	5,208<br>(4,585, 5,902)	2,775<br>(2,577, 2,959)	62,069<br>(58,249, 66,371)	12,229<br>(10,601, 13,942)	6,458<br>(5,818, 6,914)	129,048<br>(119,923, 136,552)
**Canada**	Male	2,969<br>(2,622, 3,372)	1,253<br>(1,168, 1,329)	24,072<br>(22,625, 25,474)	4,888<br>(4,182, 5,769)	2,219<br>(1,999, 2,423)	36,226<br>(32,681, 39,840)
Canada	Female	1,371<br>(1,132, 1,719)	515<br>(456, 563)	8,973<br>(8,144, 9,821)	2,127<br>(1,655, 2,607)	844<br>(715, 941)	13,415<br>(11,683, 15,106)
Canada	Both	4,340<br>(3,771, 5,022)	1,769<br>(1,634, 1,873)	33,046<br>(30,930, 34,807)	7,015<br>(5,906, 8,379)	3,063<br>(2,738, 3,321)	49,641<br>(44,844, 53,720)
**Chile**	Male	467<br>(410, 538)	228<br>(208, 250)	4,781<br>(4,432, 5,212)	937<br>(818, 1,071)	423<br>(387, 469)	8,194<br>(7,537, 8,985)
Chile	Female	312<br>(263, 376)	155<br>(137, 173)	3,034<br>(2,744, 3,369)	505<br>(395, 616)	230<br>(194, 260)	4,148<br>(3,633, 4,617)
Chile	Both	779<br>(682, 910)	382<br>(354, 415)	7,814<br>(7,351, 8,422)	1,442<br>(1,228, 1,704)	653<br>(588, 716)	12,342<br>(11,358, 13,332)
**Colombia**	Male	481<br>(426, 551)	234<br>(215, 254)	5,263<br>(4,873, 5,723)	963<br>(833, 1,101)	448<br>(405, 492)	8,904<br>(8,117, 9,690)
Colombia	Female	293<br>(245, 351)	147<br>(132, 164)	3,265<br>(2,945, 3,630)	475<br>(384, 581)	230<br>(198, 258)	4,537<br>(3,997, 5,043)
Colombia	Both	775<br>(679, 899)	380<br>(357, 407)	8,528<br>(7,974, 9,133)	1,438<br>(1,224, 1,673)	678<br>(613, 735)	13,441<br>(12,327, 14,553)
**Costa Rica**	Male	78<br>(64, 93)	37<br>(32, 44)	759<br>(651, 886)	202<br>(162, 249)	88<br>(73, 105)	1,756<br>(1,457, 2,083)
Costa Rica	Female	35<br>(28, 45)	17<br>(14, 20)	346<br>(295, 402)	72<br>(56, 91)	33<br>(27, 39)	647<br>(537, 777)
Costa Rica	Both	113<br>(95, 134)	54<br>(48, 61)	1,104<br>(981, 1,229)	274<br>(224, 331)	121<br>(105, 139)	2,403<br>(2,083, 2,784)
**Cuba**	Male	674<br>(591, 774)	337<br>(309, 364)	6,589<br>(6,044, 7,119)	1,233<br>(1,001, 1,496)	559<br>(479, 654)	10,959<br>(9,467, 12,789)
Cuba	Female	241<br>(195, 300)	120<br>(103, 135)	2,388<br>(2,057, 2,706)	483<br>(373, 596)	233<br>(194, 276)	4,398<br>(3,692, 5,127)
Cuba	Both	915<br>(800, 1,062)	456<br>(422, 487)	8,977<br>(8,332, 9,595)	1,717<br>(1,415, 2,028)	792<br>(693, 905)	15,358<br>(13,472, 17,316)
**Dominica**	Male	2<br>(1, 2)	1<br>(1, 1)	19<br>(14, 27)	3<br>(2, 4)	2<br>(1, 2)	33<br>(22, 45)
Dominica	Female	1<br>(1, 2)	1<br>(1, 1)	17<br>(13, 23)	2<br>(1, 3)	1<br>(1, 2)	25<br>(17, 36)

Location	Sex	2000 Incidence	2000 Mortality	2000 DALYs	2023 Incidence	2023 Mortality	2023 DALYs
Dominica	Both	3<br>(2, 4)	2<br>(2, 3)	36<br>(29, 46)	5<br>(4, 7)	3<br>(2, 4)	58<br>(44, 73)
**Dominican Republic**	Male	62<br>(45, 85)	36<br>(27, 49)	769<br>(567, 1,045)	181<br>(120, 256)	102<br>(70, 145)	2,123<br>(1,443, 2,982)
Dominican Republic	Female	34<br>(22, 46)	20<br>(14, 27)	417<br>(279, 550)	97<br>(60, 134)	57<br>(36, 76)	1,163<br>(747, 1,535)
Dominican Republic	Both	96<br>(69, 120)	56<br>(42, 71)	1,186<br>(891, 1,505)	279<br>(206, 373)	159<br>(123, 213)	3,286<br>(2,533, 4,338)
**Ecuador**	Male	183<br>(151, 216)	78<br>(69, 88)	1,662<br>(1,450, 1,890)	340<br>(282, 412)	130<br>(115, 148)	2,562<br>(2,242, 2,957)
Ecuador	Female	82<br>(66, 102)	48<br>(41, 56)	1,011<br>(863, 1,171)	140<br>(110, 176)	75<br>(64, 88)	1,508<br>(1,278, 1,786)
Ecuador	Both	265<br>(225, 311)	126<br>(114, 140)	2,673<br>(2,436, 2,988)	479<br>(400, 571)	206<br>(184, 229)	4,070<br>(3,667, 4,529)
**El Salvador**	Male	36<br>(25, 50)	19<br>(13, 26)	416<br>(287, 567)	89<br>(63, 136)	44<br>(32, 64)	923<br>(683, 1,357)
El Salvador	Female	33<br>(23, 46)	18<br>(13, 23)	385<br>(282, 500)	85<br>(59, 114)	45<br>(33, 61)	904<br>(667, 1,225)
El Salvador	Both	69<br>(52, 87)	37<br>(29, 45)	801<br>(645, 972)	174<br>(134, 228)	89<br>(71, 110)	1,826<br>(1,451, 2,254)
**Greenland**	Male	3<br>(2, 4)	1<br>(1, 2)	35<br>(22, 47)	4<br>(3, 6)	2<br>(2, 3)	47<br>(32, 66)
Greenland	Female	1<br>(1, 1)	0<br>(0, 1)	10<br>(8, 14)	2<br>(1, 2)	1<br>(1, 1)	14<br>(10, 20)
Greenland	Both	4<br>(2, 5)	2<br>(1, 3)	45<br>(31, 58)	6<br>(4, 8)	3<br>(2, 4)	61<br>(45, 78)
**Grenada**	Male	2<br>(2, 3)	1<br>(1, 1)	25<br>(21, 30)	4<br>(3, 4)	2<br>(1, 2)	40<br>(32, 49)
Grenada	Female	2<br>(1, 2)	1<br>(1, 1)	18<br>(15, 21)	3<br>(2, 3)	2<br>(1, 2)	30<br>(24, 37)
Grenada	Both	4<br>(3, 4)	2<br>(2, 2)	43<br>(38, 50)	6<br>(5, 7)	3<br>(3, 4)	70<br>(61, 81)
**Guatemala**	Male	42<br>(35, 51)	24<br>(20, 28)	540<br>(459, 649)	82<br>(66, 104)	44<br>(37, 55)	946<br>(779, 1,158)
Guatemala	Female	36<br>(28, 44)	20<br>(16, 24)	479<br>(393, 559)	68<br>(53, 85)	36<br>(29, 44)	818<br>(655, 990)
Guatemala	Both	78<br>(66, 92)	44<br>(38, 49)	1,019<br>(902, 1,146)	150<br>(126, 182)	80<br>(69, 93)	1,764<br>(1,524, 2,038)
**Guyana**	Male	7<br>(6, 9)	4<br>(3, 5)	101<br>(85, 118)	15<br>(12, 19)	8<br>(6, 10)	194<br>(157, 246)
Guyana	Female	3<br>(3, 4)	2<br>(2, 2)	44<br>(37, 52)	6<br>(5, 8)	3<br>(3, 4)	84<br>(65, 105)

Location	Sex	2000 Incidence	2000 Mortality	2000 DALYs	2023 Incidence	2023 Mortality	2023 DALYs
Guyana	Both	10<br>(9, 12)	6<br>(5, 7)	144<br>(125, 163)	21<br>(17, 26)	11<br>(10, 14)	278<br>(234, 333)
**Haiti**	Male	66<br>(44, 100)	44<br>(29, 67)	1,025<br>(690, 1,533)	157<br>(90, 235)	95<br>(55, 148)	2,337<br>(1,354, 3,586)
Haiti	Female	38<br>(24, 60)	25<br>(15, 39)	593<br>(378, 939)	103<br>(57, 170)	61<br>(35, 101)	1,605<br>(930, 2,680)
Haiti	Both	104<br>(74, 144)	69<br>(48, 96)	1,619<br>(1,148, 2,236)	261<br>(180, 360)	156<br>(109, 220)	3,942<br>(2,785, 5,520)
**Honduras**	Male	12<br>(8, 17)	7<br>(5, 11)	156<br>(107, 223)	32<br>(19, 50)	21<br>(12, 32)	380<br>(226, 597)
Honduras	Female	8<br>(5, 11)	4<br>(3, 6)	110<br>(74, 155)	22<br>(13, 34)	13<br>(8, 19)	282<br>(184, 428)
Honduras	Both	20<br>(14, 25)	12<br>(9, 16)	267<br>(200, 344)	54<br>(38, 75)	33<br>(24, 46)	663<br>(472, 911)
**Jamaica**	Male	60<br>(48, 73)	33<br>(28, 39)	665<br>(557, 789)	107<br>(85, 132)	52<br>(43, 64)	1,122<br>(927, 1,363)
Jamaica	Female	38<br>(30, 48)	22<br>(18, 26)	418<br>(356, 493)	65<br>(51, 83)	35<br>(29, 43)	710<br>(570, 871)
Jamaica	Both	97<br>(81, 117)	55<br>(48, 62)	1,083<br>(957, 1,232)	172<br>(142, 205)	88<br>(75, 101)	1,833<br>(1,574, 2,131)
**Mexico**	Male	976<br>(807, 1,172)	501<br>(429, 580)	11,154<br>(9,436, 12,969)	2,102<br>(1,730, 2,600)	1,046<br>(882, 1,237)	22,148<br>(18,695, 26,106)
Mexico	Female	564<br>(454, 702)	294<br>(252, 341)	6,577<br>(5,664, 7,692)	1,043<br>(844, 1,303)	553<br>(467, 652)	11,315<br>(9,492, 13,343)
Mexico	Both	1,539<br>(1,293, 1,801)	795<br>(716, 890)	17,730<br>(15,897, 20,036)	3,146<br>(2,622, 3,755)	1,599<br>(1,411, 1,821)	33,463<br>(29,308, 38,035)
**Nicaragua**	Male	17<br>(12, 23)	9<br>(6, 12)	204<br>(146, 273)	45<br>(29, 66)	20<br>(13, 29)	459<br>(297, 653)
Nicaragua	Female	14<br>(10, 19)	7<br>(5, 9)	162<br>(119, 212)	35<br>(22, 51)	16<br>(11, 23)	368<br>(247, 520)
Nicaragua	Both	31<br>(23, 39)	16<br>(12, 20)	365<br>(282, 458)	80<br>(58, 105)	36<br>(27, 47)	827<br>(625, 1,069)
**Panama**	Male	33<br>(27, 39)	17<br>(14, 19)	338<br>(283, 396)	60<br>(47, 74)	28<br>(23, 34)	552<br>(446, 662)
Panama	Female	20<br>(15, 25)	10<br>(8, 12)	205<br>(173, 243)	37<br>(29, 48)	18<br>(15, 21)	353<br>(297, 423)
Panama	Both	53<br>(44, 63)	26<br>(23, 30)	543<br>(481, 620)	98<br>(79, 118)	46<br>(39, 52)	905<br>(777, 1,034)
**Paraguay**	Male	47<br>(34, 63)	28<br>(21, 37)	576<br>(439, 746)	125<br>(84, 162)	69<br>(46, 90)	1,409<br>(968, 1,809)
Paraguay	Female	17<br>(12, 22)	10<br>(7, 12)	207<br>(157, 262)	42<br>(30, 59)	24<br>(18, 32)	493<br>(361, 661)

Location	Sex	2000 Incidence	2000 Mortality	2000 DALYs	2023 Incidence	2023 Mortality	2023 DALYs
Paraguay	Both	64<br>(51, 78)	38<br>(31, 46)	783<br>(652, 947)	167<br>(123, 212)	93<br>(67, 116)	1,902<br>(1,393, 2,335)
**Peru**	Male	317<br>(250, 389)	131<br>(103, 161)	2,755<br>(2,200, 3,347)	974<br>(780, 1,207)	343<br>(283, 395)	6,662<br>(5,433, 8,161)
Peru	Female	143<br>(105, 193)	81<br>(61, 108)	1,724<br>(1,299, 2,259)	409<br>(293, 562)	216<br>(161, 291)	4,161<br>(3,204, 5,511)
Peru	Both	459<br>(370, 552)	212<br>(170, 255)	4,478<br>(3,605, 5,313)	1,383<br>(1,122, 1,690)	560<br>(478, 662)	10,823<br>(9,250, 12,903)
**Puerto Rico**	Male	176<br>(150, 205)	84<br>(74, 94)	1,672<br>(1,479, 1,880)	281<br>(236, 340)	125<br>(108, 145)	2,308<br>(1,993, 2,636)
Puerto Rico	Female	92<br>(74, 115)	47<br>(40, 54)	876<br>(763, 1,002)	134<br>(106, 167)	69<br>(58, 81)	1,125<br>(953, 1,308)
Puerto Rico	Both	268<br>(230, 313)	131<br>(120, 146)	2,548<br>(2,350, 2,817)	415<br>(347, 497)	195<br>(170, 217)	3,433<br>(3,039, 3,840)
**Saint Kitts and Nevis**	Male	1<br>(1, 1)	1<br>(1, 1)	13<br>(11, 15)	2<br>(1, 2)	1<br>(1, 1)	17<br>(14, 21)
Saint Kitts and Nevis	Female	1<br>(1, 1)	1<br>(0, 1)	10<br>(9, 12)	1<br>(1, 2)	1<br>(1, 1)	14<br>(12, 17)
Saint Kitts and Nevis	Both	2<br>(2, 2)	1<br>(1, 1)	23<br>(20, 26)	3<br>(2, 3)	1<br>(1, 2)	31<br>(28, 35)
**Saint Lucia**	Male	3<br>(3, 4)	2<br>(2, 2)	38<br>(32, 46)	7<br>(6, 9)	4<br>(3, 4)	76<br>(62, 94)
Saint Lucia	Female	2<br>(2, 3)	1<br>(1, 2)	29<br>(23, 34)	4<br>(3, 6)	2<br>(2, 3)	48<br>(38, 60)
Saint Lucia	Both	6<br>(5, 7)	3<br>(3, 4)	67<br>(58, 77)	12<br>(10, 14)	6<br>(5, 7)	124<br>(108, 146)
**Saint Vincent and the Grenadines**	Male	2<br>(2, 3)	1<br>(1, 1)	25<br>(20, 29)	4<br>(3, 5)	2<br>(2, 3)	46<br>(37, 57)
Saint Vincent and the Grenadines	Female	1<br>(1, 1)	1<br>(0, 1)	12<br>(10, 14)	2<br>(1, 2)	1<br>(1, 1)	18<br>(15, 22)
Saint Vincent and the Grenadines	Both	3<br>(3, 4)	2<br>(2, 2)	36<br>(32, 41)	6<br>(5, 7)	3<br>(3, 4)	65<br>(54, 76)
**Suriname**	Male	6<br>(4, 8)	3<br>(2, 5)	77<br>(55, 104)	13<br>(8, 19)	7<br>(5, 11)	160<br>(107, 227)
Suriname	Female	2<br>(1, 3)	1<br>(1, 2)	29<br>(21, 39)	6<br>(4, 8)	3<br>(2, 5)	70<br>(47, 99)
Suriname	Both	8<br>(6, 10)	5<br>(4, 6)	106<br>(81, 136)	19<br>(13, 25)	11<br>(8, 14)	230<br>(168, 297)
**Trinidad and Tobago**	Male	24<br>(20, 30)	13<br>(11, 16)	296<br>(247, 348)	57<br>(46, 71)	28<br>(23, 35)	617<br>(502, 763)
Trinidad and Tobago	Female	12<br>(9, 14)	7<br>(6, 8)	147<br>(126, 174)	26<br>(21, 34)	14<br>(12, 18)	286<br>(237, 357)

Location	Sex	2000 Incidence	2000 Mortality	2000 DALYs	2023 Incidence	2023 Mortality	2023 DALYs
Trinidad and Tobago	Both	36<br>(31, 43)	20<br>(18, 23)	442<br>(389, 498)	84<br>(68, 100)	42<br>(36, 49)	903<br>(775, 1,069)
**United States of America**	Male	43,242<br>(37,669, 48,552)	9,702<br>(8,993, 10,387)	200,817<br>(186,738, 218,349)	64,058<br>(55,890, 71,962)	15,467<br>(13,880, 16,899)	293,124<br>(268,032, 320,440)
United States of America	Female	16,983<br>(13,542, 21,036)	4,566<br>(3,857, 5,155)	83,922<br>(73,069, 93,412)	22,314<br>(17,356, 27,396)	6,062<br>(5,058, 7,022)	110,003<br>(93,462, 126,139)
United States of America	Both	60,225<br>(51,893, 68,363)	14,268<br>(13,083, 15,264)	284,738<br>(263,859, 305,198)	86,373<br>(73,452, 97,367)	21,528<br>(19,031, 23,398)	403,127<br>(365,600, 436,042)
**Uruguay**	Male	415<br>(359, 473)	207<br>(186, 228)	4,198<br>(3,784, 4,639)	467<br>(399, 546)	231<br>(202, 265)	4,385<br>(3,831, 4,946)
Uruguay	Female	122<br>(100, 151)	66<br>(56, 75)	1,153<br>(1,006, 1,305)	164<br>(131, 199)	83<br>(72, 98)	1,444<br>(1,265, 1,694)
Uruguay	Both	537<br>(467, 617)	273<br>(250, 297)	5,350<br>(4,914, 5,853)	631<br>(539, 732)	315<br>(281, 351)	5,829<br>(5,217, 6,469)
**Venezuela (Bolivarian Republic of)**	Male	290<br>(254, 335)	144<br>(130, 159)	3,336<br>(3,065, 3,653)	851<br>(726, 1,003)	431<br>(376, 490)	9,354<br>(8,166, 10,621)
Venezuela (Bolivarian Republic of)	Female	156<br>(129, 187)	80<br>(72, 90)	1,815<br>(1,614, 2,014)	417<br>(327, 545)	224<br>(188, 283)	4,722<br>(3,929, 5,931)
Venezuela (Bolivarian Republic of)	Both	446<br>(386, 511)	224<br>(208, 242)	5,151<br>(4,777, 5,563)	1,268<br>(1,071, 1,488)	654<br>(584, 736)	14,076<br>(12,399, 15,932)

Abbreviation: DALYs = disability-adjusted life years.

Table S6 Prostate cancer — age-standardised incidence, mortality, and DALY rates (2000 and 2023), males (UI 95 %)

Location	2000 Incidence	2000 Mortality	2000 DALYs	2023 Incidence	2023 Mortality	2023 DALYs
**Antigua and Barbuda**	170·14 (136·08, 209·51)	87·19 (73·64, 102·85)	1,497·22 (1,283·03, 1,747·93)	145·08 (107·94, 193·97)	62·96 (52·30, 76·81)	1,095·50 (905·62, 1,310·56)
**Argentina**	48·32 (41·16, 57·94)	32·33 (29·39, 35·78)	551·93 (503·41, 613·79)	41·53 (33·97, 50·66)	22·40 (19·24, 25·46)	382·10 (331·30, 431·24)
**Bahamas**	138·28 (112·05, 169·95)	77·51 (67·62, 86·76)	1,347·89 (1,167·47, 1,518·95)	156·11 (124·47, 198·02)	82·33 (69·33, 95·68)	1,366·33 (1,162·69, 1,593·00)
**Barbados**	106·00 (82·24, 136·92)	53·65 (45·22, 62·74)	883·96 (747·88, 1,042·63)	146·49 (111·38, 191·28)	60·50 (49·90, 73·52)	1,035·86 (865·26, 1,251·43)
**Belize**	71·09 (56·42, 87·01)	46·44 (39·71, 54·04)	788·04 (676·97, 917·04)	68·69 (54·43, 87·20)	38·29 (32·47, 45·95)	629·49 (535·52, 748·11)
**Bermuda**	188·17 (141·41, 247·48)	59·72 (51·32, 70·15)	1,019·08 (857·51, 1,182·35)	170·63 (126·99, 226·47)	43·37 (36·91, 51·14)	730·63 (606·28, 872·77)
**Bolivia (Plurinational State of)**	41·44 (27·08, 58·85)	29·51 (19·08, 42·85)	485·62 (320·28, 688·90)	89·16 (60·40, 127·43)	41·69 (29·28, 56·67)	735·46 (515·14, 1,009·03)
**Brazil**	43·01 (36·28, 51·55)	24·66 (22·31, 27·09)	434·64 (396·09, 476·60)	45·42 (37·13, 55·96)	20·80 (18·37, 22·82)	362·41 (326·69, 394·28)
**Canada**	83·62 (68·15, 102·62)	26·87 (24·68, 28·78)	463·19 (424·56, 500·41)	59·17 (46·59, 73·86)	17·22 (15·02, 19·20)	289·57 (252·02, 322·94)
**Chile**	48·06 (41·52, 56·13)	31·56 (29·31, 33·90)	510·92 (475·80, 543·87)	51·28 (42·80, 62·05)	24·13 (21·95, 25·61)	387·07 (357·22, 409·70)
**Colombia**	52·56 (43·46, 63·28)	23·71 (21·95, 25·40)	412·18 (385·17, 442·47)	54·28 (42·87, 67·47)	16·67 (14·91, 18·36)	296·91 (265·41, 330·39)
**Costa Rica**	80·09 (64·54, 96·69)	31·72 (28·81, 34·35)	519·34 (477·14, 561·52)	92·02 (73·56, 116·37)	27·58 (24·43, 30·74)	463·25 (411·66, 513·12)
**Cuba**	80·18 (65·51, 97·14)	32·37 (29·99, 34·63)	578·15 (538·96, 621·40)	112·80 (85·85, 143·85)	37·43 (31·72, 43·96)	668·28 (572·90, 762·66)
**Dominica**	131·81 (90·74, 178·59)	88·04 (63·61, 117·33)	1,443·37 (1,031·92, 1,914·88)	140·02 (96·31, 187·65)	87·02 (61·99, 117·09)	1,423·30 (1,027·70, 1,909·25)
**Dominican Republic**	60·05 (44·68, 77·68)	38·37 (29·16, 47·27)	668·19 (509·85, 813·92)	99·00 (64·27, 134·89)	63·55 (41·35, 88·13)	1,017·92 (665·52, 1,392·51)
**Ecuador**	63·33 (52·08, 75·65)	33·71 (30·35, 36·90)	549·14 (497·30, 599·03)	60·44 (48·47, 76·19)	21·49 (19·29, 24·02)	360·52 (325·15, 399·94)
**El Salvador**	44·64 (33·74, 58·86)	22·50 (17·61, 29·32)	390·92 (310·81, 500·36)	76·59 (51·59, 113·61)	31·75 (23·60, 44·29)	536·66 (403·98, 764·49)
**Greenland**	32·38 (18·53, 47·86)	25·61 (14·50, 37·92)	399·71 (230·08, 589·96)	29·17 (16·97, 42·96)	19·48 (11·59, 28·00)	295·76 (178·49, 424·23)
**Grenada**	127·73 (102·59, 160·54)	77·33 (66·00, 90·99)	1,340·20 (1,152·83, 1,560·47)	111·70 (88·07, 144·44)	56·82 (47·92, 67·98)	1,035·71 (870·51, 1,255·45)
**Guatemala**	43·10 (35·70, 52·07)	30·16 (26·73, 33·92)	473·38 (417·91, 531·16)	45·31 (35·93, 55·91)	26·34 (22·40, 31·06)	406·97 (348·21, 473·09)

Location	2000 Incidence	2000 Mortality	2000 DALYs	2023 Incidence	2023 Mortality	2023 DALYs
**Guyana**	74·73 (63·48, 88·75)	55·70 (48·49, 62·73)	985·28 (872·93, 1,103·14)	91·73 (71·78, 120·24)	59·31 (48·76, 73·08)	1,066·15 (883·69, 1,306·80)
**Haiti**	48·12 (30·28, 70·37)	43·49 (27·74, 64·16)	741·16 (468·94, 1,073·62)	65·95 (41·43, 97·26)	54·57 (34·12, 84·05)	948·82 (611·95, 1,430·56)
**Honduras**	16·90 (11·29, 25·23)	12·97 (8·66, 19·81)	198·63 (137·59, 297·12)	21·69 (14·53, 30·85)	16·45 (11·03, 23·99)	228·87 (155·57, 322·79)
**Jamaica**	116·53 (97·30, 145·84)	60·96 (54·35, 68·14)	1,085·80 (985·89, 1,227·04)	130·68 (102·25, 165·52)	60·91 (52·52, 72·31)	1,081·46 (915·41, 1,300·67)
**Mexico**	40·70 (33·10, 49·16)	19·80 (17·92, 21·36)	344·34 (314·68, 374·24)	37·59 (30·54, 45·77)	15·65 (14·37, 16·71)	271·00 (252·90, 289·61)
**Nicaragua**	36·27 (25·70, 49·00)	19·04 (13·48, 24·52)	322·38 (228·22, 408·98)	48·68 (36·14, 65·16)	19·18 (13·87, 24·14)	323·53 (245·24, 407·99)
**Panama**	75·04 (63·24, 89·96)	31·76 (28·91, 34·39)	531·47 (483·29, 577·70)	74·76 (61·36, 90·10)	23·24 (20·21, 26·01)	401·66 (356·63, 449·98)
**Paraguay**	39·23 (25·18, 51·45)	25·16 (16·38, 31·32)	428·24 (280·84, 534·09)	51·24 (30·70, 67·58)	29·83 (17·42, 37·60)	485·27 (291·27, 604·10)
**Peru**	44·70 (28·67, 57·55)	19·96 (14·12, 25·04)	341·08 (237·19, 426·13)	75·31 (52·60, 106·00)	24·08 (17·77, 31·56)	382·26 (285·07, 498·01)
**Puerto Rico**	75·42 (62·02, 91·07)	31·43 (28·65, 33·95)	529·02 (487·54, 571·87)	55·06 (43·10, 68·98)	15·57 (13·58, 17·34)	279·76 (246·09, 311·02)
**Saint Kitts and Nevis**	129·78 (105·43, 157·66)	86·56 (73·59, 100·26)	1,424·91 (1,214·39, 1,668·52)	133·80 (106·54, 172·55)	78·40 (65·69, 91·72)	1,279·19 (1,065·74, 1,512·70)
**Saint Lucia**	126·25 (100·82, 158·26)	68·60 (58·95, 79·19)	1,232·22 (1,048·94, 1,417·60)	117·73 (92·21, 150·17)	55·56 (45·06, 66·77)	994·39 (823·60, 1,195·73)
**Saint Vincent and the Grenadines**	127·64 (101·96, 157·26)	81·96 (70·52, 93·93)	1,398·61 (1,215·85, 1,601·92)	116·32 (91·14, 146·66)	63·42 (52·79, 74·37)	1,121·97 (939·56, 1,337·27)
**Suriname**	51·31 (37·66, 65·21)	37·28 (27·82, 45·87)	631·70 (452·89, 772·14)	58·70 (41·34, 73·43)	39·14 (27·77, 47·10)	650·83 (453·90, 787·83)
**Trinidad and Tobago**	108·19 (95·33, 124·64)	77·57 (70·90, 84·47)	1,203·75 (1,089·86, 1,309·58)	139·89 (112·24, 173·85)	81·84 (68·81, 94·43)	1,262·15 (1,064·14, 1,447·13)
**United States of America**	137·08 (110·75, 167·07)	24·80 (22·19, 27·06)	479·00 (424·18, 534·80)	101·54 (82·29, 121·92)	16·22 (13·86, 18·39)	324·37 (282·57, 365·80)
**Uruguay**	67·97 (59·55, 79·81)	41·63 (38·80, 44·40)	707·11 (661·80, 751·71)	69·48 (57·25, 84·72)	33·94 (30·79, 37·07)	580·13 (527·95, 628·53)
**Venezuela (Bolivarian Republic of)**	71·21 (59·59, 85·03)	37·33 (34·35, 40·01)	616·78 (569·84, 663·97)	91·82 (73·37, 113·81)	45·17 (39·84, 51·90)	741·59 (644·27, 859·25)

Abbreviation: DALYs = disability-adjusted life years.

Table S7. Testicular cancer — age-standardised incidence, mortality, and DALY rates (2000 and 2023), males (UI 95 %)

Location	2000 Incidence	2000 Mortality	2000 DALYs	2023 Incidence	2023 Mortality	2023 DALYs
**Antigua and Barbuda**	0·81<br>(0·58, 1·09)	0·16<br>(0·14, 0·20)	7·28<br>(6·08, 9·04)	1·27<br>(0·83, 1·70)	0·18<br>(0·14, 0·22)	8·24<br>(6·53, 10·25)
**Argentina**	6·54<br>(4·86, 8·49)	1·10<br>(0·96, 1·24)	56·66<br>(49·40, 63·92)	11·59<br>(8·08, 15·67)	1·21<br>(1·08, 1·37)	66·98<br>(58·33, 76·76)
**Bahamas**	0·18<br>(0·13, 0·24)	0·04<br>(0·03, 0·05)	1·86<br>(1·53, 2·25)	0·37<br>(0·25, 0·52)	0·06<br>(0·05, 0·07)	2·94<br>(2·30, 3·61)
**Barbados**	0·83<br>(0·60, 1·18)	0·14<br>(0·12, 0·17)	6·54<br>(5·29, 8·12)	0·93<br>(0·64, 1·30)	0·12<br>(0·10, 0·15)	5·43<br>(4·38, 6·72)
**Belize**	0·63<br>(0·46, 0·87)	0·18<br>(0·15, 0·22)	8·49<br>(6·72, 10·24)	1·47<br>(1·05, 1·99)	0·27<br>(0·22, 0·33)	12·83<br>(10·28, 16·29)
**Bermuda**	0·95<br>(0·68, 1·31)	0·08<br>(0·07, 0·10)	3·78<br>(3·08, 4·67)	3·35<br>(2·26, 4·60)	0·14<br>(0·12, 0·16)	8·40<br>(6·79, 10·26)
**Bolivia (Plurinational State of)**	1·25<br>(0·69, 2·12)	0·55<br>(0·31, 0·91)	24·32<br>(13·56, 40·33)	2·78<br>(1·48, 4·37)	0·84<br>(0·48, 1·27)	33·65<br>(19·22, 51·54)
**Brazil**	1·30<br>(0·98, 1·71)	0·31<br>(0·27, 0·35)	15·06<br>(13·22, 17·15)	3·70<br>(2·76, 4·94)	0·53<br>(0·48, 0·60)	27·78<br>(24·76, 31·11)
**Canada**	7·07<br>(4·84, 10·01)	0·27<br>(0·24, 0·32)	15·98<br>(13·30, 19·11)	8·98<br>(6·48, 12·16)	0·25<br>(0·21, 0·30)	16·24<br>(12·95, 20·68)
**Chile**	10·86<br>(8·17, 14·40)	1·45<br>(1·30, 1·60)	76·94<br>(68·55, 85·73)	17·56<br>(11·67, 25·01)	1·19<br>(1·02, 1·39)	66·21<br>(55·27, 79·77)
**Colombia**	2·50<br>(1·85, 3·26)	0·45<br>(0·40, 0·50)	23·25<br>(20·67, 26·40)	6·49<br>(4·73, 8·49)	0·60<br>(0·55, 0·66)	33·29<br>(29·45, 37·95)
**Costa Rica**	3·36<br>(2·41, 4·68)	0·46<br>(0·38, 0·54)	22·00<br>(18·06, 26·10)	10·58<br>(7·37, 14·42)	0·82<br>(0·68, 1·00)	44·24<br>(36·06, 54·89)
**Cuba**	1·80<br>(1·28, 2·46)	0·27<br>(0·22, 0·31)	11·89<br>(9·79, 14·13)	2·54<br>(1·76, 3·45)	0·29<br>(0·24, 0·36)	12·20<br>(9·64, 15·07)
**Dominica**	0·25<br>(0·16, 0·39)	0·07<br>(0·05, 0·10)	3·26<br>(2·14, 4·58)	0·44<br>(0·25, 0·74)	0·10<br>(0·07, 0·17)	4·71<br>(2·86, 7·86)
**Dominican Republic**	0·22<br>(0·13, 0·35)	0·06<br>(0·04, 0·10)	3·07<br>(1·95, 4·73)	0·82<br>(0·50, 1·39)	0·21<br>(0·14, 0·33)	9·16<br>(5·98, 14·66)
**Ecuador**	3·49<br>(2·63, 4·61)	0·88<br>(0·75, 1·04)	41·17<br>(34·88, 48·85)	5·39<br>(3·81, 7·22)	0·73<br>(0·60, 0·87)	37·56<br>(31·03, 45·63)
**El Salvador**	1·60<br>(1·10, 2·33)	0·33<br>(0·25, 0·46)	17·92<br>(13·18, 24·83)	3·05<br>(1·80, 4·90)	0·45<br>(0·30, 0·68)	22·89<br>(14·86, 35·13)
**Greenland**	2·37<br>(1·48, 3·55)	0·59<br>(0·42, 0·80)	23·86<br>(16·47, 32·37)	2·52<br>(1·37, 4·22)	0·34<br>(0·21, 0·49)	14·89<br>(8·97, 22·25)
**Grenada**	0·99<br>(0·74, 1·31)	0·28<br>(0·23, 0·34)	12·02<br>(10·03, 14·88)	1·51<br>(1·01, 2·11)	0·30<br>(0·24, 0·37)	14·15<br>(11·11, 17·47)
**Guatemala**	2·00<br>(1·48, 2·66)	0·63<br>(0·52, 0·74)	31·59<br>(26·17, 37·04)	3·60<br>(2·57, 4·83)	0·74<br>(0·59, 0·90)	37·95<br>(29·98, 46·69)

Location	2000 Incidence	2000 Mortality	2000 DALYs	2023 Incidence	2023 Mortality	2023 DALYs
**Guyana**	0·67<br>(0·52, 0·85)	0·27<br>(0·23, 0·33)	12·31<br>(10·41, 15·24)	1·32<br>(0·88, 1·77)	0·39<br>(0·31, 0·48)	18·44<br>(14·64, 22·83)
**Haiti**	0·26<br>(0·13, 0·51)	0·14<br>(0·07, 0·26)	6·67<br>(3·36, 12·92)	0·59<br>(0·30, 1·03)	0·26<br>(0·14, 0·42)	12·71<br>(6·65, 21·89)
**Honduras**	0·57<br>(0·30, 1·01)	0·20<br>(0·11, 0·34)	10·23<br>(5·59, 17·39)	0·81<br>(0·40, 1·40)	0·22<br>(0·12, 0·36)	10·82<br>(5·67, 17·97)
**Jamaica**	0·68<br>(0·49, 0·92)	0·16<br>(0·13, 0·19)	6·85<br>(5·65, 8·29)	1·85<br>(1·26, 2·57)	0·29<br>(0·23, 0·35)	13·95<br>(10·79, 17·51)
**Mexico**	3·70<br>(2·76, 4·95)	0·81<br>(0·69, 0·94)	40·14<br>(34·50, 46·53)	9·27<br>(6·88, 12·07)	1·29<br>(1·12, 1·53)	72·55<br>(62·77, 84·93)
**Nicaragua**	1·65<br>(1·01, 2·49)	0·38<br>(0·24, 0·55)	18·77<br>(11·86, 27·54)	3·71<br>(1·93, 6·04)	0·51<br>(0·31, 0·79)	25·38<br>(15·09, 39·53)
**Panama**	2·18<br>(1·55, 2·97)	0·33<br>(0·27, 0·38)	16·72<br>(14·01, 19·85)	4·88<br>(3·38, 6·71)	0·45<br>(0·36, 0·54)	23·80<br>(19·45, 28·96)
**Paraguay**	1·59<br>(1·01, 2·37)	0·47<br>(0·33, 0·66)	21·54<br>(14·98, 30·24)	3·71<br>(2·35, 5·58)	0·82<br>(0·56, 1·19)	37·74<br>(25·21, 54·06)
**Peru**	2·51<br>(1·71, 3·64)	0·61<br>(0·46, 0·84)	27·96<br>(21·07, 38·19)	6·17<br>(3·86, 8·83)	0·77<br>(0·57, 0·96)	35·84<br>(26·46, 45·73)
**Puerto Rico**	3·00<br>(2·08, 4·16)	0·35<br>(0·29, 0·41)	17·57<br>(14·45, 20·97)	7·04<br>(4·87, 9·67)	0·41<br>(0·34, 0·52)	23·64<br>(19·06, 29·63)
**Saint Kitts and Nevis**	1·11<br>(0·81, 1·54)	0·34<br>(0·28, 0·42)	14·70<br>(11·90, 18·10)	2·27<br>(1·68, 3·04)	0·48<br>(0·39, 0·57)	21·62<br>(17·48, 26·43)
**Saint Lucia**	1·25<br>(0·91, 1·70)	0·29<br>(0·23, 0·35)	13·63<br>(10·88, 16·30)	2·50<br>(1·74, 3·39)	0·39<br>(0·32, 0·49)	19·75<br>(16·20, 24·55)
**Saint Vincent and the Grenadines**	0·88<br>(0·64, 1·21)	0·25<br>(0·20, 0·30)	11·49<br>(9·08, 14·10)	1·32<br>(0·94, 1·91)	0·28<br>(0·23, 0·35)	13·29<br>(10·63, 16·91)
**Suriname**	0·64<br>(0·39, 0·95)	0·23<br>(0·15, 0·33)	10·54<br>(6·66, 15·11)	1·36<br>(0·79, 2·12)	0·35<br>(0·22, 0·54)	17·15<br>(10·41, 26·54)
**Trinidad and Tobago**	0·84<br>(0·63, 1·09)	0·25<br>(0·21, 0·29)	10·79<br>(9·05, 12·82)	1·58<br>(1·10, 2·17)	0·28<br>(0·23, 0·35)	12·96<br>(10·28, 16·13)
**United States of America**	6·06<br>(4·17, 8·46)	0·30<br>(0·25, 0·35)	17·11<br>(14·18, 20·67)	7·13<br>(4·94, 9·45)	0·31<br>(0·26, 0·38)	18·36<br>(14·69, 23·23)
**Uruguay**	6·24<br>(4·46, 8·53)	0·83<br>(0·70, 0·98)	43·53<br>(36·51, 52·24)	12·70<br>(8·19, 17·86)	1·07<br>(0·87, 1·27)	60·10<br>(47·84, 72·87)
**Venezuela (Bolivarian Republic of)**	1·58<br>(1·17, 2·15)	0·33<br>(0·29, 0·38)	16·69<br>(14·51, 19·28)	2·61<br>(1·50, 3·73)	0·46<br>(0·31, 0·61)	23·49<br>(15·22, 31·45)

Abbreviation: DALYs = disability-adjusted life years.

Table S8. Kidney cancer — age-standardised incidence, mortality, and DALY rates (2000 and 2023), males, females, and both sexes (UI 95 %)

Location	Sex	2000 Incidence	2000 Mortality	2000 DALYs	2023 Incidence	2023 Mortality	2023 DALYs
**Antigua and Barbuda**	Both	2·77 (2·31, 3·34)	1·46 (1·25, 1·68)	42·01 (35·99, 48·32)	2·78 (2·34, 3·27)	1·18 (1·01, 1·34)	35·10 (30·00, 39·64)
Antigua and Barbuda	Female	1·93 (1·52, 2·49)	0·91 (0·71, 1·12)	27·24 (21·12, 33·47)	2·32 (1·84, 2·88)	0·86 (0·70, 1·02)	27·14 (22·21, 32·13)
Antigua and Barbuda	Male	3·78 (3·00, 4·67)	2·12 (1·72, 2·53)	59·43 (48·39, 71·51)	3·31 (2·61, 4·07)	1·56 (1·26, 1·86)	44·03 (35·30, 52·83)
**Argentina**	Both	7·51 (6·77, 8·29)	4·66 (4·36, 4·93)	124·30 (117·51, 131·44)	9·10 (8·12, 10·10)	4·83 (4·49, 5·16)	127·58 (118·86, 135·88)
Argentina	Female	4·44 (3·90, 5·12)	2·61 (2·33, 2·88)	68·16 (61·16, 75·75)	5·42 (4·81, 6·16)	2·64 (2·38, 2·86)	70·25 (64·13, 76·54)
Argentina	Male	11·32 (10·25, 12·48)	7·31 (6·80, 7·83)	191·75 (178·73, 205·13)	13·63 (12·06, 15·35)	7·66 (7·02, 8·33)	196·39 (180·06, 213·86)
**Bahamas**	Both	3·67 (3·10, 4·40)	1·93 (1·68, 2·20)	60·56 (52·74, 69·82)	4·72 (4·06, 5·52)	2·09 (1·82, 2·39)	66·07 (57·47, 74·54)
Bahamas	Female	2·81 (2·20, 3·53)	1·35 (1·07, 1·63)	42·89 (33·93, 51·43)	4·05 (3·25, 4·98)	1·60 (1·30, 1·90)	52·54 (42·67, 61·91)
Bahamas	Male	4·73 (3·87, 5·74)	2·67 (2·24, 3·19)	81·32 (68·24, 98·13)	5·55 (4·61, 6·88)	2·71 (2·30, 3·26)	81·99 (69·56, 97·23)
**Barbados**	Both	4·50 (3·81, 5·34)	2·23 (1·92, 2·53)	65·73 (57·04, 74·42)	6·01 (4·93, 7·09)	2·58 (2·21, 2·96)	73·21 (63·10, 84·12)
Barbados	Female	3·28 (2·54, 4·28)	1·45 (1·14, 1·76)	44·10 (34·57, 53·62)	4·45 (3·55, 5·47)	1·66 (1·36, 1·95)	49·80 (41·26, 58·61)
Barbados	Male	6·07 (4·90, 7·38)	3·28 (2·68, 3·96)	92·35 (75·88, 111·91)	7·92 (6·37, 10·05)	3·73 (3·09, 4·59)	101·07 (84·10, 123·43)
**Belize**	Both	3·13 (2·62, 3·67)	1·79 (1·53, 2·03)	55·31 (47·31, 63·11)	3·53 (2·91, 4·22)	1·65 (1·42, 1·88)	50·69 (43·18, 58·19)
Belize	Female	2·71 (2·07, 3·41)	1·41 (1·11, 1·70)	45·27 (35·52, 54·51)	3·12 (2·45, 3·91)	1·33 (1·08, 1·59)	41·71 (34·41, 49·79)
Belize	Male	3·54 (2·86, 4·24)	2·15 (1·78, 2·57)	64·91 (53·59, 77·83)	3·94 (3·15, 4·78)	1·98 (1·60, 2·41)	59·74 (48·55, 73·60)
**Bermuda**	Both	6·40 (5·25, 7·81)	2·63 (2·29, 3·00)	70·83 (61·37, 80·61)	6·36 (5·29, 7·63)	1·93 (1·63, 2·19)	54·94 (47·14, 62·35)
Bermuda	Female	4·03 (3·06, 5·19)	1·50 (1·18, 1·82)	40·29 (32·18, 48·35)	3·77 (2·97, 4·64)	1·03 (0·85, 1·25)	29·05 (24·28, 34·43)
Bermuda	Male	9·38 (7·40, 11·47)	4·15 (3·39, 4·86)	108·18 (88·80, 125·84)	9·29 (7·46, 11·26)	3·03 (2·43, 3·65)	83·82 (67·32, 99·97)
**Bolivia (Plurinational State of)**	Both	4·12 (2·77, 5·84)	2·17 (1·51, 3·06)	63·68 (44·10, 90·12)	5·76 (4·03, 7·94)	2·48 (1·73, 3·34)	70·57 (49·61, 94·17)
Bolivia (Plurinational State of)	Female	4·04 (2·39, 6·39)	1·96 (1·16, 2·99)	59·70 (35·03, 91·37)	5·32 (3·20, 8·75)	2·05 (1·18, 3·35)	61·09 (35·85, 100·21)

Location	Sex	2000 Incidence	2000 Mortality	2000 DALYs	2023 Incidence	2023 Mortality	2023 DALYs
Bolivia (Plurinational State of)	Male	4·24 (2·80, 6·41)	2·43 (1·59, 3·78)	68·44 (44·71, 106·95)	6·33 (4·04, 9·56)	3·00 (1·93, 4·49)	81·79 (53·48, 121·78)
**Brazil**	Both	2·97 (2·65, 3·39)	1·68 (1·55, 1·80)	49·43 (45·95, 52·97)	4·20 (3·72, 4·74)	2·10 (1·97, 2·20)	56·72 (53·93, 59·50)
Brazil	Female	2·45 (2·09, 2·95)	1·26 (1·12, 1·41)	38·30 (33·88, 43·49)	3·16 (2·74, 3·64)	1·41 (1·28, 1·52)	39·80 (36·55, 42·80)
Brazil	Male	3·60 (3·24, 3·99)	2·19 (2·01, 2·35)	62·33 (57·12, 67·23)	5·49 (4·90, 6·17)	2·97 (2·76, 3·18)	77·03 (72·35, 81·67)
**Canada**	Both	11·31 (9·78, 12·94)	3·54 (3·35, 3·70)	88·69 (84·32, 93·03)	10·29 (8·56, 12·30)	3·02 (2·71, 3·33)	71·60 (64·63, 80·02)
Canada	Female	6·03 (5·26, 6·99)	2·35 (2·16, 2·54)	57·61 (53·48, 61·76)	4·74 (4·07, 5·57)	1·77 (1·55, 1·98)	40·59 (36·06, 45·14)
Canada	Male	17·44 (15·08, 19·96)	5·08 (4·81, 5·34)	124·97 (118·83, 130·95)	16·40 (13·12, 19·97)	4·50 (3·88, 5·12)	105·98 (92·37, 121·69)
**Chile**	Both	6·30 (5·65, 7·14)	3·78 (3·51, 4·05)	97·14 (90·35, 104·92)	9·62 (8·37, 10·84)	4·54 (4·18, 4·86)	113·73 (105·94, 121·17)
Chile	Female	4·06 (3·48, 4·75)	2·32 (2·05, 2·59)	58·48 (51·99, 65·20)	5·86 (4·84, 6·76)	2·60 (2·28, 2·91)	63·49 (57·33, 70·08)
Chile	Male	9·02 (8·11, 10·15)	5·65 (5·13, 6·17)	142·62 (129·93, 153·99)	14·04 (12·31, 15·78)	6·92 (6·48, 7·43)	172·00 (159·75, 183·89)
**Colombia**	Both	2·38 (2·12, 2·68)	1·26 (1·16, 1·35)	36·43 (34·13, 38·84)	3·49 (3·06, 3·96)	1·49 (1·38, 1·59)	40·33 (38·05, 42·54)
Colombia	Female	1·99 (1·69, 2·28)	0·98 (0·88, 1·07)	28·26 (25·72, 30·54)	2·74 (2·34, 3·17)	1·04 (0·93, 1·14)	29·20 (27·05, 31·55)
Colombia	Male	2·81 (2·46, 3·18)	1·57 (1·41, 1·75)	45·35 (41·21, 49·56)	4·43 (3·83, 5·00)	2·05 (1·88, 2·21)	53·75 (49·78, 57·58)
**Costa Rica**	Both	3·93 (3·31, 4·62)	2·01 (1·76, 2·29)	52·45 (46·19, 59·55)	5·59 (4·59, 6·61)	2·27 (1·98, 2·58)	59·11 (51·83, 66·85)
Costa Rica	Female	3·45 (2·70, 4·27)	1·63 (1·34, 1·94)	42·92 (35·44, 51·20)	4·70 (3·71, 5·81)	1·73 (1·43, 2·01)	45·82 (37·59, 53·34)
Costa Rica	Male	4·46 (3·72, 5·41)	2·43 (2·04, 2·85)	62·71 (52·85, 73·98)	6·56 (5·23, 8·08)	2·89 (2·34, 3·44)	73·39 (59·21, 87·51)
**Cuba**	Both	3·53 (3·03, 4·21)	1·63 (1·46, 1·84)	47·87 (42·62, 53·98)	5·72 (4·65, 6·76)	2·34 (2·01, 2·68)	62·52 (53·88, 71·43)
Cuba	Female	2·73 (2·21, 3·54)	1·14 (0·95, 1·37)	33·94 (27·80, 40·72)	4·53 (3·53, 5·62)	1·63 (1·33, 1·95)	45·26 (37·94, 53·61)
Cuba	Male	4·37 (3·66, 5·13)	2·15 (1·87, 2·45)	62·33 (53·99, 71·00)	7·10 (5·67, 8·77)	3·16 (2·61, 3·76)	81·84 (68·28, 97·45)
**Dominica**	Both	2·79 (2·06, 3·69)	1·62 (1·19, 2·19)	48·78 (36·48, 65·28)	3·80 (2·81, 5·16)	1·99 (1·41, 2·65)	59·18 (42·99, 78·26)
Dominica	Female	2·04 (1·37, 2·89)	1·08 (0·75, 1·58)	33·35 (22·81, 47·54)	2·86 (1·89, 4·24)	1·36 (0·91, 2·05)	41·26 (27·08, 61·20)

Location	Sex	2000 Incidence	2000 Mortality	2000 DALYs	2023 Incidence	2023 Mortality	2023 DALYs
Dominica	Male	3·67 (2·55, 5·40)	2·27 (1·50, 3·29)	66·06 (44·99, 95·42)	4·73 (3·16, 6·97)	2·63 (1·69, 3·82)	76·54 (50·49, 109·96)
**Dominican Republic**	Both	1·36 (0·94, 1·80)	0·78 (0·54, 1·06)	24·47 (16·95, 32·26)	1·70 (1·16, 2·55)	0·89 (0·61, 1·34)	26·50 (18·36, 38·73)
Dominican Republic	Female	1·03 (0·62, 1·61)	0·54 (0·34, 0·80)	17·48 (10·87, 26·25)	1·35 (0·83, 2·28)	0·64 (0·39, 1·07)	19·61 (12·07, 32·89)
Dominican Republic	Male	1·70 (1·10, 2·44)	1·02 (0·68, 1·52)	31·62 (20·60, 45·48)	2·09 (1·30, 3·32)	1·16 (0·74, 1·87)	33·97 (21·25, 54·21)
**Ecuador**	Both	3·87 (3·30, 4·56)	1·71 (1·52, 1·92)	47·43 (42·37, 53·34)	4·40 (3·66, 5·29)	1·50 (1·32, 1·70)	42·38 (37·50, 47·93)
Ecuador	Female	3·39 (2·71, 4·21)	1·38 (1·16, 1·63)	38·70 (32·31, 45·66)	3·57 (2·87, 4·36)	1·10 (0·93, 1·29)	31·59 (26·58, 37·17)
Ecuador	Male	4·39 (3·59, 5·24)	2·06 (1·76, 2·39)	56·61 (48·79, 65·61)	5·34 (4·32, 6·38)	1·96 (1·65, 2·27)	54·44 (45·43, 64·14)
**El Salvador**	Both	2·20 (1·57, 2·79)	1·24 (0·93, 1·62)	36·04 (27·47, 45·59)	3·25 (2·30, 4·45)	1·52 (1·07, 1·98)	42·83 (29·89, 55·71)
El Salvador	Female	1·98 (1·40, 2·75)	1·03 (0·74, 1·48)	30·52 (21·33, 44·31)	2·95 (1·96, 4·29)	1·29 (0·87, 1·84)	36·36 (24·42, 52·32)
El Salvador	Male	2·48 (1·72, 3·52)	1·48 (1·05, 2·03)	42·72 (29·87, 57·70)	3·71 (2·37, 5·44)	1·86 (1·22, 2·70)	52·25 (33·85, 74·61)
**Greenland**	Both	10·49 (6·17, 14·14)	6·85 (3·70, 9·52)	161·66 (94·48, 217·97)	10·61 (6·24, 15·01)	6·00 (3·22, 8·52)	128·74 (74·37, 176·76)
Greenland	Female	6·27 (2·72, 9·19)	4·24 (1·71, 6·42)	96·92 (42·25, 143·15)	5·98 (2·38, 10·06)	3·53 (1·21, 5·85)	74·79 (28·87, 121·00)
Greenland	Male	15·15 (8·87, 22·60)	10·11 (5·39, 15·60)	229·27 (132·23, 335·60)	14·81 (8·75, 21·30)	8·43 (4·74, 12·27)	177·22 (106·19, 247·98)
**Grenada**	Both	2·63 (2·25, 3·13)	1·47 (1·27, 1·68)	45·12 (39·28, 51·47)	2·97 (2·50, 3·56)	1·49 (1·25, 1·73)	44·39 (38·37, 51·32)
Grenada	Female	1·96 (1·55, 2·46)	0·99 (0·77, 1·21)	31·57 (25·12, 37·84)	2·15 (1·74, 2·62)	0·95 (0·77, 1·15)	29·68 (24·37, 35·36)
Grenada	Male	3·41 (2·75, 4·14)	2·06 (1·66, 2·46)	60·43 (48·70, 71·97)	3·80 (3·05, 4·79)	2·05 (1·65, 2·55)	58·91 (47·64, 72·19)
**Guatemala**	Both	1·84 (1·59, 2·13)	1·12 (0·97, 1·26)	33·94 (29·90, 38·45)	3·05 (2·51, 3·65)	1·64 (1·39, 1·91)	46·02 (39·21, 53·85)
Guatemala	Female	1·75 (1·45, 2·12)	0·98 (0·84, 1·14)	30·68 (26·09, 35·47)	2·71 (2·08, 3·47)	1·35 (1·10, 1·69)	38·58 (31·17, 48·03)
Guatemala	Male	1·94 (1·59, 2·37)	1·26 (1·04, 1·51)	37·48 (30·68, 45·44)	3·44 (2·80, 4·13)	1·98 (1·59, 2·39)	54·72 (44·02, 65·79)
**Guyana**	Both	2·62 (2·22, 3·10)	1·63 (1·41, 1·84)	53·09 (46·28, 59·88)	3·04 (2·63, 3·58)	1·63 (1·40, 1·89)	53·41 (46·05, 60·87)
Guyana	Female	2·11 (1·61, 2·64)	1·21 (0·94, 1·47)	40·79 (31·80, 49·39)	2·75 (2·18, 3·43)	1·38 (1·13, 1·68)	45·27 (37·08, 54·66)

Location	Sex	2000 Incidence	2000 Mortality	2000 DALYs	2023 Incidence	2023 Mortality	2023 DALYs
Guyana	Male	3·17 (2·59, 3·81)	2·10 (1·73, 2·46)	66·16 (54·61, 77·76)	3·37 (2·64, 4·17)	1·91 (1·48, 2·35)	62·05 (48·95, 75·52)
**Haiti**	Both	1·89 (1·24, 2·72)	1·31 (0·88, 1·83)	43·82 (29·07, 62·07)	2·29 (1·56, 3·23)	1·44 (0·97, 1·97)	47·93 (32·30, 64·95)
Haiti	Female	1·57 (0·95, 2·49)	1·03 (0·64, 1·60)	35·23 (21·07, 54·46)	1·92 (1·11, 3·04)	1·12 (0·64, 1·73)	38·54 (21·81, 60·85)
Haiti	Male	2·24 (1·39, 3·68)	1·62 (1·01, 2·68)	52·86 (33·44, 85·28)	2·72 (1·72, 4·25)	1·80 (1·10, 2·72)	58·33 (36·66, 86·62)
**Honduras**	Both	0·97 (0·68, 1·31)	0·65 (0·47, 0·90)	18·63 (13·36, 26·13)	1·04 (0·73, 1·43)	0·65 (0·45, 0·89)	16·87 (11·78, 22·86)
Honduras	Female	0·91 (0·53, 1·42)	0·55 (0·32, 0·85)	16·78 (9·94, 25·18)	0·91 (0·55, 1·39)	0·51 (0·31, 0·82)	14·07 (8·53, 22·22)
Honduras	Male	1·05 (0·70, 1·61)	0·75 (0·49, 1·16)	20·61 (13·78, 31·45)	1·20 (0·74, 1·92)	0·79 (0·49, 1·24)	20·01 (12·35, 31·19)
**Jamaica**	Both	3·11 (2·61, 3·81)	1·52 (1·31, 1·75)	49·21 (42·29, 56·77)	3·21 (2·67, 3·84)	1·42 (1·20, 1·65)	43·98 (37·74, 50·51)
Jamaica	Female	2·59 (2·03, 3·35)	1·13 (0·90, 1·38)	38·03 (30·53, 46·41)	2·62 (2·10, 3·29)	1·03 (0·85, 1·22)	33·29 (27·75, 39·12)
Jamaica	Male	3·68 (3·01, 4·55)	1·95 (1·61, 2·29)	61·02 (50·04, 71·94)	3·86 (2·99, 4·90)	1·86 (1·51, 2·36)	55·41 (44·94, 69·30)
**Mexico**	Both	4·07 (3·52, 4·67)	2·36 (2·13, 2·61)	65·38 (58·97, 72·15)	6·05 (5·20, 7·01)	2·98 (2·71, 3·30)	82·92 (75·51, 91·92)
Mexico	Female	3·36 (2·73, 4·04)	1·82 (1·54, 2·10)	50·78 (43·25, 59·04)	4·57 (3·78, 5·43)	2·07 (1·75, 2·40)	58·05 (49·10, 67·34)
Mexico	Male	4·86 (4·15, 5·64)	2·96 (2·58, 3·36)	81·43 (70·91, 91·97)	7·76 (6·47, 9·08)	4·04 (3·47, 4·65)	111·28 (95·60, 127·89)
**Nicaragua**	Both	2·16 (1·58, 2·84)	1·21 (0·88, 1·62)	35·64 (26·23, 47·39)	3·40 (2·42, 4·59)	1·58 (1·03, 2·08)	43·96 (29·42, 58·07)
Nicaragua	Female	1·85 (1·21, 2·71)	0·94 (0·64, 1·38)	28·75 (19·64, 42·09)	2·74 (1·80, 4·41)	1·16 (0·73, 1·86)	32·77 (20·55, 52·73)
Nicaragua	Male	2·51 (1·73, 3·73)	1·51 (1·03, 2·22)	43·29 (29·59, 63·35)	4·24 (2·57, 6·39)	2·12 (1·24, 3·15)	57·70 (34·61, 83·18)
**Panama**	Both	3·55 (2·90, 4·23)	1·77 (1·53, 2·02)	50·59 (43·82, 57·96)	5·51 (4·56, 6·45)	2·24 (1·94, 2·52)	62·45 (53·95, 70·25)
Panama	Female	3·12 (2·43, 3·89)	1·39 (1·12, 1·67)	41·28 (32·69, 49·75)	4·41 (3·62, 5·37)	1·59 (1·32, 1·87)	45·96 (37·99, 54·41)
Panama	Male	4·00 (3·21, 4·85)	2·17 (1·80, 2·59)	59·95 (49·70, 71·77)	6·72 (5·26, 8·15)	2·97 (2·39, 3·56)	80·22 (64·47, 95·37)
**Paraguay**	Both	2·64 (1·98, 3·32)	1·61 (1·21, 1·99)	45·74 (34·82, 57·24)	4·29 (2·71, 5·41)	2·32 (1·45, 2·96)	63·98 (40·58, 81·05)
Paraguay	Female	2·30 (1·61, 3·23)	1·26 (0·88, 1·77)	37·93 (26·92, 54·20)	3·46 (2·21, 4·67)	1·68 (1·08, 2·23)	48·43 (30·17, 63·56)

Location	Sex	2000 Incidence	2000 Mortality	2000 DALYs	2023 Incidence	2023 Mortality	2023 DALYs
Paraguay	Male	3·03<br>(2·14, 4·13)	1·99<br>(1·37, 2·67)	54·18<br>(37·72, 73·52)	5·27<br>(2·92, 7·36)	3·08<br>(1·67, 4·32)	81·69<br>(46·11, 112·31)
**Peru**	Both	4·94<br>(3·47, 6·28)	1·98<br>(1·48, 2·45)	57·18<br>(44·05, 72·36)	9·17<br>(6·92, 12·33)	2·74<br>(2·06, 3·47)	77·70<br>(61·73, 101·11)
Peru	Female	4·71<br>(3·44, 6·62)	1·71<br>(1·28, 2·46)	50·82<br>(38·18, 73·78)	7·95<br>(5·60, 13·97)	2·10<br>(1·69, 3·45)	60·99<br>(45·13, 106·06)
Peru	Male	5·20<br>(3·42, 6·95)	2·28<br>(1·51, 2·95)	63·90<br>(42·86, 84·81)	10·51<br>(7·38, 13·80)	3·44<br>(2·16, 4·42)	95·43<br>(62·52, 122·53)
**Puerto Rico**	Both	4·01<br>(3·36, 4·79)	1·82<br>(1·56, 2·07)	52·81<br>(45·72, 60·28)	5·04<br>(4·06, 6·08)	1·65<br>(1·42, 1·91)	49·11<br>(42·21, 56·82)
Puerto Rico	Female	2·54<br>(1·94, 3·29)	1·05<br>(0·83, 1·28)	30·41<br>(24·07, 36·48)	3·36<br>(2·69, 4·18)	1·02<br>(0·85, 1·21)	29·44<br>(24·98, 34·69)
Puerto Rico	Male	5·74<br>(4·75, 6·88)	2·75<br>(2·32, 3·18)	78·95<br>(66·45, 92·22)	6·95<br>(5·37, 8·71)	2·40<br>(1·94, 2·88)	71·24<br>(57·26, 86·56)
**Saint Kitts and Nevis**	Both	3·08<br>(2·60, 3·69)	1·85<br>(1·61, 2·13)	53·39<br>(46·91, 61·34)	3·17<br>(2·72, 3·73)	1·64<br>(1·42, 1·84)	46·68<br>(40·97, 52·76)
Saint Kitts and Nevis	Female	2·43<br>(1·87, 3·09)	1·33<br>(1·05, 1·60)	39·68<br>(31·48, 48·13)	2·55<br>(2·08, 3·16)	1·19<br>(0·98, 1·41)	34·92<br>(29·07, 41·21)
Saint Kitts and Nevis	Male	3·88<br>(3·19, 4·71)	2·50<br>(2·07, 3·01)	69·24<br>(57·99, 83·05)	3·88<br>(3·24, 4·64)	2·17<br>(1·76, 2·59)	59·78<br>(49·36, 71·07)
**Saint Lucia**	Both	2·51<br>(2·12, 2·97)	1·35<br>(1·16, 1·57)	41·86<br>(36·27, 48·90)	2·47<br>(2·10, 2·90)	1·11<br>(0·94, 1·30)	34·98<br>(29·53, 40·88)
Saint Lucia	Female	1·79<br>(1·35, 2·27)	0·88<br>(0·68, 1·08)	27·46<br>(21·32, 33·16)	1·84<br>(1·45, 2·31)	0·75<br>(0·62, 0·88)	23·71<br>(19·56, 27·75)
Saint Lucia	Male	3·33<br>(2·69, 4·18)	1·89<br>(1·55, 2·35)	57·72<br>(46·64, 70·94)	3·13<br>(2·56, 3·91)	1·48<br>(1·21, 1·88)	46·55<br>(38·06, 58·41)
**Saint Vincent and the Grenadines**	Both	2·62<br>(2·18, 3·10)	1·47<br>(1·26, 1·67)	45·67<br>(39·37, 52·02)	2·52<br>(2·09, 2·96)	1·20<br>(1·01, 1·39)	38·14<br>(32·12, 44·04)
Saint Vincent and the Grenadines	Female	2·75<br>(2·12, 3·49)	1·42<br>(1·13, 1·76)	45·70<br>(36·53, 56·58)	2·65<br>(2·08, 3·25)	1·17<br>(0·92, 1·41)	37·58<br>(30·23, 45·30)
Saint Vincent and the Grenadines	Male	2·52<br>(2·06, 3·07)	1·53<br>(1·25, 1·85)	46·04<br>(37·44, 55·64)	2·37<br>(1·90, 2·97)	1·23<br>(0·99, 1·52)	38·50<br>(30·74, 48·00)
**Suriname**	Both	2·33<br>(1·68, 3·12)	1·44<br>(1·04, 1·98)	44·33<br>(32·27, 57·88)	2·77<br>(2·03, 3·78)	1·46<br>(1·08, 1·98)	46·43<br>(34·41, 62·27)
Suriname	Female	1·81<br>(1·14, 2·65)	1·04<br>(0·66, 1·59)	32·79<br>(20·64, 48·38)	2·23<br>(1·39, 3·47)	1·07<br>(0·65, 1·69)	35·13<br>(21·31, 55·66)
Suriname	Male	2·89<br>(1·91, 4·20)	1·88<br>(1·28, 2·72)	56·61<br>(37·44, 80·39)	3·41<br>(2·31, 4·92)	1·95<br>(1·31, 2·77)	59·17<br>(38·84, 84·31)
**Trinidad and Tobago**	Both	3·71<br>(3·16, 4·30)	2·12<br>(1·85, 2·37)	64·16<br>(55·66, 71·74)	4·56<br>(3·78, 5·37)	2·16<br>(1·88, 2·50)	63·14<br>(55·12, 72·77)
Trinidad and Tobago	Female	3·27<br>(2·55, 4·15)	1·72<br>(1·37, 2·07)	53·25<br>(42·04, 64·17)	4·39<br>(3·51, 5·37)	1·90<br>(1·57, 2·22)	56·79<br>(47·05, 66·62)

Location	Sex	2000 Incidence	2000 Mortality	2000 DALYs	2023 Incidence	2023 Mortality	2023 DALYs
Trinidad and Tobago	Male	4·21 (3·50, 5·00)	2·58 (2·20, 3·04)	75·73 (64·29, 89·40)	4·80 (3·87, 5·95)	2·49 (2·03, 3·03)	70·34 (57·06, 85·71)
**United States of America**	Both	13·38 (11·54, 15·50)	3·71 (3·45, 3·94)	97·26 (90·98, 103·87)	10·67 (9·10, 12·27)	2·92 (2·63, 3·17)	71·14 (65·61, 77·19)
United States of America	Female	9·46 (7·79, 11·29)	2·40 (2·16, 2·62)	61·79 (55·09, 68·38)	6·92 (5·68, 8·30)	1·72 (1·51, 1·89)	41·36 (36·64, 45·76)
United States of America	Male	18·17 (15·66, 21·00)	5·42 (4·97, 5·81)	139·24 (129·33, 148·70)	14·91 (12·66, 17·28)	4·35 (3·90, 4·82)	104·23 (93·89, 116·29)
**Uruguay**	Both	9·75 (8·75, 11·16)	5·65 (5·16, 6·16)	151·18 (138·47, 164·59)	13·17 (11·37, 15·06)	6·50 (5·87, 7·13)	171·69 (155·76, 187·51)
Uruguay	Female	6·00 (4·85, 7·48)	3·27 (2·70, 3·91)	86·15 (70·45, 102·40)	8·53 (6·94, 10·18)	3·95 (3·31, 4·63)	102·82 (87·98, 119·01)
Uruguay	Male	14·41 (12·72, 16·22)	8·75 (7·95, 9·59)	229·91 (208·55, 251·12)	18·99 (16·14, 22·03)	9·95 (8·95, 11·10)	256·00 (225·92, 286·85)
**Venezuela (Bolivarian Republic of)**	Both	3·85 (3·41, 4·31)	2·22 (2·03, 2·38)	61·75 (56·85, 66·69)	4·26 (3·61, 4·86)	2·12 (1·83, 2·35)	61·50 (53·54, 68·68)
Venezuela (Bolivarian Republic of)	Female	3·13 (2·74, 3·64)	1·65 (1·45, 1·87)	47·17 (41·69, 53·75)	3·60 (2·96, 4·39)	1·62 (1·37, 1·91)	48·41 (40·89, 57·69)
Venezuela (Bolivarian Republic of)	Male	4·69 (4·13, 5·26)	2·89 (2·63, 3·16)	78·30 (71·32, 85·65)	5·07 (4·18, 5·84)	2·76 (2·28, 3·10)	76·72 (64·29, 86·59)

Abbreviation: DALYs = disability-adjusted life years.

Table S9. Bladder cancer — age-standardised incidence, mortality, and DALY rates (2000 and 2023), males, females, and both sexes (UI 95 %)

Location	Sex	2000 Incidence	2000 Mortality	2000 DALYs	2023 Incidence	2023 Mortality	2023 DALYs
**Antigua and Barbuda**	Both	4·22 (3·53, 5·00)	2·30 (2·00, 2·60)	45·55 (39·66, 51·27)	4·16 (3·46, 4·94)	2·06 (1·77, 2·38)	42·14 (36·70, 48·74)
Antigua and Barbuda	Female	3·09 (2·51, 3·89)	1·69 (1·42, 1·98)	33·51 (28·59, 39·37)	3·32 (2·62, 4·09)	1·65 (1·36, 1·94)	34·19 (28·66, 39·79)
Antigua and Barbuda	Male	5·63 (4·52, 6·86)	3·08 (2·55, 3·67)	60·45 (50·18, 72·10)	5·16 (4·01, 6·38)	2·58 (2·10, 3·21)	51·59 (42·01, 63·26)
**Argentina**	Both	8·33 (7·29, 9·53)	4·33 (4·03, 4·59)	89·28 (83·03, 94·97)	6·14 (5·27, 7·12)	3·00 (2·72, 3·33)	60·76 (55·08, 67·30)
Argentina	Female	3·68 (3·07, 4·55)	1·94 (1·72, 2·13)	37·94 (34·36, 42·01)	2·70 (2·18, 3·35)	1·27 (1·09, 1·48)	25·98 (22·55, 29·66)
Argentina	Male	14·64 (13·02, 16·50)	7·88 (7·34, 8·44)	158·27 (147·19, 170·01)	10·76 (9·35, 12·62)	5·53 (4·88, 6·26)	107·17 (94·52, 120·99)
**Bahamas**	Both	3·22 (2·68, 3·90)	1·72 (1·52, 1·94)	37·10 (32·50, 41·93)	3·90 (3·24, 4·64)	2·06 (1·78, 2·37)	41·98 (36·14, 48·76)
Bahamas	Female	2·04 (1·57, 2·49)	1·10 (0·92, 1·30)	23·66 (19·69, 27·74)	2·55 (2·01, 3·12)	1·37 (1·15, 1·63)	27·90 (23·37, 32·88)
Bahamas	Male	4·83 (3·95, 6·02)	2·69 (2·26, 3·20)	55·46 (46·44, 65·86)	5·66 (4·58, 7·05)	3·04 (2·51, 3·73)	60·34 (49·54, 74·10)
**Barbados**	Both	4·32 (3·62, 5·28)	2·31 (2·03, 2·69)	44·95 (39·62, 51·92)	5·39 (4·51, 6·43)	2·69 (2·36, 3·11)	52·36 (45·51, 60·48)
Barbados	Female	2·74 (2·17, 3·40)	1·49 (1·24, 1·79)	28·73 (24·10, 34·43)	3·44 (2·74, 4·36)	1·77 (1·45, 2·13)	34·00 (28·32, 40·61)
Barbados	Male	6·50 (5·26, 8·03)	3·54 (2·92, 4·27)	67·42 (55·91, 81·42)	7·89 (6·29, 9·56)	3·97 (3·26, 4·82)	75·92 (61·66, 91·97)
**Belize**	Both	3·73 (3·16, 4·39)	2·24 (1·98, 2·51)	45·78 (40·32, 51·27)	3·71 (3·03, 4·48)	2·04 (1·75, 2·37)	41·27 (35·10, 47·83)
Belize	Female	2·88 (2·22, 3·62)	1·71 (1·37, 2·03)	35·73 (28·50, 42·40)	3·06 (2·30, 4·15)	1·67 (1·31, 2·12)	34·68 (27·40, 43·58)
Belize	Male	4·58 (3·79, 5·52)	2·80 (2·38, 3·33)	55·73 (47·05, 66·07)	4·38 (3·48, 5·42)	2·44 (1·97, 2·98)	48·05 (38·87, 58·86)
**Bermuda**	Both	12·16 (9·94, 14·85)	5·31 (4·67, 5·96)	101·43 (88·66, 114·23)	12·06 (9·68, 14·45)	4·64 (3·92, 5·29)	89·37 (76·40, 101·90)
Bermuda	Female	5·45 (4·23, 6·91)	2·54 (2·16, 2·98)	45·71 (38·48, 53·58)	5·45 (4·19, 6·87)	2·23 (1·80, 2·64)	41·59 (34·40, 48·81)
Bermuda	Male	21·50 (17·67, 26·79)	9·55 (8·00, 11·08)	179·13 (150·04, 205·99)	20·32 (15·70, 24·80)	7·99 (6·51, 9·62)	149·82 (121·73, 180·12)
**Bolivia (Plurinational State of)**	Both	2·63 (1·92, 3·69)	1·57 (1·13, 2·22)	31·85 (23·22, 44·58)	4·52 (3·10, 6·65)	2·17 (1·54, 3·10)	45·75 (32·60, 64·50)
Bolivia (Plurinational State of)	Female	1·47 (1·00, 2·16)	1·01 (0·66, 1·47)	21·11 (14·34, 30·74)	2·16 (1·30, 3·31)	1·24 (0·78, 1·83)	27·53 (17·47, 41·19)

Location	Sex	2000 Incidence	2000 Mortality	2000 DALYs	2023 Incidence	2023 Mortality	2023 DALYs
Bolivia (Plurinational State of)	Male	4·07 (2·72, 6·42)	2·33 (1·50, 3·67)	45·19 (29·55, 71·13)	7·54 (4·63, 11·99)	3·49 (2·15, 5·40)	69·40 (42·44, 106·64)
**Brazil**	Both	4·30 (3·82, 4·86)	2·47 (2·27, 2·64)	50·74 (47·59, 54·09)	4·66 (4·03, 5·31)	2·50 (2·25, 2·68)	48·98 (45·44, 51·88)
Brazil	Female	2·53 (2·11, 3·11)	1·43 (1·26, 1·61)	30·39 (27·27, 33·71)	2·95 (2·41, 3·50)	1·55 (1·33, 1·68)	31·78 (28·50, 34·37)
Brazil	Male	6·52 (5·85, 7·35)	3·84 (3·57, 4·09)	76·13 (71·31, 81·25)	6·91 (6·09, 7·74)	3·84 (3·50, 4·13)	71·63 (66·52, 76·68)
**Canada**	Both	10·21 (8·87, 11·79)	4·07 (3·77, 4·30)	78·27 (73·38, 82·30)	8·69 (7·35, 10·35)	3·53 (3·18, 3·83)	61·62 (56·13, 66·53)
Canada	Female	5·63 (4·62, 7·07)	1·94 (1·73, 2·11)	36·97 (33·61, 40·37)	4·89 (3·86, 6·04)	1·70 (1·46, 1·88)	30·76 (27·27, 34·49)
Canada	Male	16·47 (14·62, 18·63)	7·32 (6·82, 7·75)	133·67 (125·51, 141·50)	13·36 (11·43, 15·71)	5·99 (5·40, 6·53)	99·51 (90·39, 109·15)
**Chile**	Both	5·90 (5·17, 6·86)	3·00 (2·76, 3·26)	58·95 (55·42, 63·56)	5·23 (4·46, 6·18)	2·32 (2·10, 2·55)	44·70 (41·24, 48·23)
Chile	Female	4·20 (3·53, 5·05)	2·08 (1·84, 2·33)	40·79 (36·89, 45·26)	3·27 (2·54, 3·97)	1·40 (1·20, 1·58)	26·93 (23·82, 29·96)
Chile	Male	8·16 (7·15, 9·42)	4·34 (3·94, 4·77)	82·93 (76·47, 90·63)	7·73 (6·78, 8·83)	3·59 (3·29, 3·98)	67·43 (62·14, 73·70)
**Colombia**	Both	3·03 (2·68, 3·47)	1·64 (1·52, 1·77)	33·12 (31·04, 35·46)	2·44 (2·07, 2·84)	1·15 (1·04, 1·25)	22·75 (20·86, 24·63)
Colombia	Female	2·16 (1·81, 2·56)	1·17 (1·06, 1·31)	23·75 (21·34, 26·49)	1·45 (1·17, 1·78)	0·68 (0·58, 0·76)	13·83 (12·21, 15·35)
Colombia	Male	4·03 (3·57, 4·57)	2·19 (2·01, 2·38)	43·89 (40·44, 47·60)	3·70 (3·21, 4·22)	1·79 (1·62, 1·97)	34·20 (31·14, 37·32)
**Costa Rica**	Both	4·42 (3·73, 5·24)	2·25 (2·00, 2·52)	43·44 (38·57, 48·41)	4·52 (3·69, 5·46)	2·04 (1·77, 2·34)	39·61 (34·36, 45·88)
Costa Rica	Female	2·58 (2·07, 3·33)	1·30 (1·10, 1·51)	25·53 (21·73, 29·62)	2·27 (1·75, 2·86)	1·03 (0·85, 1·22)	20·30 (16·84, 24·37)
Costa Rica	Male	6·49 (5·38, 7·78)	3·39 (2·92, 4·00)	63·74 (54·73, 74·42)	7·07 (5·70, 8·73)	3·28 (2·73, 3·92)	61·80 (51·32, 73·46)
**Cuba**	Both	7·09 (6·20, 8·23)	3·47 (3·22, 3·70)	69·57 (64·55, 74·33)	8·49 (6·97, 10·04)	3·78 (3·33, 4·33)	75·83 (66·56, 85·50)
Cuba	Female	3·59 (2·90, 4·48)	1·72 (1·49, 1·95)	35·56 (30·69, 40·27)	4·42 (3·39, 5·47)	2·02 (1·68, 2·38)	40·34 (33·93, 46·76)
Cuba	Male	10·90 (9·56, 12·52)	5·43 (4·98, 5·88)	106·57 (97·69, 115·17)	13·23 (10·72, 16·06)	5·92 (5·06, 6·93)	117·18 (100·77, 136·32)
**Dominica**	Both	4·04 (3·11, 5·14)	2·49 (1·95, 3·18)	48·56 (38·96, 61·29)	4·89 (3·65, 6·40)	2·91 (2·18, 3·70)	56·82 (43·17, 71·89)
Dominica	Female	3·34 (2·45, 4·62)	2·04 (1·52, 2·79)	40·44 (30·46, 53·86)	3·98 (2·61, 6·11)	2·38 (1·59, 3·43)	47·00 (31·73, 67·72)

Location	Sex	2000 Incidence	2000 Mortality	2000 DALYs	2023 Incidence	2023 Mortality	2023 DALYs
Dominica	Male	4·98<br>(3·51, 7·04)	3·13<br>(2·27, 4·45)	59·37<br>(43·04, 83·47)	5·90<br>(3·86, 8·14)	3·56<br>(2·35, 4·83)	67·69<br>(45·06, 91·95)
**Dominican Republic**	Both	1·79<br>(1·30, 2·25)	1·09<br>(0·81, 1·37)	22·03<br>(16·60, 27·92)	2·85<br>(2·12, 3·83)	1·70<br>(1·31, 2·28)	33·32<br>(25·77, 44·06)
Dominican Republic	Female	1·21<br>(0·78, 1·64)	0·74<br>(0·50, 1·00)	14·96<br>(9·97, 19·70)	1·85<br>(1·14, 2·55)	1·11<br>(0·70, 1·47)	21·98<br>(14·18, 29·08)
Dominican Republic	Male	2·41<br>(1·75, 3·27)	1·46<br>(1·07, 1·97)	29·48<br>(21·78, 40·03)	4·03<br>(2·67, 5·66)	2·44<br>(1·66, 3·50)	46·64<br>(31·71, 65·72)
**Ecuador**	Both	3·49<br>(3·00, 4·09)	1·81<br>(1·64, 2·01)	35·19<br>(32·06, 39·20)	2·67<br>(2·23, 3·18)	1·17<br>(1·04, 1·30)	22·77<br>(20·50, 25·34)
Ecuador	Female	2·09<br>(1·68, 2·59)	1·29<br>(1·10, 1·50)	25·44<br>(21·71, 29·49)	1·46<br>(1·15, 1·84)	0·79<br>(0·67, 0·93)	15·81<br>(13·40, 18·71)
Ecuador	Male	5·02<br>(4·18, 5·88)	2·39<br>(2·11, 2·70)	45·87<br>(40·21, 51·99)	4·08<br>(3·38, 4·94)	1·62<br>(1·43, 1·85)	30·91<br>(27·04, 35·69)
**El Salvador**	Both	1·78<br>(1·37, 2·26)	0·98<br>(0·78, 1·20)	20·87<br>(16·80, 25·33)	2·84<br>(2·18, 3·71)	1·46<br>(1·16, 1·81)	29·78<br>(23·69, 36·77)
El Salvador	Female	1·55<br>(1·09, 2·16)	0·86<br>(0·62, 1·12)	18·25<br>(13·38, 23·67)	2·34<br>(1·63, 3·12)	1·22<br>(0·90, 1·67)	24·81<br>(18·29, 33·60)
El Salvador	Male	2·06<br>(1·44, 2·86)	1·13<br>(0·78, 1·57)	24·05<br>(16·60, 33·18)	3·59<br>(2·53, 5·47)	1·85<br>(1·35, 2·69)	37·28<br>(27·60, 54·89)
**Greenland**	Both	11·52<br>(7·56, 14·64)	7·17<br>(4·86, 9·13)	131·59<br>(88·50, 167·94)	10·27<br>(7·41, 13·68)	6·00<br>(4·45, 7·74)	101·66<br>(75·40, 130·88)
Greenland	Female	5·83<br>(4·25, 8·00)	3·40<br>(2·41, 4·51)	60·89<br>(44·60, 80·63)	5·73<br>(3·63, 8·53)	3·15<br>(2·14, 4·47)	51·50<br>(35·75, 72·67)
Greenland	Male	19·39<br>(12·61, 26·64)	13·00<br>(8·78, 17·86)	225·21<br>(147·55, 305·50)	14·71<br>(10·20, 20·92)	9·03<br>(6·34, 12·38)	149·88<br>(103·14, 207·15)
**Grenada**	Both	3·84<br>(3·25, 4·59)	2·23<br>(1·92, 2·57)	45·78<br>(39·74, 52·83)	3·89<br>(3·20, 4·64)	2·09<br>(1·79, 2·44)	44·24<br>(38·55, 50·98)
Grenada	Female	2·82<br>(2·30, 3·53)	1·66<br>(1·39, 1·96)	33·85<br>(28·72, 40·33)	3·23<br>(2·46, 4·14)	1·78<br>(1·38, 2·19)	37·27<br>(29·57, 45·84)
Grenada	Male	5·13<br>(4·16, 6·35)	3·00<br>(2·48, 3·61)	60·86<br>(50·52, 72·40)	4·57<br>(3·57, 5·69)	2·45<br>(1·92, 2·99)	51·53<br>(40·63, 62·44)
**Guatemala**	Both	1·62<br>(1·38, 1·90)	1·05<br>(0·93, 1·18)	20·75<br>(18·33, 23·33)	1·35<br>(1·13, 1·63)	0·80<br>(0·69, 0·93)	15·66<br>(13·55, 18·09)
Guatemala	Female	1·42<br>(1·11, 1·74)	0·90<br>(0·74, 1·07)	18·38<br>(15·15, 21·55)	1·12<br>(0·87, 1·40)	0·65<br>(0·53, 0·79)	13·25<br>(10·65, 16·07)
Guatemala	Male	1·85<br>(1·53, 2·24)	1·23<br>(1·05, 1·46)	23·42<br>(19·82, 28·05)	1·62<br>(1·31, 2·04)	0·99<br>(0·81, 1·22)	18·55<br>(15·30, 22·74)
**Guyana**	Both	2·52<br>(2·16, 2·98)	1·60<br>(1·40, 1·84)	34·66<br>(30·18, 39·40)	2·91<br>(2·35, 3·54)	1·68<br>(1·40, 2·02)	37·53<br>(31·56, 44·87)
Guyana	Female	1·41<br>(1·17, 1·74)	0·90<br>(0·76, 1·07)	19·59<br>(16·76, 23·37)	1·64<br>(1·23, 2·08)	0·96<br>(0·73, 1·19)	21·52<br>(16·64, 26·69)

Location	Sex	2000 Incidence	2000 Mortality	2000 DALYs	2023 Incidence	2023 Mortality	2023 DALYs
Guyana	Male	3·78<br>(3·09, 4·54)	2·42<br>(2·03, 2·88)	51·59<br>(43·42, 60·59)	4·39<br>(3·51, 5·64)	2·56<br>(2·05, 3·26)	55·98<br>(45·32, 70·51)
**Haiti**	Both	2·66<br>(1·92, 3·69)	1·98<br>(1·39, 2·76)	40·00<br>(28·35, 55·45)	3·62<br>(2·52, 5·00)	2·51<br>(1·75, 3·57)	52·40<br>(36·73, 74·27)
Haiti	Female	1·80<br>(1·15, 2·86)	1·33<br>(0·84, 2·10)	27·42<br>(17·30, 43·27)	2·59<br>(1·43, 4·26)	1·77<br>(1·03, 2·90)	38·25<br>(22·12, 63·62)
Haiti	Male	3·63<br>(2·43, 5·49)	2·73<br>(1·78, 4·09)	54·11<br>(35·95, 81·15)	4·85<br>(2·77, 7·40)	3·39<br>(1·93, 5·32)	69·18<br>(40·32, 107·65)
**Honduras**	Both	0·70<br>(0·51, 0·88)	0·47<br>(0·35, 0·63)	9·29<br>(6·93, 12·18)	0·81<br>(0·57, 1·13)	0·56<br>(0·39, 0·79)	9·77<br>(6·98, 13·51)
Honduras	Female	0·52<br>(0·34, 0·73)	0·33<br>(0·21, 0·46)	7·12<br>(4·67, 9·97)	0·60<br>(0·35, 0·92)	0·38<br>(0·24, 0·58)	7·51<br>(4·88, 11·45)
Honduras	Male	0·89<br>(0·60, 1·29)	0·63<br>(0·42, 0·92)	11·58<br>(7·91, 16·67)	1·03<br>(0·62, 1·64)	0·74<br>(0·43, 1·16)	12·23<br>(7·21, 19·15)
**Jamaica**	Both	4·46<br>(3·71, 5·36)	2·41<br>(2·13, 2·73)	50·10<br>(44·33, 57·00)	5·33<br>(4·38, 6·34)	2·67<br>(2·27, 3·09)	56·62<br>(48·68, 65·91)
Jamaica	Female	3·17<br>(2·51, 4·05)	1·72<br>(1·45, 2·03)	35·84<br>(30·51, 42·11)	3·79<br>(2·94, 4·83)	1·93<br>(1·53, 2·36)	41·24<br>(33·00, 50·66)
Jamaica	Male	5·93<br>(4·77, 7·26)	3·25<br>(2·73, 3·84)	66·31<br>(55·38, 78·84)	7·08<br>(5·66, 8·81)	3·56<br>(2·91, 4·34)	74·09<br>(60·99, 90·26)
**Mexico**	Both	2·59<br>(2·18, 3·01)	1·44<br>(1·29, 1·61)	29·52<br>(26·43, 33·38)	2·32<br>(1·94, 2·77)	1·22<br>(1·08, 1·39)	24·56<br>(21·52, 27·92)
Mexico	Female	1·77<br>(1·44, 2·20)	0·99<br>(0·85, 1·15)	20·46<br>(17·58, 23·91)	1·43<br>(1·16, 1·78)	0·77<br>(0·65, 0·91)	15·41<br>(12·93, 18·16)
Mexico	Male	3·50<br>(2·90, 4·19)	1·95<br>(1·67, 2·26)	39·74<br>(33·60, 46·15)	3·37<br>(2·78, 4·16)	1·76<br>(1·49, 2·09)	35·35<br>(29·86, 41·64)
**Nicaragua**	Both	1·33<br>(1·01, 1·69)	0·74<br>(0·58, 0·95)	15·69<br>(12·14, 19·75)	1·62<br>(1·17, 2·14)	0·82<br>(0·60, 1·08)	16·66<br>(12·43, 21·69)
Nicaragua	Female	1·08<br>(0·78, 1·49)	0·59<br>(0·42, 0·79)	12·85<br>(9·38, 16·84)	1·26<br>(0·80, 1·81)	0·64<br>(0·43, 0·89)	13·19<br>(8·82, 18·56)
Nicaragua	Male	1·64<br>(1·15, 2·19)	0·95<br>(0·67, 1·29)	19·19<br>(13·74, 25·70)	2·11<br>(1·38, 3·11)	1·10<br>(0·71, 1·65)	21·49<br>(13·81, 30·93)
**Panama**	Both	2·54<br>(2·12, 3·04)	1·36<br>(1·19, 1·53)	26·39<br>(23·35, 30·02)	2·13<br>(1·72, 2·58)	0·99<br>(0·84, 1·13)	19·78<br>(16·96, 22·59)
Panama	Female	1·85<br>(1·44, 2·36)	0·96<br>(0·80, 1·15)	19·28<br>(16·29, 22·85)	1·55<br>(1·21, 1·97)	0·71<br>(0·59, 0·85)	14·60<br>(12·33, 17·50)
Panama	Male	3·28<br>(2·69, 3·93)	1·80<br>(1·53, 2·10)	33·99<br>(28·58, 39·88)	2·80<br>(2·21, 3·43)	1·35<br>(1·08, 1·64)	25·75<br>(20·80, 30·79)
**Paraguay**	Both	2·16<br>(1·70, 2·66)	1·34<br>(1·07, 1·64)	26·30<br>(21·69, 32·07)	3·11<br>(2·30, 3·94)	1·85<br>(1·35, 2·30)	35·10<br>(25·56, 43·14)
Paraguay	Female	1·04<br>(0·77, 1·36)	0·63<br>(0·48, 0·81)	12·91<br>(9·83, 16·39)	1·44<br>(1·01, 1·99)	0·84<br>(0·62, 1·13)	16·59<br>(12·15, 22·23)

Location	Sex	2000 Incidence	2000 Mortality	2000 DALYs	2023 Incidence	2023 Mortality	2023 DALYs
Paraguay	Male	3·44 (2·47, 4·58)	2·20 (1·63, 2·86)	41·66 (31·41, 54·03)	5·26 (3·54, 6·87)	3·27 (2·17, 4·22)	58·76 (39·69, 75·53)
**Peru**	Both	2·78 (2·24, 3·34)	1·36 (1·09, 1·62)	27·11 (21·70, 32·38)	3·78 (3·07, 4·62)	1·57 (1·33, 1·85)	29·49 (25·15, 34·99)
Peru	Female	1·66 (1·22, 2·25)	0·98 (0·73, 1·30)	19·94 (15·02, 26·31)	2·11 (1·51, 2·90)	1·11 (0·82, 1·48)	21·39 (16·48, 28·35)
Peru	Male	4·01 (3·16, 4·98)	1·80 (1·43, 2·23)	35·06 (28·16, 42·60)	5·68 (4·56, 6·95)	2·14 (1·76, 2·44)	38·90 (31·74, 47·06)
**Puerto Rico**	Both	5·64 (4·85, 6·59)	2·66 (2·45, 2·97)	53·75 (49·53, 59·43)	5·01 (4·17, 6·01)	2·05 (1·80, 2·28)	41·30 (36·60, 46·12)
Puerto Rico	Female	3·45 (2·78, 4·33)	1·67 (1·44, 1·91)	32·99 (28·75, 37·80)	2·87 (2·25, 3·60)	1·25 (1·05, 1·46)	24·15 (20·23, 28·33)
Puerto Rico	Male	8·35 (7·11, 9·76)	3·93 (3·47, 4·44)	79·48 (70·26, 89·20)	7·55 (6·34, 9·18)	3·06 (2·64, 3·51)	61·72 (53·25, 70·12)
**Saint Kitts and Nevis**	Both	4·73 (3·96, 5·61)	2·91 (2·55, 3·27)	57·06 (49·61, 63·82)	4·85 (4·11, 5·66)	2·81 (2·43, 3·22)	54·69 (48·10, 62·02)
Saint Kitts and Nevis	Female	3·77 (2·93, 4·68)	2·32 (1·93, 2·72)	45·90 (38·54, 53·85)	4·06 (3·19, 4·97)	2·35 (1·96, 2·81)	46·51 (38·90, 55·31)
Saint Kitts and Nevis	Male	5·98 (4·90, 7·34)	3·74 (3·16, 4·41)	71·75 (60·35, 84·30)	5·82 (4·69, 7·03)	3·41 (2·79, 4·13)	64·58 (53·50, 77·88)
**Saint Lucia**	Both	4·74 (3·97, 5·80)	2·64 (2·30, 3·04)	54·71 (47·66, 62·61)	4·64 (3·87, 5·51)	2·40 (2·06, 2·80)	49·98 (43·53, 58·75)
Saint Lucia	Female	3·72 (2·87, 4·66)	2·09 (1·66, 2·52)	43·33 (34·48, 51·74)	3·39 (2·60, 4·33)	1·82 (1·44, 2·27)	37·20 (29·50, 46·08)
Saint Lucia	Male	5·95 (4·90, 7·41)	3·32 (2·81, 4·00)	68·30 (57·98, 82·51)	6·00 (4·74, 7·68)	3·05 (2·46, 3·79)	63·88 (52·20, 78·85)
**Saint Vincent and the Grenadines**	Both	3·54 (3·00, 4·17)	2·08 (1·81, 2·36)	43·17 (37·99, 48·87)	3·47 (2·94, 4·12)	1·85 (1·58, 2·16)	40·25 (33·90, 47·15)
Saint Vincent and the Grenadines	Female	2·09 (1·61, 2·63)	1·22 (0·99, 1·45)	25·74 (20·99, 30·77)	1·98 (1·49, 2·45)	1·08 (0·86, 1·30)	23·51 (18·83, 27·77)
Saint Vincent and the Grenadines	Male	5·31 (4·40, 6·48)	3·19 (2·66, 3·81)	64·29 (53·41, 76·42)	4·90 (3·91, 6·07)	2·63 (2·12, 3·24)	56·24 (45·06, 69·14)
**Suriname**	Both	2·56 (1·88, 3·27)	1·61 (1·23, 2·06)	33·40 (25·58, 42·70)	2·84 (2·04, 3·82)	1·69 (1·26, 2·20)	34·79 (25·52, 45·09)
Suriname	Female	1·32 (0·89, 1·85)	0·84 (0·58, 1·13)	17·39 (12·41, 23·68)	1·57 (1·05, 2·24)	0·94 (0·63, 1·35)	19·51 (13·14, 27·67)
Suriname	Male	3·95 (2·74, 5·37)	2·51 (1·77, 3·42)	51·25 (36·32, 69·43)	4·45 (2·87, 6·50)	2·70 (1·83, 3·92)	53·84 (35·98, 76·98)
**Trinidad and Tobago**	Both	3·66 (3·13, 4·32)	2·28 (2·02, 2·58)	44·22 (38·94, 49·86)	4·39 (3·63, 5·27)	2·44 (2·11, 2·86)	47·49 (40·94, 56·10)
Trinidad and Tobago	Female	2·31 (1·84, 2·76)	1·45 (1·25, 1·70)	28·01 (23·99, 33·12)	2·63 (2·10, 3·45)	1·53 (1·28, 1·90)	29·03 (24·22, 36·15)

Location	Sex	2000 Incidence	2000 Mortality	2000 DALYs	2023 Incidence	2023 Mortality	2023 DALYs
Trinidad and Tobago	Male	5·24 (4·31, 6·31)	3·30 (2·78, 3·88)	62·92 (52·55, 74·07)	6·56 (5·24, 8·07)	3·73 (3·06, 4·61)	70·25 (57·60, 87·24)
**United States of America**	Both	16·04 (13·81, 18·19)	3·57 (3·28, 3·82)	75·89 (70·51, 81·41)	14·18 (12·07, 15·96)	3·35 (2·98, 3·63)	66·04 (60·07, 71·40)
United States of America	Female	7·83 (6·17, 9·78)	1·84 (1·58, 2·07)	38·33 (33·96, 42·55)	6·92 (5·47, 8·49)	1·66 (1·40, 1·91)	33·63 (29·07, 38·38)
United States of America	Male	27·19 (23·69, 30·51)	6·25 (5·78, 6·71)	127·00 (118·11, 138·27)	23·08 (20·11, 25·88)	5·65 (5·04, 6·19)	106·18 (96·99, 116·40)
**Uruguay**	Both	12·09 (10·46, 13·94)	5·96 (5·47, 6·49)	121·80 (111·50, 133·39)	10·98 (9·31, 12·78)	5·05 (4·50, 5·61)	102·44 (91·56, 113·71)
Uruguay	Female	4·65 (3·82, 5·84)	2·35 (2·02, 2·66)	45·05 (39·31, 50·85)	4·79 (3·85, 5·84)	2·13 (1·85, 2·47)	43·03 (37·55, 51·40)
Uruguay	Male	22·26 (19·16, 25·35)	11·39 (10·25, 12·59)	226·15 (203·69, 250·92)	19·75 (16·85, 23·07)	9·72 (8·49, 11·15)	186·12 (163·41, 209·79)
**Venezuela (Bolivarian Republic of)**	Both	3·46 (3·00, 3·97)	2·04 (1·88, 2·21)	39·59 (36·64, 42·89)	4·25 (3·61, 4·97)	2·43 (2·16, 2·72)	46·85 (41·37, 52·90)
Venezuela (Bolivarian Republic of)	Female	2·23 (1·86, 2·67)	1·33 (1·18, 1·50)	25·74 (22·86, 28·61)	2·56 (2·02, 3·34)	1·48 (1·25, 1·88)	28·89 (24·09, 36·27)
Venezuela (Bolivarian Republic of)	Male	5·00 (4·36, 5·77)	3·00 (2·74, 3·32)	56·88 (51·87, 62·87)	6·54 (5·56, 7·73)	3·87 (3·40, 4·43)	71·05 (61·90, 81·04)

Abbreviation: DALYs = disability-adjusted life years.

Table S10. Prostate cancer — annual percentage change (APC) for age-standardised incidence, mortality and DALY rates, 2000–2023 (UI 95 %)

Location	Incidence	Mortality	DALYs
**Global**	-0·51<br>(-0·61, -0·41)	-1·15<br>(-1·26, -1·04)	-1·04<br>(-1·15, -0·92)
**High SDI**	-0·70<br>(-0·81, -0·59)	-1·68<br>(-1·80, -1·57)	-1·58<br>(-1·68, -1·48)
**High-income North America**	-1·42<br>(-1·59, -1·24)	-1·82<br>(-2·05, -1·59)	-1·71<br>(-1·93, -1·48)
**High-middle SDI**	0·25<br>(0·13, 0·38)	-1·21<br>(-1·30, -1·13)	-0·97<br>(-1·07, -0·88)
**Latin America and Caribbean**	0·29<br>(0·18, 0·40)	-0·65<br>(-0·74, -0·55)	-0·63<br>(-0·72, -0·54)
**Middle SDI**	1·35<br>(1·23, 1·47)	0·31<br>(0·22, 0·41)	0·39<br>(0·29, 0·48)
**Low-middle SDI**	1·39<br>(1·24, 1·54)	0·56<br>(0·44, 0·68)	0·50<br>(0·36, 0·65)
**Low SDI**	1·21<br>(0·96, 1·46)	0·56<br>(0·35, 0·78)	0·62<br>(0·40, 0·84)

Abbreviation: DALYs = disability-adjusted life years.

Table S11. Testicular cancer — annual percentage change (APC) for age-standardised incidence, mortality and DALY rates, 2000–2023 (UI 95 %)

Location	Incidence	Mortality	DALYs
**Global**	0·96<br>(0·79, 1·12)	-0·29<br>(-0·54, -0·05)	-0·05<br>(-0·29, 0·19)
**High SDI**	0·83<br>(0·66, 1·01)	-1·12<br>(-1·36, -0·87)	-0·82<br>(-1·08, -0·56)
**High-income North America**	0·80<br>(0·51, 1·10)	0·22<br>(-0·05, 0·50)	0·46<br>(0·14, 0·77)
**High-middle SDI**	3·64<br>(3·28, 4·00)	0·39<br>(0·01, 0·78)	4·58<br>(4·35, 4·91)
**Latin America and Caribbean**	4·26<br>(4·02, 4·49)	2·18<br>(1·93, 2·43)	2·60<br>(2·36, 2·84)
**Middle SDI**	3·32<br>(3·15, 3·50)	0·79<br>(0·67, 0·90)	0·97<br>(0·88, 1·07)
**Low-middle SDI**	2·28<br>(2·12, 2·45)	0·38<br>(0·21, 0·54)	0·35<br>(0·18, 0·51)
**Low SDI**	2·12<br>(1·69, 2·56)	0·30<br>(-0·03, 0·63)	0·28<br>(-0·05, 0·61)

Abbreviation: DALYs = disability-adjusted life years.

Table S12. Kidney cancer — annual percentage change (APC) for age-standardised incidence, mortality and DALY rates, 2000–2023 (UI 95 %)

Location	Incidence	Mortality	DALYs
**Global**	-0·17<br>(-0·33, -0·02)	-0·62<br>(-0·71, -0·53)	-0·81<br>(-0·88, -0·74)
**High SDI**	-0·26<br>(-0·46, -0·06)	-0·83<br>(-0·94, -0·73)	-1·20<br>(-1·31, -1·10)
**High-income North America**	-1·04<br>(-1·20, -0·89)	-0·98<br>(-1·08, -0·89)	-1·31<br>(-1·41, -1·22)
**High-middle SDI**	1·11<br>(0·99, 1·23)	0·10<br>(-0·01, 0·21)	-0·12<br>(-0·22, -0·02)
**Latin America and Caribbean**	1·46<br>(1·34, 1·57)	0·84<br>(0·75, 0·93)	0·61<br>(0·52, 0·70)
**Middle SDI**	1·65<br>(1·56, 1·73)	0·95<br>(0·82, 1·08)	0·69<br>(0·58, 0·80)
**Low-middle SDI**	1·56<br>(1·44, 1·68)	0·90<br>(0·79, 1·01)	0·89<br>(0·78, 1·00)
**Low SDI**	1·54<br>(1·46, 1·62)	0·87<br>(0·78, 0·96)	0·91<br>(0·82, 1·00)

Abbreviation: DALYs = disability-adjusted life years.

Table S13. Bladder cancer — annual percentage change (APC) for age-standardised incidence, mortality and DALY rates, 2000–2023 (UI 95 %)

Location	Incidence	Mortality	DALYs
**Global**	-0·70<br>(-0·77, -0·62)	-0·94<br>(-1·05, -0·82)	-1·17<br>(-1·30, -1·03)
**High SDI**	-0·73<br>(-0·81, -0·66)	-1·03<br>(-1·10, -0·97)	-1·37<br>(-1·44, -1·29)
**High-income North America**	-0·66<br>(-0·83, -0·49)	-0·29<br>(-0·45, -0·14)	-0·70<br>(-0·85, -0·54)
**High-middle SDI**	-0·14<br>(-0·40, 0·13)	-0·97<br>(-1·24, -0·71)	-1·14<br>(-1·42, -0·87)
**Latin America and Caribbean**	0·19<br>(0·13, 0·25)	-0·11<br>(-0·15, -0·06)	-0·24<br>(-0·29, -0·18)
**Middle SDI**	0·35<br>(-0·04, 0·74)	-0·32<br>(-0·66, 0·02)	-0·60<br>(-0·97, -0·22)
**Low-middle SDI**	0·64<br>(0·41, 0·87)	0·11<br>(-0·08, 0·29)	0·13<br>(-0·08, 0·33)
**Low SDI**	0·78<br>(0·45, 1·12)	0·31<br>(0·05, 0·58)	0·36<br>(0·06, 0·66)

Abbreviation: DALYs = disability-adjusted life years.

Table S14. Methods. Estimation of the Average Annual Growth Rate.

Statistical methodology

We analyzed the temporal evolution of an annual population-level rate associated with a disease for the years $t = 2000, \ldots, 2023$. For each year, an estimated value val_t and its corresponding 95% confidence interval ($lower_t$, $upper_t$) were available.

The objective of the analysis was to estimate the *average annual growth rate* (Annual Percent Change, APC) over the study period, while explicitly accounting for differences in the precision of the yearly estimates.

Log-linear trend model

Temporal trends were modeled assuming a smooth exponential pattern using a log-linear regression:

$$\log(val_t) = \alpha + \beta \cdot t + \varepsilon_t, \qquad (1)$$

where α denotes the intercept, β is the temporal trend coefficient, and ε_t is a random error term.

Under this specification, the coefficient β represents the average relative annual change in the rate. The APC is obtained through the transformation:

$$APC = \left(e^{\beta} - 1\right) \times 100. \qquad (2)$$

Incorporation of annual uncertainty

To account for heterogeneity in the precision of the annual estimates, a *weighted least squares* (WLS) approach was employed.

The standard error of each annual estimate was approximated from its reported 95% confidence interval, assuming asymptotic normality:

$$SE(val_t) \approx \frac{upper_t - lower_t}{2 \cdot 1.96}. \qquad (3)$$

Delta method for variance propagation

Because the regression model is specified on the logarithmic scale, the uncertainty of each annual estimate was propagated from the original scale to the log scale using the first-order delta method.

Let $g(x) = \log(x)$. Under standard regularity conditions, the delta method yields the approxi- mation:

$$SE\left(\log(val_t)\right) \approx \left|g'(val_t)\right| \cdot SE(val_t) = \frac{SE(val_t)}{val_t}. \qquad (4)$$

This approximation allows uncertainty derived from the confidence intervals of val_t to be co- herently incorporated into the log-linear model.

Weights were then defined as the inverse of the variance on the log scale:

$$w_t = \frac{1}{SE\left(\log(val_t)\right)^2}. \qquad (5)$$

Thus, years with more precise estimates (narrower confidence intervals) exert greater influence on the estimation of the overall temporal trend.

Estimation and confidence intervals

The weighted log-linear model was fitted using weighted least squares. A 95% confidence interval for the trend coefficient β was obtained from the fitted model. This interval was subsequently transformed to the APC scale using the exponential mapping:

$$CI_{95\%}(APC) = \left(\left(e^{\beta_L} - 1\right) \times 100,\ \left(e^{\beta_U} - 1\right) \times 100\right), \qquad (6)$$

where (β_L, β_U) denotes the 95% confidence interval for β.

Predicted values

Predicted values on the original scale were obtained as:

$$\widehat{val}_t = \exp\left(\widehat{\log(val_t)}\right), \qquad (7)$$

and were used to visually compare observed values with the estimated temporal trend.

Assumptions

This approach relies on the following assumptions:

- The annual estimates val_t are asymptotically normally distributed.
- The reported confidence intervals accurately reflect the uncertainty of val_t.
- The log-linear model adequately captures the long-term temporal trend.
- Estimation errors across years are uncorrelated.
- The delta method provides a valid first-order approximation for variance propagation.

Interpretation

The resulting APC represents the average annual percentage change in the population-level rate over the study period, adjusted for the precision of the individual yearly estimates. A negative APC indicates a sustained decline over time, whereas a positive APC indicates an average increase.

Table S15. Statement of GATHER compliance

Item #	Checklist item	Reported on page #
Objectives and funding		
1	Define the indicator(s), populations (including age, sex, and geographic entities), and time period(s) for which estimates were made.	Page 4.
2	List the funding sources for the work.	Page 7. This research received no specific funding from public, commercial, or not-for-profit sectors. Full data access was available to authors, who take final responsibility for submission. As secondary public data were used, no institutional ethics approval was required.
Data inputs		
For all data inputs from multiple sources that are synthesized as part of study:		
3	Describe how the data were identified and how the data were accessed.	Page 6. Section Input Data Sources Overview
4	Specify the inclusion and exclusion criteria. Identify all ad-hoc exclusions.	Page 5-7. Methods
5	Provide information on all included data sources and their main characteristics.	Page 5-7. Methods
6	Identify and describe any categories of input data that have potentially important biases.	Page 5-7. Methods
For data inputs that contribute to the analysis but were not synthesized as part of study:		
7	Describe and give sources for any other data inputs.	Page 6. Input Data Sources Overview
All data inputs		
8	Provide all data inputs in an extractable file format and relevant metadata.	Page 7. All data are publicly available via GBD 2023 on the Global Health Data Exchange (http://ghdx.healthdata.org).
Data analysis		

Item #	Checklist item	Reported on page #
9	Provide a conceptual overview of the data analysis method.	Page 4. Methods → Data Source and GBD Framework Temporal Trend Analysis Study Outcomes and Metrics
10	Provide a detailed description of all steps of the analysis, including mathematical formulae.	Appendix S1. Estimation of the Average Annual Growth Rate
11	Describe how candidate models were evaluated and how the final model(s) were selected.	Page 5. Mortality estimates were modelled using the Cause of Death Ensemble model (CODEm)
12	Provide results of any evaluation of model performance or sensitivity analysis.	Appendix S1. Estimation of the Average Annual Growth Rate
13	Describe methods for calculating uncertainty of the estimates.	Page 5. Study Outcomes and Metrics
14	State how analytic or statistical source code can be accessed.	Page 6. Input Data Sources Overview
Results and discussion		
15	Provide published estimates in an extractable file format.	Table 1-4
16	Report a quantitative measure of uncertainty of the estimates.	Page 7-9. Results section
17	Interpret results in light of existing evidence.	Page 7-9. Results section
18	Discuss limitations of the estimates.	Page 13

Table S16. Mortality-to-incidence ratios (MIRs) according to region and SDI level, 2023.

**Location**	**Prostate**	**Testicular**	**Kidney**	**Bladder**
Global	0.370	0.120	0.411	0.415
High-income North America	0.169	0.041	0.276	0.248
Latin America and Caribbean	0.420	0.137	0.460	0.517
High SDI	0.276	0.061	0.347	0.356
High-middle SDI	0.454	0.148	0.414	0.459
Middle SDI	0.604	0.227	0.450	0.459
Low-middle SDI	0.662	0.276	0.571	0.609
Low SDI	0.816	0.316	0.615	0.607

Table S17. Interval-Specific Annual Percent Changes (APCs) and 95% Confidence Intervals for Age-Standardized Incidence, Mortality, and DALY Rates of Prostate, Testicular, Kidney, and Bladder Cancers Across Global, Regional, and Socioeconomic Groups in the Americas, 2000–2023.

**Incidence**										
**Cancer**	**Measure**	**Location**	**Period**	**APC**	**APC_LCL**	**APC_UCL**	**p_value**	**R2**	**n_years**	**Method**
Prostate cancer	Incidence	Middle SDI	2000–2004	2.054	1.976	2.132	0.000	0.991	5	WLS
Bladder cancer	Incidence	Middle SDI	2000–2009	-1.820	-2.051	-1.589	0.000	0.808	10	WLS
Kidney cancer	Incidence	Middle SDI	2000–2009	2.056	2.033	2.080	0.000	0.998	10	WLS
Testicular cancer	Incidence	Middle SDI	2000–2009	2.263	2.170	2.356	0.000	0.977	10	WLS
Prostate cancer	Incidence	Middle SDI	2005–2009	1.573	1.501	1.645	0.000	0.987	5	WLS
Prostate cancer	Incidence	Middle SDI	2010–2014	0.445	0.352	0.538	0.000	0.775	5	WLS
Bladder cancer	Incidence	Middle SDI	2010–2019	0.956	0.874	1.039	0.000	0.904	10	WLS
Kidney cancer	Incidence	Middle SDI	2010–2019	1.264	1.208	1.320	0.000	0.973	10	WLS
Testicular cancer	Incidence	Middle SDI	2010–2019	3.593	3.527	3.658	0.000	0.995	10	WLS
Prostate cancer	Incidence	Middle SDI	2015–2019	1.203	1.131	1.275	0.000	0.977	5	WLS
Prostate cancer	Incidence	Middle SDI	2020–2023	1.440	0.913	1.969	0.000	0.596	4	WLS
Bladder cancer	Incidence	Middle SDI	2020–2023	4.087	3.523	4.653	0.000	0.914	4	WLS
Kidney cancer	Incidence	Middle SDI	2020–2023	0.274	-0.098	0.648	0.141	0.096	4	WLS
Testicular cancer	Incidence	Middle SDI	2020–2023	3.388	3.063	3.714	0.000	0.956	4	WLS
Prostate cancer	Incidence	Low-middle SDI	2000–2004	1.413	1.236	1.590	0.000	0.923	5	WLS
Bladder cancer	Incidence	Low-middle SDI	2000–2009	-0.148	-0.229	-0.067	0.001	0.221	10	WLS
Kidney cancer	Incidence	Low-middle SDI	2000–2009	1.960	1.892	2.029	0.000	0.986	10	WLS
Testicular cancer	Incidence	Low-middle SDI	2000–2009	2.175	1.933	2.418	0.000	0.874	10	WLS
Prostate cancer	Incidence	Low-middle SDI	2005–2009	1.509	1.411	1.607	0.000	0.978	5	WLS
Prostate cancer	Incidence	Low-middle SDI	2010–2014	0.330	0.268	0.392	0.000	0.842	5	WLS
Bladder cancer	Incidence	Low-middle SDI	2010–2019	0.749	0.612	0.886	0.000	0.717	10	WLS
Kidney cancer	Incidence	Low-middle SDI	2010–2019	1.188	1.124	1.252	0.000	0.967	10	WLS
Testicular cancer	Incidence	Low-middle SDI	2010–2019	1.688	1.644	1.731	0.000	0.992	10	WLS
Prostate cancer	Incidence	Low-middle SDI	2015–2019	1.937	1.816	2.058	0.000	0.980	5	WLS
Prostate cancer	Incidence	Low-middle SDI	2020–2023	0.942	0.251	1.638	0.010	0.314	4	WLS
Bladder cancer	Incidence	Low-middle SDI	2020–2023	1.830	1.148	2.518	0.000	0.641	4	WLS
Kidney cancer	Incidence	Low-middle SDI	2020–2023	-0.395	-1.445	0.667	0.443	0.033	4	WLS
Testicular cancer	Incidence	Low-middle SDI	2020–2023	2.677	0.989	4.393	0.004	0.384	4	WLS
Prostate cancer	Incidence	Low SDI	2000–2004	0.459	0.300	0.619	0.000	0.672	5	WLS
Bladder cancer	Incidence	Low SDI	2000–2009	-0.678	-0.779	-0.577	0.000	0.827	10	WLS
Kidney cancer	Incidence	Low SDI	2000–2009	1.469	1.381	1.558	0.000	0.968	10	WLS
Testicular cancer	Incidence	Low SDI	2000–2009	-0.106	-0.456	0.246	0.546	0.010	10	WLS
Prostate cancer	Incidence	Low SDI	2005–2009	0.783	0.696	0.869	0.000	0.953	5	WLS
Prostate cancer	Incidence	Low SDI	2010–2014	0.637	0.595	0.680	0.000	0.982	5	WLS
Bladder cancer	Incidence	Low SDI	2010–2019	1.150	1.054	1.246	0.000	0.940	10	WLS
Kidney cancer	Incidence	Low SDI	2010–2019	1.709	1.625	1.794	0.000	0.978	10	WLS
Testicular cancer	Incidence	Low SDI	2010–2019	2.472	2.371	2.574	0.000	0.985	10	WLS
Prostate cancer	Incidence	Low SDI	2015–2019	1.259	1.182	1.336	0.000	0.985	5	WLS
Prostate cancer	Incidence	Low SDI	2020–2023	3.266	2.191	4.353	0.000	0.756	4	WLS
Bladder cancer	Incidence	Low SDI	2020–2023	3.936	3.017	4.863	0.000	0.861	4	WLS

Kidney cancer	Incidence	Low SDI	2020–2023	-0.537	-0.986	-0.087	0.023	0.318	4	WLS
Testicular cancer	Incidence	Low SDI	2020–2023	6.369	5.223	7.528	0.000	0.914	4	WLS
Prostate cancer	Incidence	Latin America and Caribbean	2000–2004	1.325	1.249	1.400	0.000	0.991	5	WLS
Bladder cancer	Incidence	Latin America and Caribbean	2000–2009	0.316	0.226	0.407	0.000	0.647	10	WLS
Kidney cancer	Incidence	Latin America and Caribbean	2000–2009	1.639	1.580	1.698	0.000	0.992	10	WLS
Testicular cancer	Incidence	Latin America and Caribbean	2000–2009	2.975	2.917	3.032	0.000	0.998	10	WLS
Prostate cancer	Incidence	Latin America and Caribbean	2005–2009	0.283	0.075	0.492	0.011	0.400	5	WLS
Prostate cancer	Incidence	Latin America and Caribbean	2010–2014	-0.488	-0.626	-0.349	0.000	0.816	5	WLS
Bladder cancer	Incidence	Latin America and Caribbean	2010–2019	0.048	-0.056	0.153	0.350	0.031	10	WLS
Kidney cancer	Incidence	Latin America and Caribbean	2010–2019	1.285	1.169	1.401	0.000	0.949	10	WLS
Testicular cancer	Incidence	Latin America and Caribbean	2010–2019	4.970	4.741	5.199	0.000	0.987	10	WLS
Prostate cancer	Incidence	Latin America and Caribbean	2015–2019	0.405	0.246	0.564	0.000	0.701	5	WLS
Prostate cancer	Incidence	Latin America and Caribbean	2020–2023	0.018	-0.680	0.721	0.955	0.000	4	WLS
Bladder cancer	Incidence	Latin America and Caribbean	2020–2023	-0.052	-0.713	0.613	0.864	0.003	4	WLS
Kidney cancer	Incidence	Latin America and Caribbean	2020–2023	3.999	3.240	4.763	0.000	0.934	4	WLS
Testicular cancer	Incidence	Latin America and Caribbean	2020–2023	4.662	3.705	5.627	0.000	0.924	4	WLS
Prostate cancer	Incidence	High-middle SDI	2000–2004	0.462	0.402	0.521	0.000	0.884	5	WLS
Bladder cancer	Incidence	High-middle SDI	2000–2009	-1.189	-1.351	-1.028	0.000	0.758	10	WLS
Kidney cancer	Incidence	High-middle SDI	2000–2009	1.786	1.729	1.844	0.000	0.983	10	WLS
Testicular cancer	Incidence	High-middle SDI	2000–2009	1.572	1.469	1.675	0.000	0.933	10	WLS
Prostate cancer	Incidence	High-middle SDI	2005–2009	0.253	0.121	0.386	0.000	0.316	5	WLS
Prostate cancer	Incidence	High-middle SDI	2010–2014	-0.775	-0.827	-0.724	0.000	0.966	5	WLS
Bladder cancer	Incidence	High-middle SDI	2010–2019	0.340	0.166	0.513	0.000	0.184	10	WLS
Kidney cancer	Incidence	High-middle SDI	2010–2019	0.550	0.473	0.628	0.000	0.748	10	WLS
Testicular cancer	Incidence	High-middle SDI	2010–2019	4.456	4.333	4.579	0.000	0.988	10	WLS
Prostate cancer	Incidence	High-middle SDI	2015–2019	0.567	0.430	0.704	0.000	0.683	5	WLS
Prostate cancer	Incidence	High-middle SDI	2020–2023	1.248	0.769	1.730	0.000	0.526	4	WLS
Bladder cancer	Incidence	High-middle SDI	2020–2023	2.749	2.009	3.494	0.000	0.696	4	WLS
Kidney cancer	Incidence	High-middle SDI	2020–2023	2.437	2.191	2.685	0.000	0.942	4	WLS
Testicular cancer	Incidence	High-middle SDI	2020–2023	5.224	4.447	6.006	0.000	0.885	4	WLS
Prostate cancer	Incidence	High-income North America	2000–2004	-2.303	-2.671	-1.934	0.000	0.962	5	WLS
Bladder cancer	Incidence	High-income North America	2000–2009	0.112	0.006	0.219	0.040	0.215	10	WLS
Kidney cancer	Incidence	High-income North America	2000–2009	-0.220	-0.351	-0.090	0.002	0.411	10	WLS
Testicular cancer	Incidence	High-income North America	2000–2009	0.015	-0.303	0.334	0.921	0.001	10	WLS
Prostate cancer	Incidence	High-income North America	2005–2009	-1.698	-1.997	-1.398	0.000	0.954	5	WLS
Prostate cancer	Incidence	High-income North America	2010–2014	-2.141	-2.565	-1.716	0.000	0.943	5	WLS
Bladder cancer	Incidence	High-income North America	2010–2019	-1.388	-1.590	-1.186	0.000	0.919	10	WLS
Kidney cancer	Incidence	High-income North America	2010–2019	-1.816	-1.992	-1.638	0.000	0.962	10	WLS
Testicular cancer	Incidence	High-income North America	2010–2019	0.507	0.005	1.012	0.048	0.200	10	WLS
Prostate cancer	Incidence	High-income North America	2015–2019	-0.863	-0.963	-0.762	0.000	0.980	5	WLS
Prostate cancer	Incidence	High-income North America	2020–2023	-0.158	-0.478	0.162	0.272	0.196	4	WLS
Bladder cancer	Incidence	High-income North America	2020–2023	-0.383	-0.749	-0.016	0.044	0.520	4	WLS
Kidney cancer	Incidence	High-income North America	2020–2023	-1.573	-2.235	-0.906	0.001	0.846	4	WLS
Testicular cancer	Incidence	High-income North America	2020–2023	-0.492	-2.232	1.279	0.520	0.072	4	WLS
Prostate cancer	Incidence	High SDI	2000–2004	0.139	0.046	0.232	0.004	0.195	5	WLS
Bladder cancer	Incidence	High SDI	2000–2009	-0.414	-0.451	-0.377	0.000	0.865	10	WLS
Kidney cancer	Incidence	High SDI	2000–2009	0.887	0.832	0.942	0.000	0.930	10	WLS
Testicular cancer	Incidence	High SDI	2000–2009	0.981	0.930	1.031	0.000	0.950	10	WLS

Prostate cancer	Incidence	High SDI	2005–2009	-0.378	-0.495	-0.261	0.000	0.529	5	WLS
Prostate cancer	Incidence	High SDI	2010–2014	-1.744	-1.808	-1.680	0.000	0.987	5	WLS
Bladder cancer	Incidence	High SDI	2010–2019	-0.959	-1.014	-0.904	0.000	0.939	10	WLS
Kidney cancer	Incidence	High SDI	2010–2019	-0.961	-1.010	-0.912	0.000	0.951	10	WLS
Testicular cancer	Incidence	High SDI	2010–2019	0.501	0.389	0.613	0.000	0.505	10	WLS
Prostate cancer	Incidence	High SDI	2015–2019	-0.848	-0.865	-0.831	0.000	0.996	5	WLS
Prostate cancer	Incidence	High SDI	2020–2023	0.187	0.009	0.365	0.040	0.133	4	WLS
Bladder cancer	Incidence	High SDI	2020–2023	0.473	0.240	0.707	0.000	0.364	4	WLS
Kidney cancer	Incidence	High SDI	2020–2023	-0.288	-0.424	-0.152	0.000	0.383	4	WLS
Testicular cancer	Incidence	High SDI	2020–2023	2.748	2.330	3.169	0.000	0.860	4	WLS
Prostate cancer	Incidence	Global	2000–2004	0.039	-0.457	0.536	0.821	0.020	5	WLS
Bladder cancer	Incidence	Global	2000–2009	-0.758	-0.886	-0.629	0.000	0.958	10	WLS
Kidney cancer	Incidence	Global	2000–2009	0.762	0.604	0.919	0.000	0.940	10	WLS
Testicular cancer	Incidence	Global	2000–2009	0.687	0.512	0.862	0.000	0.912	10	WLS
Prostate cancer	Incidence	Global	2005–2009	-0.311	-0.902	0.283	0.194	0.481	5	WLS
Prostate cancer	Incidence	Global	2010–2014	-1.511	-1.760	-1.262	0.000	0.992	5	WLS
Bladder cancer	Incidence	Global	2010–2019	-0.775	-0.975	-0.575	0.000	0.908	10	WLS
Kidney cancer	Incidence	Global	2010–2019	-0.780	-0.890	-0.671	0.000	0.971	10	WLS
Testicular cancer	Incidence	Global	2010–2019	0.814	0.484	1.144	0.000	0.803	10	WLS
Prostate cancer	Incidence	Global	2015–2019	-0.534	-0.659	-0.409	0.001	0.984	5	WLS
Prostate cancer	Incidence	Global	2020–2023	0.440	-0.197	1.082	0.097	0.815	4	WLS
Bladder cancer	Incidence	Global	2020–2023	0.937	-0.934	2.843	0.165	0.697	4	WLS
Kidney cancer	Incidence	Global	2020–2023	-0.274	-1.196	0.658	0.332	0.446	4	WLS
Testicular cancer	Incidence	Global	2020–2023	2.598	-0.743	6.051	0.079	0.847	4	WLS
**Deaths**										
**Cancer**	**Measure**	**Location**	**Period**	**APC**	**APC_LCL**	**APC_UCL**	**p_value**	**R2**	**n_years**	**Method**
Prostate cancer	Deaths	Middle SDI	2000–2004	1.355	1.278	1.432	0.000	0.979	5	WLS
Bladder cancer	Deaths	Middle SDI	2000–2009	-2.246	-2.436	-2.056	0.000	0.904	10	WLS
Kidney cancer	Deaths	Middle SDI	2000–2009	1.659	1.612	1.705	0.000	0.989	10	WLS
Testicular cancer	Deaths	Middle SDI	2000–2009	0.193	0.141	0.245	0.000	0.490	10	WLS
Prostate cancer	Deaths	Middle SDI	2005–2009	0.515	0.424	0.605	0.000	0.830	5	WLS
Prostate cancer	Deaths	Middle SDI	2010–2014	-0.384	-0.466	-0.302	0.000	0.766	5	WLS
Bladder cancer	Deaths	Middle SDI	2010–2019	0.310	0.215	0.405	0.000	0.423	10	WLS
Kidney cancer	Deaths	Middle SDI	2010–2019	0.659	0.616	0.703	0.000	0.941	10	WLS
Testicular cancer	Deaths	Middle SDI	2010–2019	1.071	1.014	1.127	0.000	0.962	10	WLS
Prostate cancer	Deaths	Middle SDI	2015–2019	-0.087	-0.192	0.019	0.104	0.091	5	WLS
Prostate cancer	Deaths	Middle SDI	2020–2023	0.171	-0.386	0.731	0.531	0.018	4	WLS
Bladder cancer	Deaths	Middle SDI	2020–2023	3.168	2.593	3.746	0.000	0.859	4	WLS
Kidney cancer	Deaths	Middle SDI	2020–2023	-0.798	-1.125	-0.469	0.000	0.534	4	WLS
Testicular cancer	Deaths	Middle SDI	2020–2023	0.181	-0.313	0.677	0.456	0.025	4	WLS
Prostate cancer	Deaths	Low-middle SDI	2000–2004	0.658	0.511	0.806	0.000	0.789	5	WLS
Bladder cancer	Deaths	Low-middle SDI	2000–2009	-0.496	-0.567	-0.424	0.000	0.801	10	WLS
Kidney cancer	Deaths	Low-middle SDI	2000–2009	1.361	1.301	1.422	0.000	0.978	10	WLS
Testicular cancer	Deaths	Low-middle SDI	2000–2009	0.249	0.009	0.489	0.042	0.083	10	WLS
Prostate cancer	Deaths	Low-middle SDI	2005–2009	0.336	0.239	0.432	0.000	0.692	5	WLS
Prostate cancer	Deaths	Low-middle SDI	2010–2014	-0.274	-0.320	-0.229	0.000	0.871	5	WLS

Bladder cancer	Deaths	Low-middle SDI	2010–2019	0.282	0.158	0.406	0.000	0.305	10	WLS
Kidney cancer	Deaths	Low-middle SDI	2010–2019	0.672	0.598	0.747	0.000	0.872	10	WLS
Testicular cancer	Deaths	Low-middle SDI	2010–2019	-0.086	-0.166	-0.007	0.034	0.091	10	WLS
Prostate cancer	Deaths	Low-middle SDI	2015–2019	0.729	0.639	0.820	0.000	0.924	5	WLS
Prostate cancer	Deaths	Low-middle SDI	2020–2023	-0.082	-0.589	0.427	0.737	0.006	4	WLS
Bladder cancer	Deaths	Low-middle SDI	2020–2023	1.030	0.644	1.418	0.000	0.637	4	WLS
Kidney cancer	Deaths	Low-middle SDI	2020–2023	-0.862	-1.765	0.050	0.063	0.180	4	WLS
Testicular cancer	Deaths	Low-middle SDI	2020–2023	0.139	-1.421	1.723	0.855	0.002	4	WLS
Prostate cancer	Deaths	Low SDI	2000–2004	0.027	-0.134	0.188	0.733	0.007	5	WLS
Bladder cancer	Deaths	Low SDI	2000–2009	-0.803	-0.885	-0.720	0.000	0.910	10	WLS
Kidney cancer	Deaths	Low SDI	2000–2009	0.961	0.894	1.029	0.000	0.957	10	WLS
Testicular cancer	Deaths	Low SDI	2000–2009	-1.346	-1.643	-1.048	0.000	0.686	10	WLS
Prostate cancer	Deaths	Low SDI	2005–2009	0.181	0.080	0.283	0.001	0.440	5	WLS
Prostate cancer	Deaths	Low SDI	2010–2014	0.138	0.081	0.194	0.000	0.594	5	WLS
Bladder cancer	Deaths	Low SDI	2010–2019	0.555	0.486	0.624	0.000	0.875	10	WLS
Kidney cancer	Deaths	Low SDI	2010–2019	1.067	0.979	1.156	0.000	0.941	10	WLS
Testicular cancer	Deaths	Low SDI	2010–2019	0.655	0.575	0.735	0.000	0.879	10	WLS
Prostate cancer	Deaths	Low SDI	2015–2019	0.379	0.315	0.442	0.000	0.898	5	WLS
Prostate cancer	Deaths	Low SDI	2020–2023	1.599	0.472	2.739	0.009	0.399	4	WLS
Bladder cancer	Deaths	Low SDI	2020–2023	2.686	1.830	3.550	0.000	0.767	4	WLS
Kidney cancer	Deaths	Low SDI	2020–2023	-1.255	-1.547	-0.962	0.000	0.856	4	WLS
Testicular cancer	Deaths	Low SDI	2020–2023	2.883	1.828	3.949	0.000	0.714	4	WLS
Prostate cancer	Deaths	Latin America and Caribbean	2000–2004	0.341	0.280	0.403	0.000	0.918	5	WLS
Bladder cancer	Deaths	Latin America and Caribbean	2000–2009	0.019	-0.070	0.109	0.660	0.007	10	WLS
Kidney cancer	Deaths	Latin America and Caribbean	2000–2009	0.867	0.820	0.915	0.000	0.980	10	WLS
Testicular cancer	Deaths	Latin America and Caribbean	2000–2009	0.955	0.884	1.025	0.000	0.965	10	WLS
Prostate cancer	Deaths	Latin America and Caribbean	2005–2009	-0.428	-0.594	-0.263	0.000	0.706	5	WLS
Prostate cancer	Deaths	Latin America and Caribbean	2010–2014	-1.444	-1.480	-1.408	0.000	0.998	5	WLS
Bladder cancer	Deaths	Latin America and Caribbean	2010–2019	-0.152	-0.247	-0.056	0.003	0.273	10	WLS
Kidney cancer	Deaths	Latin America and Caribbean	2010–2019	0.997	0.868	1.126	0.000	0.901	10	WLS
Testicular cancer	Deaths	Latin America and Caribbean	2010–2019	3.097	2.849	3.345	0.000	0.960	10	WLS
Prostate cancer	Deaths	Latin America and Caribbean	2015–2019	-0.847	-0.992	-0.701	0.000	0.924	5	WLS
Prostate cancer	Deaths	Latin America and Caribbean	2020–2023	-1.021	-1.503	-0.537	0.001	0.687	4	WLS
Bladder cancer	Deaths	Latin America and Caribbean	2020–2023	-0.193	-0.651	0.268	0.373	0.080	4	WLS
Kidney cancer	Deaths	Latin America and Caribbean	2020–2023	2.523	1.967	3.082	0.000	0.912	4	WLS
Testicular cancer	Deaths	Latin America and Caribbean	2020–2023	0.650	-0.204	1.512	0.121	0.223	4	WLS
Prostate cancer	Deaths	High-middle SDI	2000–2004	-0.960	-1.008	-0.911	0.000	0.980	5	WLS
Bladder cancer	Deaths	High-middle SDI	2000–2009	-1.962	-2.110	-1.814	0.000	0.910	10	WLS
Kidney cancer	Deaths	High-middle SDI	2000–2009	0.686	0.598	0.775	0.000	0.779	10	WLS
Testicular cancer	Deaths	High-middle SDI	2000–2009	-1.551	-1.685	-1.416	0.000	0.885	10	WLS
Prostate cancer	Deaths	High-middle SDI	2005–2009	-0.956	-1.072	-0.839	0.000	0.893	5	WLS
Prostate cancer	Deaths	High-middle SDI	2010–2014	-2.179	-2.219	-2.139	0.000	0.997	5	WLS
Bladder cancer	Deaths	High-middle SDI	2010–2019	-0.375	-0.555	-0.194	0.000	0.201	10	WLS
Kidney cancer	Deaths	High-middle SDI	2010–2019	-0.236	-0.318	-0.153	0.000	0.323	10	WLS
Testicular cancer	Deaths	High-middle SDI	2010–2019	1.346	1.199	1.492	0.000	0.834	10	WLS
Prostate cancer	Deaths	High-middle SDI	2015–2019	-1.378	-1.581	-1.175	0.000	0.851	5	WLS
Prostate cancer	Deaths	High-middle SDI	2020–2023	-0.240	-0.558	0.079	0.134	0.084	4	WLS
Bladder cancer	Deaths	High-middle SDI	2020–2023	1.966	1.337	2.599	0.000	0.617	4	WLS

Kidney cancer	Deaths	High-middle SDI	2020–2023	1.281	0.917	1.645	0.000	0.671	4	WLS
Testicular cancer	Deaths	High-middle SDI	2020–2023	1.531	0.723	2.345	0.001	0.370	4	WLS
Prostate cancer	Deaths	High-income North America	2000–2004	-3.296	-3.544	-3.047	0.000	0.991	5	WLS
Bladder cancer	Deaths	High-income North America	2000–2009	0.250	0.170	0.331	0.000	0.702	10	WLS
Kidney cancer	Deaths	High-income North America	2000–2009	-0.673	-0.786	-0.561	0.000	0.897	10	WLS
Testicular cancer	Deaths	High-income North America	2000–2009	-1.002	-1.346	-0.658	0.000	0.673	10	WLS
Prostate cancer	Deaths	High-income North America	2005–2009	-2.428	-2.732	-2.123	0.000	0.976	5	WLS
Prostate cancer	Deaths	High-income North America	2010–2014	-2.313	-2.790	-1.834	0.000	0.938	5	WLS
Bladder cancer	Deaths	High-income North America	2010–2019	-0.748	-0.950	-0.546	0.000	0.769	10	WLS
Kidney cancer	Deaths	High-income North America	2010–2019	-1.271	-1.450	-1.092	0.000	0.924	10	WLS
Testicular cancer	Deaths	High-income North America	2010–2019	0.431	0.023	0.840	0.040	0.215	10	WLS
Prostate cancer	Deaths	High-income North America	2015–2019	-0.765	-0.874	-0.655	0.000	0.970	5	WLS
Prostate cancer	Deaths	High-income North America	2020–2023	-0.819	-1.213	-0.424	0.002	0.810	4	WLS
Bladder cancer	Deaths	High-income North America	2020–2023	-0.473	-0.965	0.022	0.058	0.477	4	WLS
Kidney cancer	Deaths	High-income North America	2020–2023	-1.880	-2.443	-1.314	0.000	0.916	4	WLS
Testicular cancer	Deaths	High-income North America	2020–2023	-1.193	-2.458	0.089	0.063	0.464	4	WLS
Prostate cancer	Deaths	High SDI	2000–2004	-1.670	-1.797	-1.544	0.000	0.949	5	WLS
Bladder cancer	Deaths	High SDI	2000–2009	-1.190	-1.219	-1.161	0.000	0.988	10	WLS
Kidney cancer	Deaths	High SDI	2000–2009	-0.304	-0.369	-0.239	0.000	0.524	10	WLS
Testicular cancer	Deaths	High SDI	2000–2009	-2.144	-2.186	-2.103	0.000	0.992	10	WLS
Prostate cancer	Deaths	High SDI	2005–2009	-1.968	-2.043	-1.893	0.000	0.987	5	WLS
Prostate cancer	Deaths	High SDI	2010–2014	-2.296	-2.345	-2.247	0.000	0.996	5	WLS
Bladder cancer	Deaths	High SDI	2010–2019	-0.919	-0.979	-0.858	0.000	0.920	10	WLS
Kidney cancer	Deaths	High SDI	2010–2019	-1.110	-1.149	-1.071	0.000	0.976	10	WLS
Testicular cancer	Deaths	High SDI	2010–2019	-0.529	-0.628	-0.430	0.000	0.590	10	WLS
Prostate cancer	Deaths	High SDI	2015–2019	-1.206	-1.263	-1.149	0.000	0.979	5	WLS
Prostate cancer	Deaths	High SDI	2020–2023	-0.390	-0.589	-0.190	0.000	0.346	4	WLS
Bladder cancer	Deaths	High SDI	2020–2023	0.234	0.021	0.447	0.032	0.144	4	WLS
Kidney cancer	Deaths	High SDI	2020–2023	-0.647	-0.806	-0.487	0.000	0.694	4	WLS
Testicular cancer	Deaths	High SDI	2020–2023	0.758	0.263	1.255	0.004	0.247	4	WLS
Prostate cancer	Deaths	Global	2000–2004	-1.253	-1.829	-0.674	0.006	0.940	5	WLS
Bladder cancer	Deaths	Global	2000–2009	-1.397	-1.551	-1.242	0.000	0.982	10	WLS
Kidney cancer	Deaths	Global	2000–2009	-0.153	-0.347	0.041	0.107	0.292	10	WLS
Testicular cancer	Deaths	Global	2000–2009	-1.503	-1.762	-1.243	0.000	0.956	10	WLS
Prostate cancer	Deaths	Global	2005–2009	-1.355	-1.709	-1.000	0.001	0.980	5	WLS
Prostate cancer	Deaths	Global	2010–2014	-1.803	-1.950	-1.657	0.000	0.998	5	WLS
Bladder cancer	Deaths	Global	2010–2019	-0.728	-0.984	-0.471	0.000	0.841	10	WLS
Kidney cancer	Deaths	Global	2010–2019	-0.866	-0.960	-0.772	0.000	0.982	10	WLS
Testicular cancer	Deaths	Global	2010–2019	0.189	-0.117	0.496	0.192	0.202	10	WLS
Prostate cancer	Deaths	Global	2015–2019	-0.882	-1.316	-0.446	0.008	0.932	5	WLS
Prostate cancer	Deaths	Global	2020–2023	-0.095	-0.923	0.740	0.672	0.108	4	WLS
Bladder cancer	Deaths	Global	2020–2023	0.718	-1.027	2.493	0.220	0.608	4	WLS
Kidney cancer	Deaths	Global	2020–2023	-0.602	-1.575	0.381	0.118	0.777	4	WLS
Testicular cancer	Deaths	Global	2020–2023	1.061	-2.878	5.160	0.372	0.395	4	WLS
**DALYs**										
**Cancer**	**Measure**	**Location**	**Period**	**APC**	**APC_LCL**	**APC_UCL**	**p_value**	**R2**	**n_years**	**Method**

Prostate cancer	DALYs	Middle SDI	2000–2004	1.265	1.189	1.342	0.000	0.976	5	WLS
Bladder cancer	DALYs	Middle SDI	2000–2009	-2.689	-2.932	-2.445	0.000	0.892	10	WLS
Kidney cancer	DALYs	Middle SDI	2000–2009	1.276	1.226	1.326	0.000	0.978	10	WLS
Testicular cancer	DALYs	Middle SDI	2000–2009	0.400	0.330	0.470	0.000	0.695	10	WLS
Prostate cancer	DALYs	Middle SDI	2005–2009	0.707	0.638	0.777	0.000	0.940	5	WLS
Prostate cancer	DALYs	Middle SDI	2010–2014	-0.357	-0.439	-0.275	0.000	0.741	5	WLS
Bladder cancer	DALYs	Middle SDI	2010–2019	0.014	-0.070	0.098	0.745	0.002	10	WLS
Kidney cancer	DALYs	Middle SDI	2010–2019	0.358	0.315	0.402	0.000	0.825	10	WLS
Testicular cancer	DALYs	Middle SDI	2010–2019	1.220	1.170	1.270	0.000	0.976	10	WLS
Prostate cancer	DALYs	Middle SDI	2015–2019	-0.035	-0.133	0.064	0.478	0.018	5	WLS
Prostate cancer	DALYs	Middle SDI	2020–2023	0.172	-0.446	0.794	0.570	0.015	4	WLS
Bladder cancer	DALYs	Middle SDI	2020–2023	2.979	2.457	3.503	0.000	0.867	4	WLS
Kidney cancer	DALYs	Middle SDI	2020–2023	-1.006	-1.394	-0.617	0.000	0.565	4	WLS
Testicular cancer	DALYs	Middle SDI	2020–2023	0.273	-0.098	0.646	0.142	0.095	4	WLS
Prostate cancer	DALYs	Low-middle SDI	2000–2004	0.438	0.277	0.600	0.000	0.578	5	WLS
Bladder cancer	DALYs	Low-middle SDI	2000–2009	-0.571	-0.667	-0.475	0.000	0.747	10	WLS
Kidney cancer	DALYs	Low-middle SDI	2000–2009	1.134	1.071	1.198	0.000	0.964	10	WLS
Testicular cancer	DALYs	Low-middle SDI	2000–2009	0.338	0.090	0.586	0.009	0.135	10	WLS
Prostate cancer	DALYs	Low-middle SDI	2005–2009	0.386	0.299	0.472	0.000	0.787	5	WLS
Prostate cancer	DALYs	Low-middle SDI	2010–2014	-0.490	-0.547	-0.433	0.000	0.932	5	WLS
Bladder cancer	DALYs	Low-middle SDI	2010–2019	0.228	0.100	0.357	0.001	0.211	10	WLS
Kidney cancer	DALYs	Low-middle SDI	2010–2019	0.654	0.584	0.724	0.000	0.882	10	WLS
Testicular cancer	DALYs	Low-middle SDI	2010–2019	-0.202	-0.273	-0.131	0.000	0.405	10	WLS
Prostate cancer	DALYs	Low-middle SDI	2015–2019	0.924	0.848	1.000	0.000	0.965	5	WLS
Prostate cancer	DALYs	Low-middle SDI	2020–2023	0.229	-0.438	0.901	0.481	0.028	4	WLS
Bladder cancer	DALYs	Low-middle SDI	2020–2023	1.183	0.486	1.885	0.002	0.415	4	WLS
Kidney cancer	DALYs	Low-middle SDI	2020–2023	-1.181	-2.263	-0.086	0.036	0.222	4	WLS
Testicular cancer	DALYs	Low-middle SDI	2020–2023	0.302	-1.284	1.915	0.695	0.009	4	WLS
Prostate cancer	DALYs	Low SDI	2000–2004	0.016	-0.142	0.174	0.835	0.002	5	WLS
Bladder cancer	DALYs	Low SDI	2000–2009	-0.926	-1.023	-0.828	0.000	0.905	10	WLS
Kidney cancer	DALYs	Low SDI	2000–2009	0.822	0.740	0.903	0.000	0.917	10	WLS
Testicular cancer	DALYs	Low SDI	2000–2009	-1.411	-1.725	-1.096	0.000	0.681	10	WLS
Prostate cancer	DALYs	Low SDI	2005–2009	0.247	0.161	0.334	0.000	0.667	5	WLS
Prostate cancer	DALYs	Low SDI	2010–2014	0.152	0.111	0.193	0.000	0.768	5	WLS
Bladder cancer	DALYs	Low SDI	2010–2019	0.681	0.593	0.768	0.000	0.867	10	WLS
Kidney cancer	DALYs	Low SDI	2010–2019	1.143	1.072	1.214	0.000	0.966	10	WLS
Testicular cancer	DALYs	Low SDI	2010–2019	0.659	0.580	0.737	0.000	0.884	10	WLS
Prostate cancer	DALYs	Low SDI	2015–2019	0.642	0.574	0.710	0.000	0.956	5	WLS
Prostate cancer	DALYs	Low SDI	2020–2023	2.419	1.427	3.420	0.000	0.665	4	WLS
Bladder cancer	DALYs	Low SDI	2020–2023	3.301	2.383	4.227	0.000	0.813	4	WLS
Kidney cancer	DALYs	Low SDI	2020–2023	-1.452	-1.953	-0.949	0.000	0.730	4	WLS
Testicular cancer	DALYs	Low SDI	2020–2023	3.202	2.312	4.100	0.000	0.813	4	WLS
Prostate cancer	DALYs	Latin America and Caribbean	2000–2004	0.315	0.247	0.382	0.000	0.887	5	WLS
Bladder cancer	DALYs	Latin America and Caribbean	2000–2009	-0.098	-0.192	-0.004	0.042	0.140	10	WLS
Kidney cancer	DALYs	Latin America and Caribbean	2000–2009	0.575	0.522	0.629	0.000	0.946	10	WLS
Testicular cancer	DALYs	Latin America and Caribbean	2000–2009	1.344	1.260	1.429	0.000	0.975	10	WLS
Prostate cancer	DALYs	Latin America and Caribbean	2005–2009	-0.552	-0.736	-0.367	0.000	0.761	5	WLS
Prostate cancer	DALYs	Latin America and Caribbean	2010–2014	-1.408	-1.460	-1.356	0.000	0.996	5	WLS

Bladder cancer	DALYs	Latin America and Caribbean	2010–2019	-0.314	-0.410	-0.217	0.000	0.612	10	WLS
Kidney cancer	DALYs	Latin America and Caribbean	2010–2019	0.740	0.598	0.882	0.000	0.804	10	WLS
Testicular cancer	DALYs	Latin America and Caribbean	2010–2019	3.527	3.243	3.813	0.000	0.960	10	WLS
Prostate cancer	DALYs	Latin America and Caribbean	2015–2019	-0.581	-0.716	-0.446	0.000	0.869	5	WLS
Prostate cancer	DALYs	Latin America and Caribbean	2020–2023	-1.282	-1.847	-0.713	0.001	0.714	4	WLS
Bladder cancer	DALYs	Latin America and Caribbean	2020–2023	-0.641	-1.292	0.013	0.054	0.323	4	WLS
Kidney cancer	DALYs	Latin America and Caribbean	2020–2023	2.619	1.933	3.309	0.000	0.881	4	WLS
Testicular cancer	DALYs	Latin America and Caribbean	2020–2023	1.476	0.509	2.451	0.007	0.538	4	WLS
Prostate cancer	DALYs	High-middle SDI	2000–2004	-0.926	-0.989	-0.864	0.000	0.965	5	WLS
Bladder cancer	DALYs	High-middle SDI	2000–2009	-2.259	-2.410	-2.108	0.000	0.928	10	WLS
Kidney cancer	DALYs	High-middle SDI	2000–2009	0.329	0.252	0.406	0.000	0.517	10	WLS
Testicular cancer	DALYs	High-middle SDI	2000–2009	2.952	2.867	3.037	0.000	0.986	10	WLS
Prostate cancer	DALYs	High-middle SDI	2005–2009	-0.809	-0.921	-0.696	0.000	0.866	5	WLS
Prostate cancer	DALYs	High-middle SDI	2010–2014	-1.999	-2.043	-1.954	0.000	0.996	5	WLS
Bladder cancer	DALYs	High-middle SDI	2010–2019	-0.531	-0.712	-0.349	0.000	0.332	10	WLS
Kidney cancer	DALYs	High-middle SDI	2010–2019	-0.430	-0.507	-0.352	0.000	0.642	10	WLS
Testicular cancer	DALYs	High-middle SDI	2010–2019	5.381	5.265	5.498	0.000	0.992	10	WLS
Prostate cancer	DALYs	High-middle SDI	2015–2019	-0.871	-1.055	-0.688	0.000	0.738	5	WLS
Prostate cancer	DALYs	High-middle SDI	2020–2023	-0.145	-0.596	0.309	0.517	0.016	4	WLS
Bladder cancer	DALYs	High-middle SDI	2020–2023	1.718	1.051	2.391	0.000	0.521	4	WLS
Kidney cancer	DALYs	High-middle SDI	2020–2023	1.229	0.956	1.503	0.000	0.769	4	WLS
Testicular cancer	DALYs	High-middle SDI	2020–2023	5.101	4.247	5.962	0.000	0.858	4	WLS
Prostate cancer	DALYs	High-income North America	2000–2004	-3.224	-3.522	-2.924	0.000	0.987	5	WLS
Bladder cancer	DALYs	High-income North America	2000–2009	-0.051	-0.145	0.043	0.268	0.068	10	WLS
Kidney cancer	DALYs	High-income North America	2000–2009	-1.038	-1.164	-0.912	0.000	0.942	10	WLS
Testicular cancer	DALYs	High-income North America	2000–2009	-0.784	-1.088	-0.479	0.000	0.617	10	WLS
Prostate cancer	DALYs	High-income North America	2005–2009	-2.327	-2.623	-2.030	0.000	0.976	5	WLS
Prostate cancer	DALYs	High-income North America	2010–2014	-2.144	-2.611	-1.674	0.000	0.932	5	WLS
Bladder cancer	DALYs	High-income North America	2010–2019	-1.238	-1.452	-1.024	0.000	0.890	10	WLS
Kidney cancer	DALYs	High-income North America	2010–2019	-1.647	-1.848	-1.447	0.000	0.942	10	WLS
Testicular cancer	DALYs	High-income North America	2010–2019	0.691	0.179	1.205	0.011	0.310	10	WLS
Prostate cancer	DALYs	High-income North America	2015–2019	-0.806	-0.896	-0.716	0.000	0.981	5	WLS
Prostate cancer	DALYs	High-income North America	2020–2023	-0.725	-1.000	-0.450	0.001	0.874	4	WLS
Bladder cancer	DALYs	High-income North America	2020–2023	-0.567	-0.903	-0.231	0.006	0.739	4	WLS
Kidney cancer	DALYs	High-income North America	2020–2023	-2.014	-2.655	-1.368	0.000	0.905	4	WLS
Testicular cancer	DALYs	High-income North America	2020–2023	-1.349	-2.946	0.274	0.088	0.409	4	WLS
Prostate cancer	DALYs	High SDI	2000–2004	-1.525	-1.641	-1.409	0.000	0.948	5	WLS
Bladder cancer	DALYs	High SDI	2000–2009	-1.504	-1.535	-1.473	0.000	0.992	10	WLS
Kidney cancer	DALYs	High SDI	2000–2009	-0.610	-0.676	-0.545	0.000	0.813	10	WLS
Testicular cancer	DALYs	High SDI	2000–2009	-1.775	-1.815	-1.735	0.000	0.990	10	WLS
Prostate cancer	DALYs	High SDI	2005–2009	-1.828	-1.905	-1.752	0.000	0.984	5	WLS
Prostate cancer	DALYs	High SDI	2010–2014	-2.229	-2.282	-2.177	0.000	0.995	5	WLS
Bladder cancer	DALYs	High SDI	2010–2019	-1.290	-1.348	-1.231	0.000	0.961	10	WLS
Kidney cancer	DALYs	High SDI	2010–2019	-1.538	-1.573	-1.502	0.000	0.989	10	WLS
Testicular cancer	DALYs	High SDI	2010–2019	-0.363	-0.477	-0.250	0.000	0.341	10	WLS
Prostate cancer	DALYs	High SDI	2015–2019	-1.237	-1.299	-1.176	0.000	0.977	5	WLS
Prostate cancer	DALYs	High SDI	2020–2023	-0.364	-0.544	-0.183	0.000	0.360	4	WLS
Bladder cancer	DALYs	High SDI	2020–2023	0.222	0.001	0.443	0.049	0.123	4	WLS

Kidney cancer	DALYs	High SDI	2020–2023	-0.763	-0.938	-0.587	0.000	0.722	4	WLS
Testicular cancer	DALYs	High SDI	2020–2023	1.354	0.814	1.898	0.000	0.468	4	WLS
Prostate cancer	DALYs	Global	2000–2004	-1.144	-1.720	-0.566	0.008	0.929	5	WLS
Bladder cancer	DALYs	Global	2000–2009	-1.705	-1.865	-1.546	0.000	0.987	10	WLS
Kidney cancer	DALYs	Global	2000–2009	-0.371	-0.558	-0.183	0.002	0.721	10	WLS
Testicular cancer	DALYs	Global	2000–2009	-1.211	-1.507	-0.914	0.000	0.916	10	WLS
Prostate cancer	DALYs	Global	2005–2009	-1.221	-1.565	-0.877	0.002	0.977	5	WLS
Prostate cancer	DALYs	Global	2010–2014	-1.743	-1.883	-1.601	0.000	0.998	5	WLS
Bladder cancer	DALYs	Global	2010–2019	-0.977	-1.222	-0.731	0.000	0.912	10	WLS
Kidney cancer	DALYs	Global	2010–2019	-1.062	-1.146	-0.978	0.000	0.991	10	WLS
Testicular cancer	DALYs	Global	2010–2019	0.352	0.036	0.670	0.033	0.452	10	WLS
Prostate cancer	DALYs	Global	2015–2019	-0.760	-1.164	-0.353	0.010	0.922	5	WLS
Prostate cancer	DALYs	Global	2020–2023	0.072	-1.075	1.233	0.813	0.035	4	WLS
Bladder cancer	DALYs	Global	2020–2023	0.863	-1.068	2.831	0.196	0.646	4	WLS
Kidney cancer	DALYs	Global	2020–2023	-0.770	-1.770	0.240	0.082	0.844	4	WLS
Testicular cancer	DALYs	Global	2020–2023	1.459	-2.369	5.437	0.247	0.568	4	WLS

Figure S1. Forest Plot of Interval-Specific APCs for Prostate Cancer.

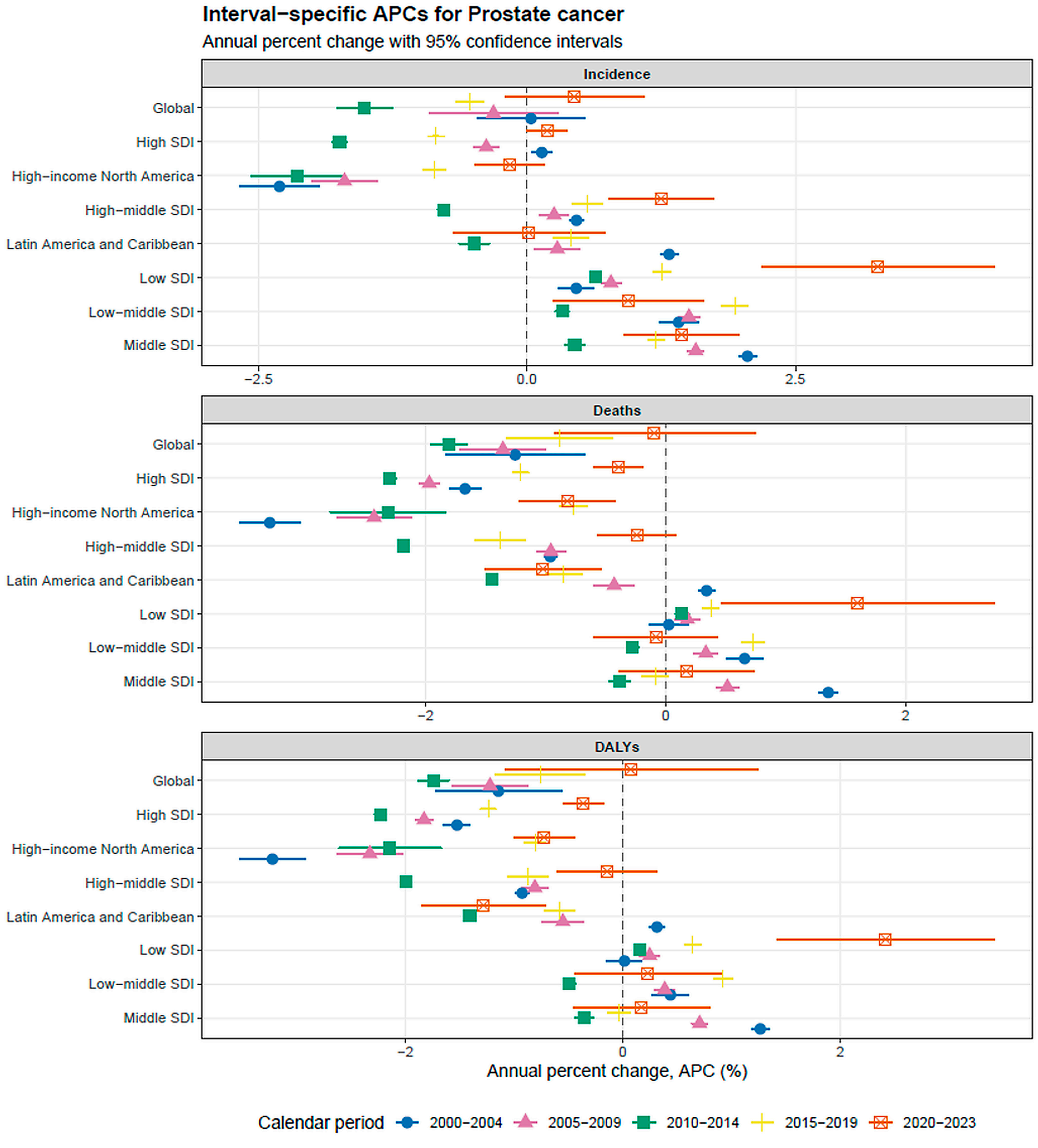

Figure S2. Forest Plot of Interval-Specific APCs for Testicular Cancer.

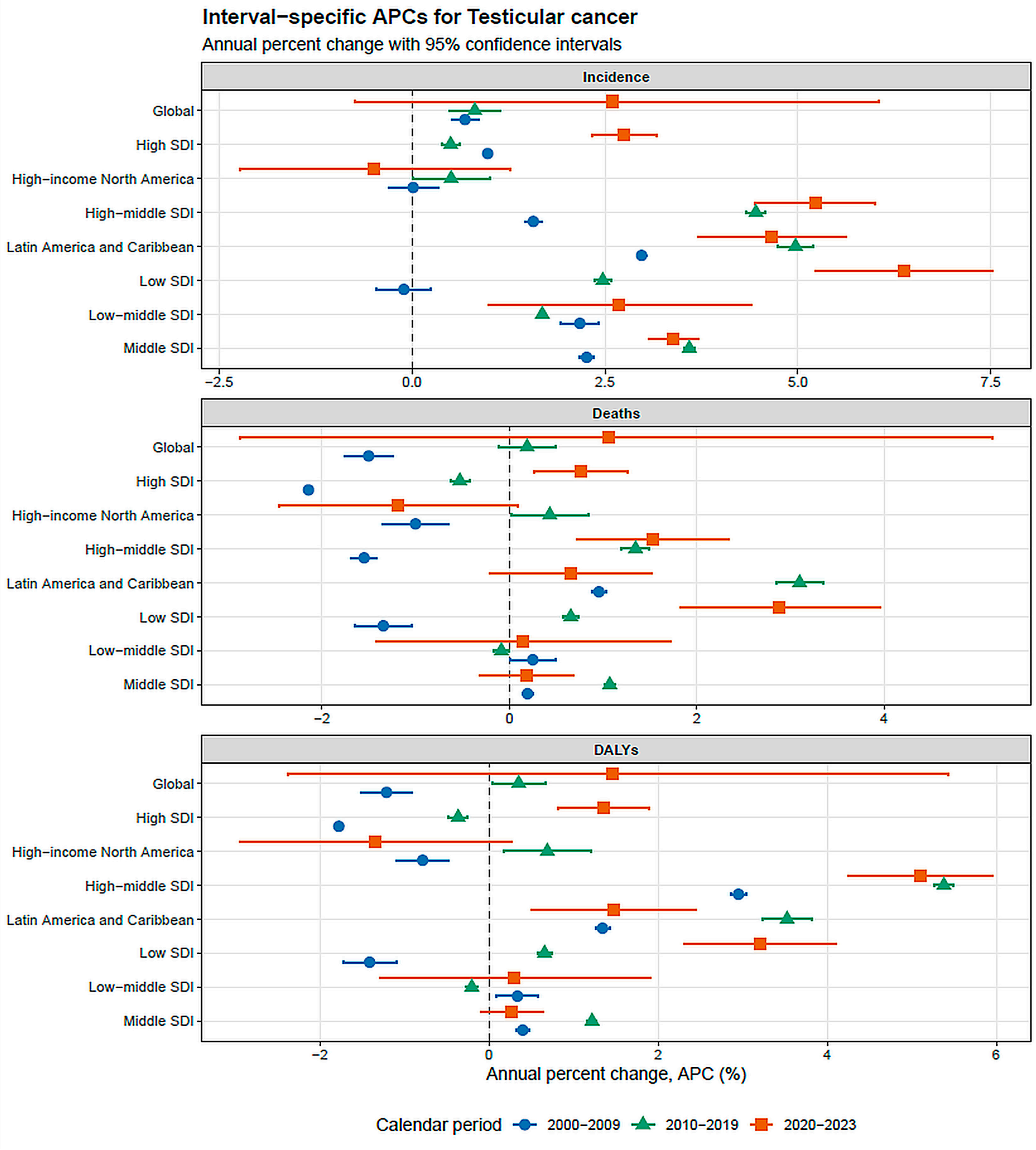

Figure S3. Forest Plot of Interval-Specific APCs for Kidney Cancer.

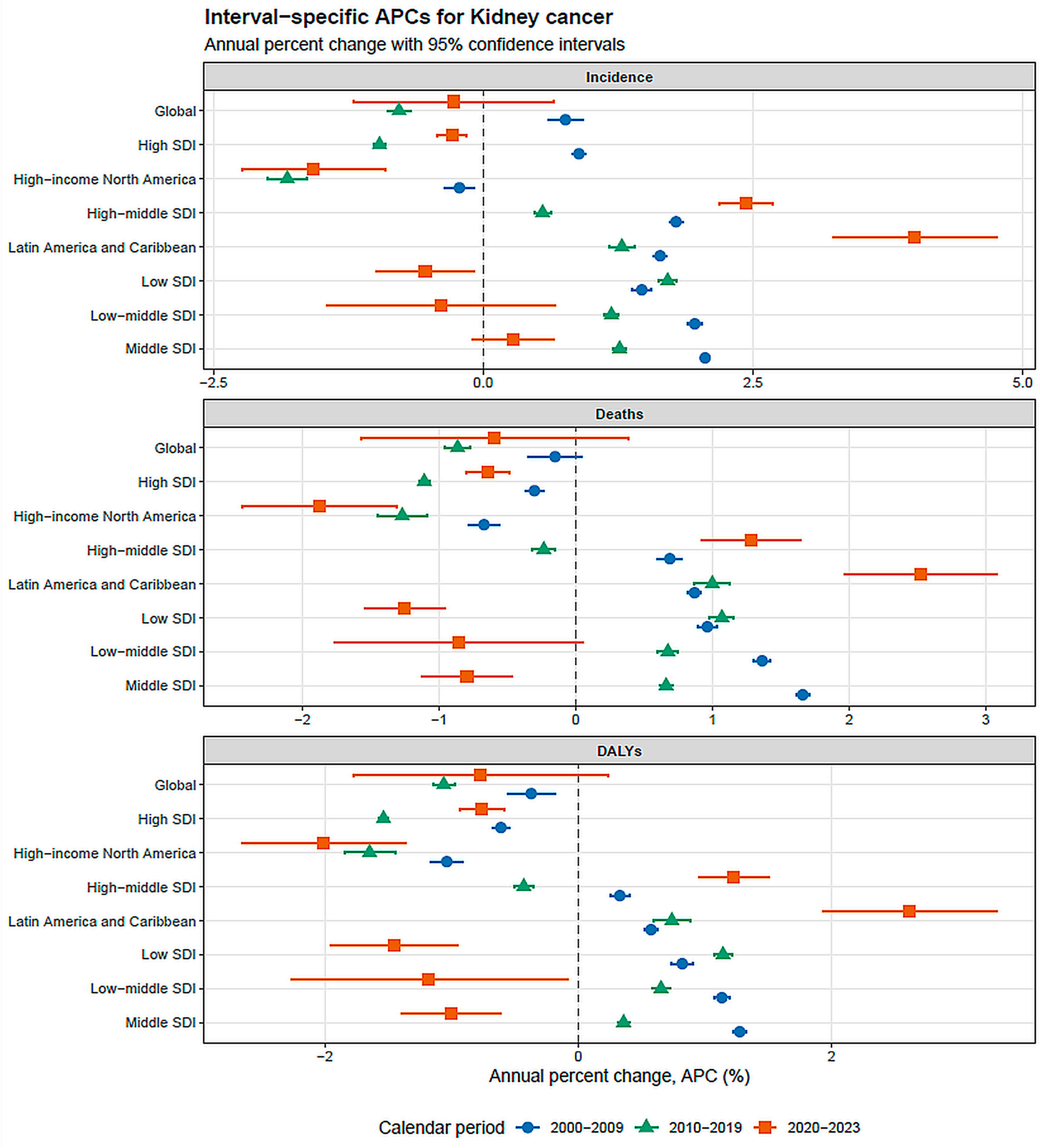

Figure S4. Forest Plot of Interval-Specific APCs for Bladder Cancer.

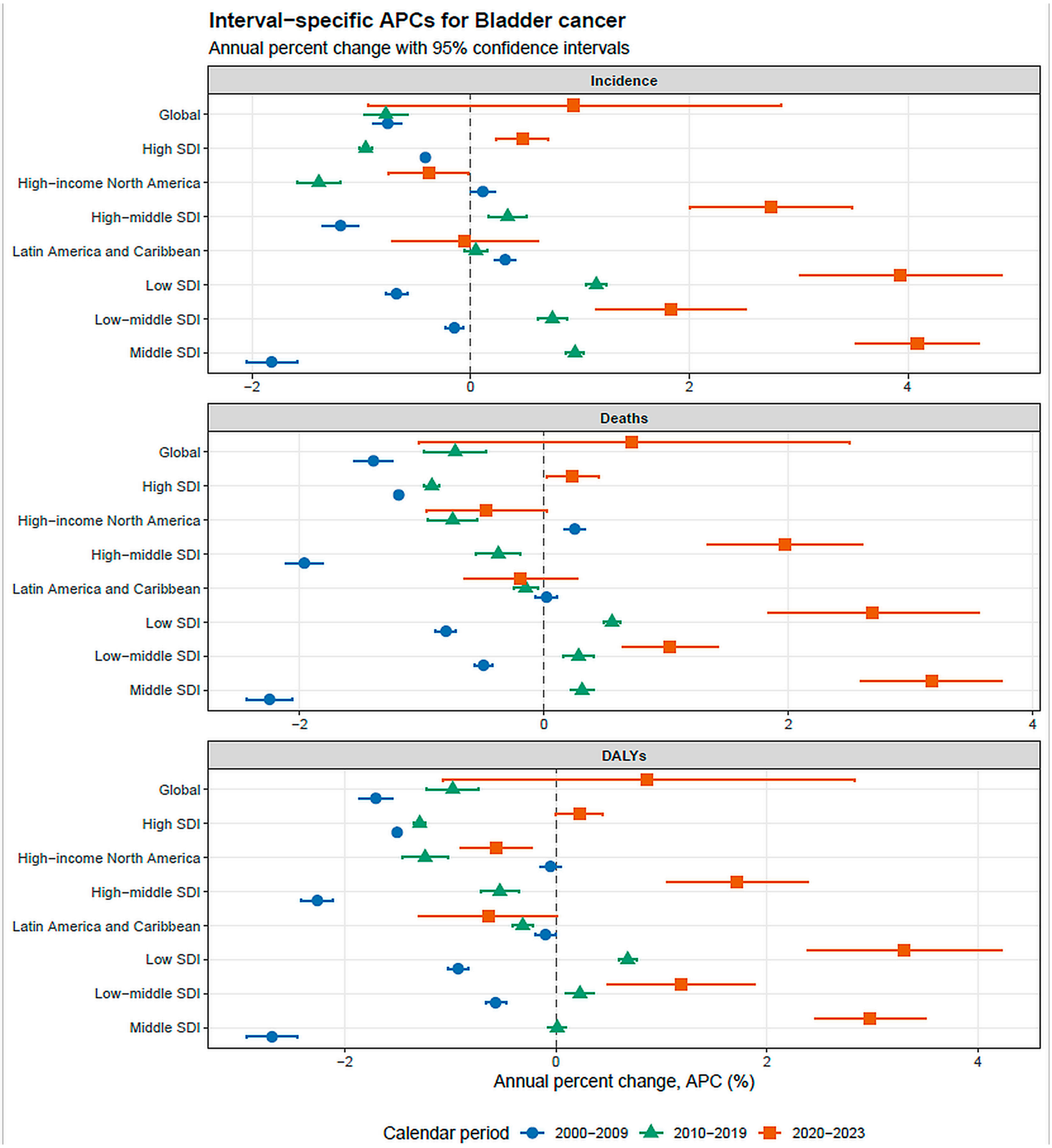

Supplement: Supplementary file 1 [file cancers-18-02016-s001.zip › cancers-4357827-supplementary.pdf]
